# Responding to the increase in substance use, HIV, HCV, and overdose in New England (RISE-NE): Protocol for a prospective cohort study

**DOI:** 10.1371/journal.pone.0358938

**Published:** 2026-09-25

**Authors:** Katie B. Biello, Angela R. Bazzi, Katherine Dunham, Jacqueline E. Goldman, Maxwell S. Krieger, Vanessa Silva, Yu Li, Deve Mehta, Mavis Nimoh, Chanelle J. Howe, Curt G. Beckwith, Devika Singh, Brandon D. L. Marshall

**Affiliations:** 1 Department of Epidemiology, Brown University School of Public Health, Providence, Rhode Island, United States of America; 2 Herbert Wertheim School of Public Health, University of California San Diego, La Jolla, California, United States of America; 3 Department of Community Health Sciences, Boston University School of Public Health, Boston, Massachusetts, United States of America; 4 Tapestry Health Systems, Springfield, Massachusetts, United States of America; 5 Division of Infectious Diseases, Brown University Health/ Warren Alpert Medical School of Brown University, Providence, Rhode Island, United States of America; 6 Department of Medicine, University of Vermont Medical Center, Burlington, Vermont, United States of America; Public Library of Science, UNITED KINGDOM OF GREAT BRITAIN AND NORTHERN IRELAND

## Abstract

**Background:**

High levels of opioid use, early introduction of drug supply contaminants, and increasing polysubstance use have led to intersecting epidemics of HIV, HCV, and overdose among people who use drugs (PWUD) in New England. Prevention services (e.g., syringe service programs, HIV testing) have been available in parts of New England for decades, yet significant service delivery gaps exist outside of major metropolitan areas. Further, racially minoritized PWUD face heightened discrimination in service settings and may be reluctant to access services. Our five-year, prospective cohort study, “Responding to the Increase in Substance Use, HIV, HCV, and Overdose in New England” (RISE-NE), will systematically examine rapidly evolving HIV and substance use trends in New England with the ultimate goal of informing the equitable scale-up of evidence-based HIV prevention and treatment services for PWUD.

**Methods:**

We will recruit 1,200 PWUD across Massachusetts, Rhode Island, and Vermont to participate in our cohort. Beginning at enrollment, participants will complete semiannual surveys asking about their substance use patterns, ability to access healthcare and social support services, and HIV, HCV, and overdose outcomes. Biospecimen collection (e.g., HIV/HCV testing, oral fluid collection, and toxicology testing) will also occur semiannually. All aspects of the project will be guided by community leadership and a multilevel framework for HIV, HCV, and substance use outcomes.

**Discussion:**

Using data from the RISE-NE cohort, we aim to: 1) examine regional transitions in substance use patterns and trajectories; 2) evaluate multilevel determinants of equitable access to and quality of services along the HIV, HCV, and overdose care continua; 3) evaluate the population-level causal effects of potential policy changes and interventions on our primary outcomes; and 4) rapidly address current and emerging research priorities. We discuss the anticipated strengths of this study as well as opportunities for training and dissemination.

## Introduction

Sizable HIV outbreaks are occurring among people who use drugs (PWUD) in New England, despite the presence of relatively robust harm reduction and substance use services in some communities [1–5]. From 2015 to 2018, a large HIV outbreak in two Massachusetts communities involved over 120 new HIV diagnoses attributed to injection drug use [3]. Similarly, from 2017 to 2018, a case cluster of 38 residents in Vermont were diagnosed with HIV infection with a substantial proportion engaged in recent methamphetamine use [5]. Elsewhere in the region, a 2023 outbreak in rural Maine involved over forty new HIV diagnoses, of which over 90% were associated with injection drug use, homelessness, and hepatitis C (HCV) coinfection [4]. Nationally, rates of HCV have also been increasing; while representing a small decrease from the year prior, the number of acute HCV cases in 2022 was double that of 2015 [6,7]. In 2023, Rhode Island, Massachusetts, and Vermont all had higher rates of acute HCV infection — a known precursor of HIV outbreaks — than the national average [7,8].

Multilevel factors are driving the escalation of HIV transmission in the context of drug use in New England, including frequent fentanyl injection [1,2], co-use of opioids and stimulants [9,10], and inaccessibility of syringe services programs (SSPs) [2,11]. For those who can access HIV treatment, lack of reliable transportation, incarceration, and homeless encampment sweeps often disrupt treatment engagement, particularly in suburban and rural areas [1,12]. These structural drivers of HIV transmission among PWUD are increasingly prevalent in the region: New England is experiencing rapidly rising levels of housing unaffordability, leading to unstable housing and homelessness [13–15].

New England is also experiencing an unprecedented overdose crisis, previously characterized by high levels of prescription opioid and heroin use and more recently driven by fentanyl and drug supply contaminants such as xylazine and medetomidine [16–20]. Research has shown that significant racial disparities exist in fatal overdose rates and, while rates of fatal overdose have been decreasing nationally and in New England since 2023, overdose rates among Black and Latino communities have increased [21–24].

Established evidence-based prevention services (e.g., SSPs, HIV/HCV testing and treatment, overdose education and naloxone distribution programs) have long been available in parts of New England [25,26]. The region has also piloted novel HIV, HCV, and overdose prevention strategies including universal access to medications for opioid use disorder (MOUD) in correctional settings [27], interventions to increase oral and long-acting injectable HIV pre-Exposure Prophylaxis (PrEP) uptake [12,28], drug checking services [29,30], and overdose prevention centers [31]. However, significant service delivery gaps and substantial geographic, racial, and ethnic inequities persist. Research has found that geographic and socio-political challenges suppress access to health and harm reduction services outside of major metropolitan areas [11,32,33]. Moreover, as a result of structural racism, Black, Hispanic/Latino, and Indigenous PWUD disproportionately experience barriers to accessing prevention services and are experiencing elevated levels of HIV, HCV, and overdose [24,34–40].

Several important research directions remain unexplored. First, there have been few observational cohort studies involving PWUD outside of the large metropolitan areas of the United States (U.S.), limiting our understanding of unique determinants of service access and quality in smaller and more suburban and rural communities [41]. Beyond studying evolving HIV, HCV, and overdose trends, there’s also a critical need to study emerging substance use trends and health outcomes in New England. For example, surveillance data from other parts of North America have highlighted elevated polysubstance use involving stimulants and widespread transitions from injection to non-injection routes of drug use [42–44]. Novel substances like xylazine and medetomidine have also been emerging in local New England drug supplies [19,20]. These trends create new health concerns for PWUD as well as challenges for the programs that serve them [45–47]. Additionally, given that many of these novel adulterants were first identified in drug supplies in the Northeast, this region can be viewed as a bellwether for drug supply evolution more broadly in the U.S. Finally, few existing studies have explored the *quality* of services along the HIV, HCV, and overdose care continua for PWUD. Given that negative and traumatic experiences in healthcare often lead to intentional avoidance of services among PWUD—including primary care [32,48], HIV/HCV services [49], mental healthcare [50], and MOUD [34,51]—a better understanding of the implementation and quality of these services is critical.

### Objectives

To inform the equitable scale-up of evidence-based services across New England and beyond, our cohort study, “Responding to the Increase in Substance Use, HIV, HCV, and Overdose in New England,” hereafter referred to as RISE-NE, aims to address these knowledge gaps by investigating rapidly evolving HIV and substance use trends across three states in this region. Using data from the RISE-NE cohort, we aim to:

1) Examine trends in rapidly evolving substance use patterns (e.g., drug types, frequency of use, routes of administration) and multilevel determinants (e.g., structural racism, stigma, isolation, urbanicity, transportation, housing insecurity) of substance use trajectories.
2) Evaluate multilevel determinants of equitable access to and quality of services along the HIV, HCV, and overdose care continua.
3) Estimate the population-level impacts of potential policy changes and interventions on our primary HIV, HCV, and overdose outcomes.
4) Rapidly address current and emerging research priorities, including the effect of HIV-associated comorbidities on care continua outcomes.

## Materials and methods

RISE-NE is a five-year, open prospective cohort study funded by the U.S. National Institute on Drug Abuse (NIDA) (U01DA063213). Data collection is expected to begin in August 2026 and subsequent data analysis and results dissemination will occur throughout the study period. RISE-NE is part of a consortium of novel and existing cohort studies investigating the intersections of substance use and HIV/AIDS (as well as other infectious diseases and related comorbidities) in the U.S [52]. The first of its kind in New England, RISE-NE will follow 1,200 PWUD across Massachusetts, Rhode Island, and Vermont.

### Study design & sample

#### Conceptual framework.

**Fig 1:**
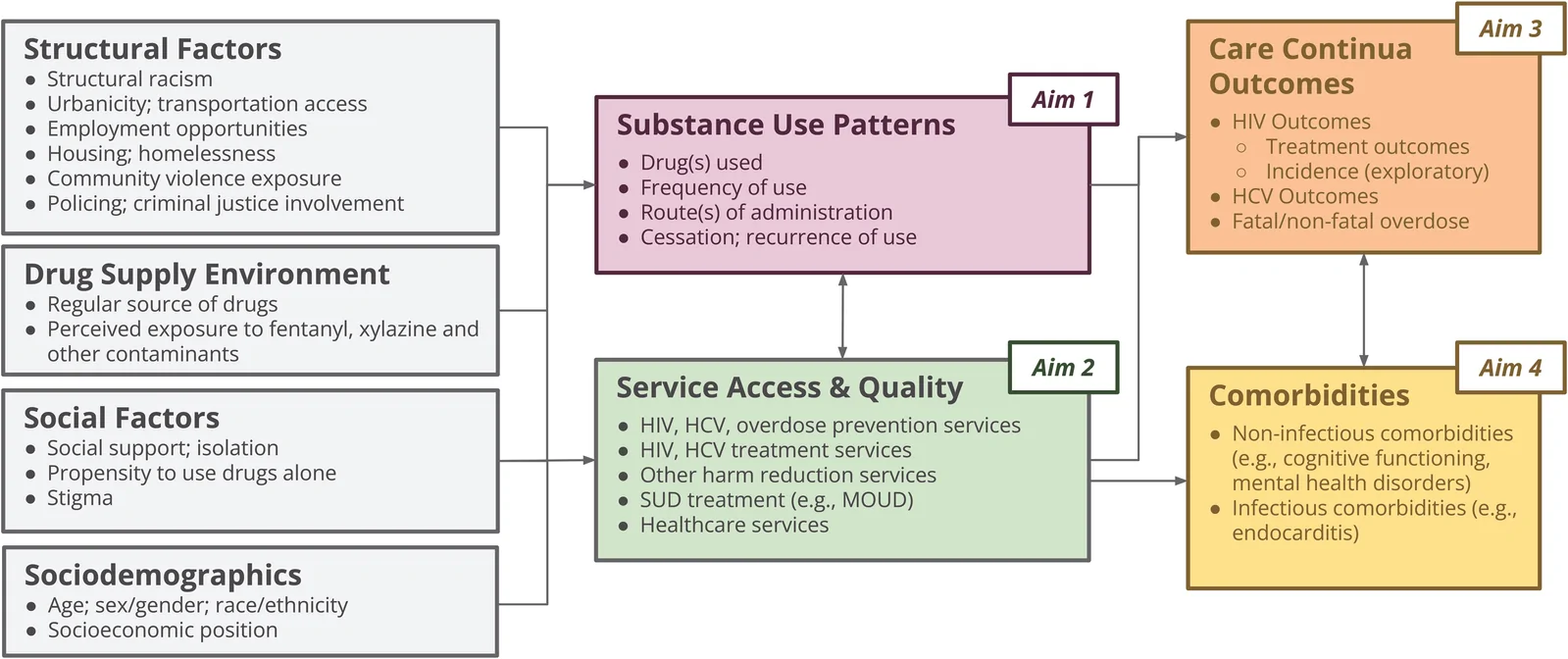
RISE-NE conceptual framework. The study’s conceptual framework, informed by Saloner’s framework for conceptualizing a public health approach to addressing opioid overdose, and aims [53].

All aspects of the project will be guided by Saloner’s framework for conceptualizing a public health approach to addressing opioid overdose [53], which itself draws on the socio-ecological model to depict multilevel influences on opioid-related outcomes [54,55]. For the purposes of RISE-NE, we extend this framework to focus on: (1) substance use patterns beyond opioids alone, (2) service access and quality, (3) the impact of specific services on HIV, HCV, and overdose outcomes, and (4) the role that comorbidities play in these care continua. As depicted in Fig 1, this framework will guide the collection of comprehensive prospective data on individual-level experiences (e.g., substance use patterns), social factors (e.g., social dynamics of use, stigma), and structural factors (e.g., structural racism, the drug supply environment) [53].

#### Community engagement & ethical considerations.

Our Community Leadership Council (CLC) provides feedback on all aspects of the project, including: recruitment and retention protocols, local considerations and priorities, appropriateness of proposed data collection measures, and eventual dissemination strategies. The CLC is currently co-chaired by one RISE-NE staff member and one community partner agency representative. Council members include people with lived/living experience, substance use/harm reduction providers, HIV care providers, and public health officials from all three states. The council meets quarterly and members are compensated for their time and expertise.

All study procedures have been reviewed and approved by the Brown University Institutional Review Board (IRB), which serves as the single IRB of record.

#### Study setting.

**Fig 2:**
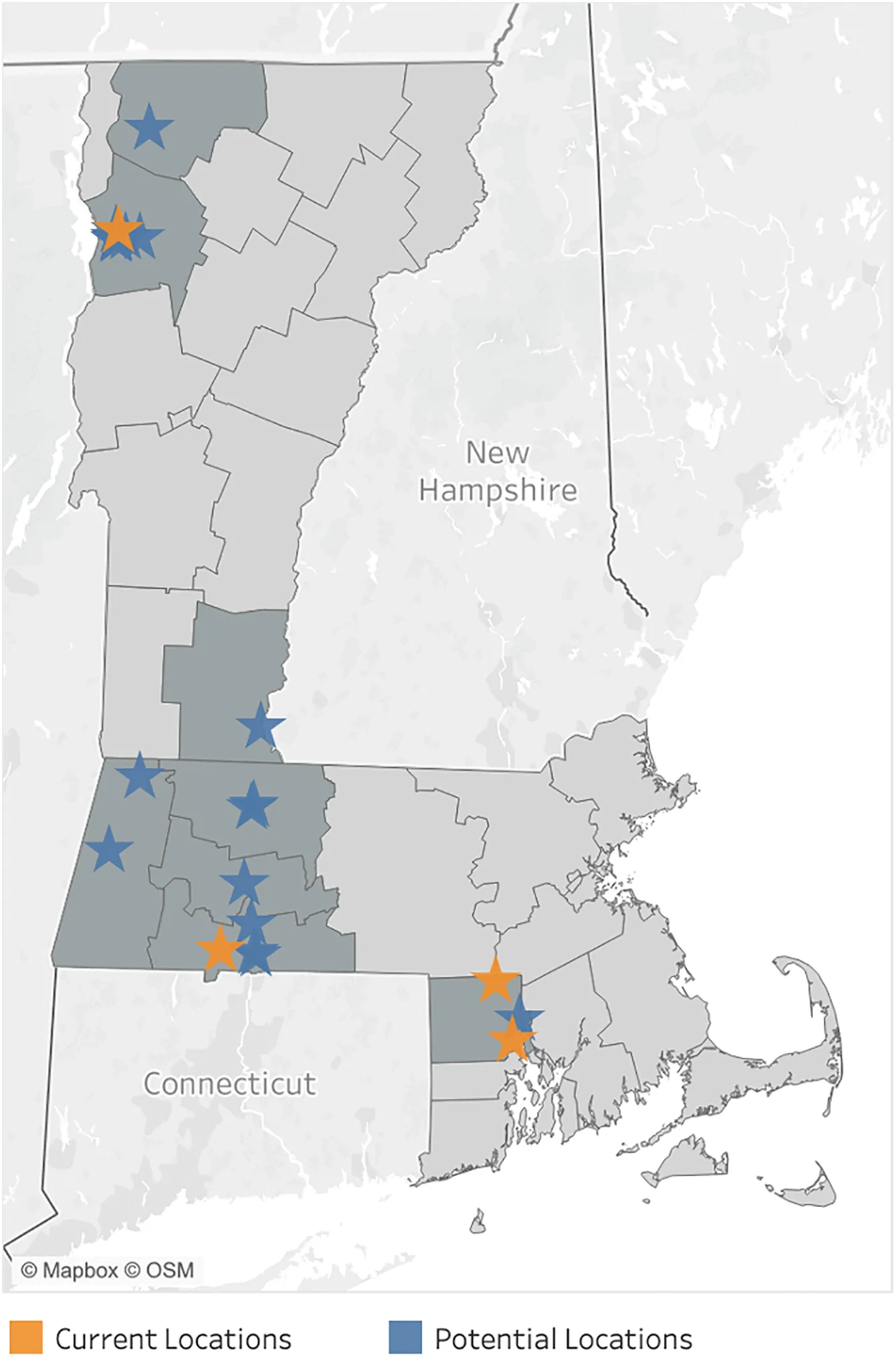
Current and future RISE-NE recruitment sites. Potential community partner agencies are located across eight different counties in MA, RI, and VT. Currently approved recruitment sites are shown in orange; potential future sites are shown in blue. Authors used Mapbox™ and OpenStreetMap™ software to generate this figure.

We will work with community partner agencies serving PWUD and people living with HIV (e.g., syringe services programs, harm reduction agencies, infectious disease clinics) across multiple areas of Massachusetts, Rhode Island, and Vermont, with a particular focus outside of major metropolitan areas like Boston (Fig 2).

#### Recruitment & eligibility.

Enrollment is planned to begin in August of 2026, with follow-up expected to continue through at least the end of 2029. We aim to recruit 1,200 PWUD across the three states shown in Fig 2, oversampling participants living with HIV (n = 180 target sample size, 15% of the total). We have conducted power calculations for a variety of analyses (purposefully choosing rare outcomes and conservatively estimating attrition) to ensure that this total sample size will allow us to detect small and medium effect sizes. Importantly, these calculations indicate that an enriched sample of ≥180 people living with HIV will allow for nuanced inferences within this subgroup. RISE-NE is an open prospective cohort design, such that we will continually recruit participants throughout the study period to maintain an active enrollment of n = 1,200. Trained research assistants (RAs) will carry out field-based recruitment at the project’s community partner sites. In addition, the team will engage in outreach strategies in wider communities to recruit PWUD who may not be regularly receiving services from community-based organizations. Such strategies will include snowball sampling and canvassing areas where PWUD are known to spend time, as informed by local community partners and the CLC.

Potential participants will complete structured pre-screeners to assess eligibility. Individuals will be eligible for enrollment in RISE-NE if they: (1) are 18 years of age or older; (2) report injection, insufflation (i.e., snorting), and/or inhalation of drugs in the past 30 days; and (3) are able to understand and speak English or Spanish. Individuals will be ineligible if they: (1) only report cannabis, tobacco, vaping, or alcohol use in the past 30 days; (2) live outside of Massachusetts, Rhode Island, or Vermont, or plan to move out of state within the next year; or (3) are unable to provide informed consent. RAs will obtain informed consent from interested and eligible participants in private areas at our field sites.

### Data collection

#### Semiannual surveys.

Beginning at enrollment, participants will complete semiannual surveys that will be administered by RAs using REDCap. The survey instrument incorporates common data elements from the PhenX Toolkit, other NIDA data harmonization efforts, and previous work conducted by the study team [31,56–59]. Measures were also reviewed by and adapted with input from our CLC, study staff, and potential participants. A summary of the enrollment survey’s key domains and topics is provided in Table 1. Briefly, key Aim 1 variables include self-reported substance use measures; key Aim 2 variables include self-reported access to and quality of care; key Aim 3 variables include self-reported engagement with HIV, HCV, and overdose care continua; and key Aim 4 variables include self-reported presence of comorbidities. More detailed information about key variables and instrument sources is included in S1 File and the complete baseline survey is included in S2 File. Note that the content of the survey is subject to change based on evolving research priorities, feedback from CLC members and recruitment sites, and other factors.

**Table 1:** Key assessment domains of the RISE-NE enrollment survey.

Domains	Key Topics
Demographics	Basic demographic information, housing stability, socioeconomic position
Substance Use	Drug use, stigma toward drug use, overdose experiences, harm reduction practices and service use, substance use disorder treatment, HCV prevention and treatment
Sexual Health	STI testing, HIV testing and prevention, HIV treatment, stigma toward HIV, sexual partners, condom use, sex work
Health	Overall health status, quality of life, healthcare access and quality, comorbidities, pregnancy experiences, oral health, mental health, proactive personality traits
Social and Structural Determinants of Health	Material hardship, transportation access, criminal justice system involvement, exposure to violence, social cohesion, discrimination

Participants who complete the semiannual survey assessments and biospecimen collection will be compensated $80 cash for their time. To promote participant retention over the study’s follow up period, full-time RAs will be embedded within partner organizations, allowing our staff to flexibly schedule study visits as well as gain familiarity and build rapport with participants, service providers, and broader communities over time.

#### Monthly retention surveys.

Each month, RAs will contact participants to briefly confirm and/or update their contact information and ask about a rotation of key study outcomes (e.g., past 30-day harm reduction and medical service use, overdose incidence, overamp incidence). These 5–10 minute “check-in” surveys can be completed by phone or at participating community-based sites and participants will be compensated $5 cash for their time.

#### Semiannual biospecimen collection.

Biospecimen collection (e.g., HIV/HCV testing and labs) will also occur semiannually over the study period. A summary of these samples is provided in Table 2. Whole blood collection will be administered by a trained phlebotomist at participating organizations where recruitment and enrollment occur. We will use rapid tests to conduct serial rapid HIV and HCV antibody testing for those not already diagnosed. The FDA-approved OraQuick® ADVANCE^TM^ HIV-1/2 Antibody Test will be used for HIV testing among participants and the FDA-approved OraQuick® HCV test will be used for HCV testing among participants. HIV and HCV test results will be returned to participants by trained RAs on the same day and participants who test positive will be referred to additional services, given that rapid HIV and HCV tests are screening tests that require confirmatory testing by a trained medical provider. Specifically, participants who receive a reactive test result will receive support and referrals for confirmatory testing, HIV care, and other services as applicable. Whole blood samples will also be used to measure HIV and HCV viral load (i.e., to confirm virological response among those on treatment) and to conduct semiannual toxicology screens to assess the presence and levels of opioids (e.g., heroin, methadone, morphine), stimulants (e.g., methamphetamine, cocaine), benzodiazepines, and hallucinogens.

**Table 2:** Biospecimen overview.

Biospecimen Collection Method	Sample Purpose
Venipuncture	HIV antibody test (via OraQuick® ADVANCE^TM^ HIV-1/2 Antibody Test)
	HCV antibody test (via OraQuick® HCV test)
	HIV viral load
	HCV viral load
	Comprehensive drug screen
	Plasma storage
Saliva	HIV antibody test (if needed)
	Oral health assessment (via SalivaBio’s Saliva Collection Aid)

In some events, when venipuncture is not available, oral-fluid specimens will be obtained for HIV antibody testing. Saliva samples will also be collected to assess biomarkers related to tooth and gum health.

#### Geospatial and administrative data linkages.

In accordance with our multilevel framework, we will assess a variety of neighborhood-level factors as social and structural determinants of health and will operationalize these exposures at the U.S. Census tract level. Census tracts will be our primary structural-level geographic measure because they are designed to approximate neighborhoods and are consistently defined across urban, suburban, and rural regions. During the enrollment survey and every six months thereafter, participants will be asked to pinpoint on a map where they spend most days and most nights, respectively), which will then be geocoded to census tracts using ArcGIS Pro 3.5.0 (ESRI Inc.). We will obtain data from the U.S. Census Bureau (i.e., American Community Survey 5-year estimates) to construct variables for selected structural factors (e.g., urbanicity, disadvantage, structural racism) [60–62]. Finally, we will link study participants to the National Center for Health Statistics’ National Death Index to ascertain cause-specific and all-cause mortality [63].

#### Data management and safety.

All data management and sharing activities will comply with NIDA’s HIV Cohorts Program and Data Coordinating Center guidelines. All study data will be entered securely at field sites on laptops using a RISE-NE-specific, HIPAA-compliant REDCap instance within Brown University’s secure computing and storage environment. All study data will be encrypted and backed up daily on centralized, secure servers at Brown University. Biospecimens will be shipped from each respective enrollment site to The Miriam Hospital in Providence, Rhode Island where laboratory staff will process, aliquot, freeze, and store samples long-term. Samples will be de-identified and stored for additional testing to support supplemental and emerging scientific questions within NIDA’s HIV Cohorts Program. All biospecimen values will be transferred securely and linked to survey data using unique participant ID numbers.

### Data analyses

While the final analytic techniques used in this study will ultimately depend on the structure of our final data, our current analytic plan is as follows.

#### Aim 1: Trends and trajectories of substance use.

To examine trends and trajectories in substance use patterns, we will first analyze trends over time, stratifying by subgroups of interest (e.g., race/ethnicity). Next, we will use person-time methods to evaluate the cumulative incidence of key substance use outcomes in relevant subgroups (e.g., injection cessation among those injecting at enrollment, recurrence of use). Third, we will use survival analysis methods to estimate associations of individual- and census tract-level exposures of interest (e.g., housing insecurity) on key substance use outcomes (e.g., non-fatal overdose) [64,65]. Fourth, we will use multi-state, time-homogeneous Markov models to assess transitions between discrete substance use states and longitudinal patterns of substance use (e.g., we will estimate the probability of transitioning from active use to early cessation, then cessation to recurrence of use) [66–70]. Fifth, we will employ repeated measures latent class analysis to explore longitudinal polysubstance use trends [71]. Finally, to evaluate the multilevel determinants of substance use trajectories, we will use multilevel growth mixture models [72,73], an extension of growth mixture models that identify latent “clusters” of trajectory patterns and heterogeneity in individual trajectories over time [74]. We will develop trajectories for multiple substance use outcomes (e.g., frequency, routes of use) and identify “latent” trajectories representing distinct patterns over time.

#### Aim 2: Equitable access to and quality of services.

Aim 2 analyses will be largely predictive in nature. To describe patient-centered quality measures and HIV, HCV, and overdose care continua outcomes over time in the overall sample and by race/ethnicity, we will first use visual and descriptive methods. We will examine racial/ethnic inequities in care continua outcomes using the *g*-formula and informed by the target study conceptual model for measuring disparities [75,76]. Second, we will characterize probabilities of transition between care continua stages using multi-state Markov transitions models and examine predictors of key transition probabilities and stages [77]. Multilevel mixed-effects models will identify individual- and census tract-level predictors of key care continua outcomes over time [78].

#### Aim 3: Population-level impacts of potential policy changes and interventions.

We will be leveraging RISE-NE’s observational data and advanced causal inference methods to simulate the population‐level effects of simultaneously scaling established services and implementing novel interventions on primary outcomes. We are particularly interested in estimating the joint effects of scaling existing services and implementing novel interventions on our care continua outcomes. We will use the *g*-formula to estimate the population risk for outcomes of interest in various hypothetical intervention scenarios [76]. For example, we will estimate the effect of hypothetical joint interventions to improve MOUD access and implement novel harm reduction interventions (e.g., overdose prevention centers) on overdose mortality rates. In contrast to typical regression techniques, the *g*-formula is able to estimate the effects of more realistic interventions (e.g., instead of an entire population receiving the joint intervention, only a subset does), particularly in the context of time-varying confounding [76,79–81]. The final outcomes of such models are similar to population attributable risks (i.e., the expected population-level change in a particular outcome that might be observed by intervening on one or multiple exposures).

#### Aim 4: Effects of HIV-associated comorbidities on care continua outcomes.

Using similar techniques as in Aim 2 (i.e., mixed effects regression), we will evaluate whether comorbidities influence care continua outcomes. However, in contrast to Aim 2, this aim will be descriptive and causal in nature, rather than predictive. We will also consider the presence of comorbidities as possible effect measure modifiers in associations between receipt of services and care continua outcomes.

Our analysis plans are likely to evolve over time and future publications will describe any changes to our analysis plan. Given that a central aim of the study is to explore new and emerging drug use patterns and research priorities, we anticipate that new research questions and lines of inquiry will arise over the course of this study. We have, therefore, designed RISE-NE to be flexible and adaptable to these new and emerging needs.

## Discussion

RISE-NE represents an unprecedented opportunity to prospectively investigate the health and well-being of PWUD across three New England states that have been heavily affected by the HIV, HCV, and overdose crises. Using rich prospective data and state-of-the-art causal inference methods, our findings on the joint impact(s) of multiple interventions on key health outcomes can inform the equitable scale-up of evidence-based services within and beyond the study region.

RISE-NE will offer multiple contributions to our understanding of evolving HIV and substance use trends as well as the service needs and health priorities of PWUD. First, RISE-NE will be the first HIV and substance use cohort of this scale in New England, a region heavily impacted by unprecedented levels of polysubstance use and related health consequences including HIV, HCV, overdose, and comorbidities (e.g., mental health disorders). Furthermore, RISE-NE will be the largest and most comprehensive prospective cohort study ever conducted outside of a major U.S. metropolitan area, providing critical insights into evolving substance use trends and service landscapes among diverse and understudied geographic areas [41]. Second, in addition to examining rapidly evolving substance use trends and health outcomes, RISE-NE has a novel emphasis on examining access to and quality of services along the HIV, HCV, and overdose care continua. Our conceptual framework and multilevel measures enable breadth and depth in the evaluation of equitable access to and quality of both well-established prevention services and novel intervention strategies. Third, we will use rigorous, state-of-the-art causal inference methods to estimate the effects of joint, policy-relevant, and realistic interventions on health outcomes. Finally, cutting across all these innovations, RISE-NE places local, community voices at the forefront to ensure that our objectives and findings will be useful and relevant for local resource allocation and programmatic decisions.

We expect that results from the study will be available during and after data collection. To maximize the reach and ultimate impact of RISE-NE, we will disseminate findings in an equitable, inclusive, and ongoing manner to a range of scientific and community audiences. These dissemination targets include publications, conferences, NIDA’s Data Coordinating Center and other data repositories, community presentations, data dashboards, and other formats requested by our CLC, community partner agencies, health departments, and other key end-users. Notably, data harmonization among NIDA’s HIV SUCCESS Cohorts will allow for increased data sharing and opportunities for national comparisons. We will regularly invite CLC members to contribute to the design and implementation of our dissemination activities, including participation as co-authors and co-presenters across all years of the project. This will both help to ensure the use of appropriate language and accurate interpretation of our findings and maximize the reach and utility of our findings for end-users.

Finally, we aim to leverage the RISE-NE infrastructure as a training platform by engaging a cohort of trainees who span disciplines and career stages (i.e., undergraduates, pre- and postdoctoral trainees, early-career faculty). In addition to mentoring academic trainees, we will regularly provide technical assistance and mentorship opportunities to community members to build research capacity.

By leveraging rigorous longitudinal data collection protocols, causal inference methods, and community engagement strategies, RISE-NE will provide critical insight into evolving health outcomes and service needs among PWUD in New England. These insights and results will have practical implications for local PWUD, service providers, researchers, and policymakers. Ultimately, RISE-NE is an opportunity to investigate long-unanswered questions about the health of PWUD as well as identify new and emerging priorities for the future.

## Supporting information

S1 File Summary of survey domains, item counts, and instrument sources at enrollment. (DOCX)

S2 File Complete enrollment survey, as of manuscript submission. (PDF)
