# Supplementary material for: Responding to the increase in substance use, HIV, HCV, and overdose in New England (RISE-NE): Protocol for a prospective cohort study
Source: PLoS One. 2026 Sep 25;21(9):e0358938. doi: 10.1371/journal.pone.0358938 (PMC13614656; doi:10.1371/journal.pone.0358938)
Supplement: Supplementary file 1 [file pone.0358938.s002.pdf]

IRB Attachment 1a. Enrollment and Baseline Survey

Co-Principal Investigators:

Angela Bazzi, PhD
Katie Biello, PhD
Brandon DL Marshall, PhD

These research materials are the property of the principal investigators and are not to be shared or used outside of the study without specific and written permission of the co-Principal Investigators, Angela Bazzi, abazzi@health.ucsd.edu, Katie Biello, PhD, katie_biello@brown.edu, and Brandon DL Marshall, PhD, brandon_marshall@brown.edu.

Table of Contents

**Demographics Block**	**3**
Demographics	3
Socioeconomic Position	7
**Substance Use Block**	**14**
Drug & Alcohol Use	14
Unexpected Drugs/Cuts	29
Substance Use Disorders	34
Drug Use Stigma	37
Overdose	38
Harm Reduction	43
SUD Treatment	51
HCV Prevention & Treatment	66
**Sexual Health Block**	**71**
STI Testing	71
HIV Testing and Prevention	74
HIV Care Cascade	84
Sexual Risk Behaviors	92
**Health Block**	**96**
Health Status and Quality of Life	96
Healthcare Access	99
Other Comorbidities	104
Pregnancy & Parenting	112
Oral Health and Access to Dental Services	117
Mental Health Disorders	122
**Structural & Psychosocial Block**	**125**
Material Hardship	125
Loneliness	126
Everyday Discrimination Scale	127
Transportation Insecurity	129
Criminal Justice System Involvement	130
Perceived Neighborhood Social Cohesion	133
Proactive Personality Scale	134
**Participant Screening: Script**	**135**
**Participant Screening: Questions**	**136**
**Participant Contact and Scheduling**	**138**

Demographics Block

Demographics

Prompt: Now I am going to ask you a few questions about you and your background.

DEM_1. How old are you (in years)? *(Write in #):* ____________
1. Don't know (997)
2. Prefer not to say (999)

DEM_2. What is the highest level of education you have completed?
1. 8th grade or less
2. Some high school (9th to 11th grade)
3. High school graduate (12th grade) or received a GED
4. Some college or technical training *(Note: e.g., trade school)*
5. College graduate or higher
6. Don't know (997)
7. Prefer not to say (999)

DEM_3. What is your gender? *(Interviewer note: if respondent gives multiple options (e.g., 3, 4, 5), use option 6 and write out)*
1. Man
2. Woman
3. Gender-queer / Nonbinary
4. Gender variant
5. Questioning or unsure of my gender identity
6. Something else: ______
7. Don't know (997)
8. Prefer not to say (999)

If DEM_3 ! = Something else (6), then skip to DEM_4;
Else ask

DEM_3a: Something else: ______ *(write in)*

DEM_4. Do you identify as transgender?
1. Yes
2. No
3. Don't know (997)
4. Prefer not to say (999)

DEM_5. What is your sexual orientation? *(Interviewer note: if respondent gives multiple options (e.g., 3, 4), use option 5 and write out)*
1. Straight/Heterosexual
2. Gay/Lesbian/Homosexual
3. Bisexual
4. Pansexual
5. Something else: ______
6. Don't know (997)
7. Prefer not to say (999)

If DEM_5 ! = Something else (5), then skip to DEM_6;
Else ask

DEM_5a: Something else: ______ *(write in)*

DEM_6. What is your current relationship status?
1. Married
2. Widowed
3. Divorced
4. Never married
5. Common law marriage/civil union
6. Living with main partner/ domestic partner
7. Separated
8. Other: _____________
9. Prefer not to say (999)

If DEM_6 ! = Other (8), then skip to DEM_7;
Else ask

DEM_6a: Other: ______ *(write in)*

DEM_7. During the **last 6 months**, on average, which of the following best describes the people you stay with? *(Select one)*
1. Just me
2. My spouse, partner, or significant other and not kids
3. Family and kids
4. My parents without kids
5. Family with 3 generations (parents, children, grandparents)
6. Roommates
7. Friends
8. Living with acquaintances or people I do not know well
9. Other: _____________
10. Prefer not to say (999)

If DEM_7 ! = Other (9), then skip to DEM_8;
Else ask

DEM_7a: Other: ______ *(write in)*

DEM_8. Were you born in the United States or in a U.S. territory (like Puerto Rico)?
1. Yes
2. No
3. Don't know (997)
4. Prefer not to say (999)

If DEM_8 = Don't know (997) (3) or Prefer not to say (999) (4), skip to DEM_9;
If DEM_8= No (2), skip to DEM_8b;
Else ask

DEM_8a. What state [or territory] were you born in? _______________ *(Select from drop down)*
1. Don't know (997)
2. Prefer not to say (999)

If DEM_8 = Yes (1), skip to DEM_9;
Else ask

DEM_8b. What country were you born in? __________________ *(Select from drop down)*
1. Don't know (997)
2. Prefer not to say (999)

DEM_9. How long have you lived here (in ***[Massachusetts/Rhode Island/Vermont]***)?
1. Entire life
2. Under 1 year
3. 1-2 years
4. 2-5 years
5. 5-10 years
6. 10+ years
7. Don't know (997)
8. Prefer not to say (999)

DEM_10. Do you consider yourself to be Afro-Latino/a, Hispanic, or Latino/a or Latine?
1. Yes
2. No
3. Don't know (997)
4. Prefer not to say (999)

DEM_11. How would you describe your racial background? *(Select all that apply)*
1. Black or African American
2. White
3. Native American/Alaska Native
4. Caribbean
5. Middle Eastern/North African (e.g., Iranian, Egyptian)
6. South Asian
7. Southeast/East Asian
8. Native Hawaiian or Pacific Islander
9. Biracial/Multiracial (unspecified)
10. Something else: ______
11. Don't know (997)
12. Prefer not to say (999)

If DEM_11 ! = Something else (10), then skip to DEM_12;
Else ask

DEM_11a: Something else: ______ *(write in)*

DEM_12. Do you speak a language other than *[current survey language (English/Spanish)]* at home?
1. Yes
2. No
3. Don't know (997)

4. Prefer not to say (999)

DEM_12a. What language(s) do you speak at home? *(Do not read list out loud, wait for respondent answer) (Select all that apply)*

1. English
2. Spanish
3. Afrikaans
4. Albanian
5. Arabic
6. Bengali
7. Bulgarian
8. Cantonese
9. Danish
10. French
11. French (Cajun)
12. Fulani
13. German
14. Greek
15. Haitian Creole
16. Hebrew
17. Hindi
18. Hungarian
19. Italian
20. Japanese
21. Kiswahili
22. Korean
23. Kru, Ibo, Yoruba
24. Mandarin
25. Mande
26. Polish
27. Portuguese
28. Punjabi
29. Russian
30. Serbo-Croatian
31. Tagalog
32. Telugu
33. Turkish
34. Urdu
35. Vietnamese
36. Yiddish
37. Other_________
38. Don't know (997)
39. Prefer not to say (999)

If DEM_12a ! = Other (37), then skip to SEP_1;
Else ask

DEM_12a1: Other: ______ *(write in)*

Socioeconomic Position

Prompt: Now I have some questions about your current housing, employment, and income.

SEP_1: Thinking about the **last month**, in what type of place did you sleep or stay *most nights*? (Please choose the one option that best describes where you stayed most often).

Interviewer note: If the participant stayed in more than one place, ask them to choose the place where they stayed the greatest number of nights. If it is truly tied, choose the place where they stayed most recently or consider their main place. As the interviewer you do not need to read out all response options and these definitions (unless it is necessary for the participant's understanding)

1. **Your own place** *(Your own apartment, house, or room where you are on the lease or have a long-term arrangement)*
2. **Someone else's place** *(Staying with friends, family, or a partner; couch surfing; not your own lease)*
3. **Temporary lodging** *(Hotel, motel, SRO, or rooming house paid day-to-day or week-to-week)*
4. **Shelter or transitional housing** *(Emergency shelter, warming center, transitional housing, domestic violence shelter)*
5. **Unsheltered / outdoors** *(Street, park, encampment, abandoned building, vehicle, or transit station)*
6. **Institutional setting** *(Jail, prison, detention center; hospital or psychiatric hospital; detox or residential treatment)*
7. **Other (please specify): _________**
8. Don't know (997) / not sure
9. Prefer not to say (999)

If SEP_1 ! = Other (7) skip to SEP_1a;
Else ask

SEP_1a1: Other: ______ *(write in)*

SEP_2: In the **last month**, did you spend any nights sleeping outside (like on the street, in a park, in a tent or encampment), in a vehicle, in an abandoned building, or in a transit station?

1. 1 = Yes
2. 0 = No
3. 7 = Don't know (997) / not sure
4. 9 = Prefer not to say (999)

SEP_3: Where do you spend most of your **days** over the **last month**?
(Interviewer: Open Google Maps in another tab. Turn the device to face the respondent and show them the map. Assist them with using the mobile interface to drop a pin, if needed. Right click the location on the map and select the GPS coordinates to copy to clipboard. Paste the coordinates below.)

1. Location: ______________ *(write in)*
2. Don't know (997)
3. Prefer not to say (999)

[in REDCap, program answer choices as:
1. Select to enter location
2. Don't know (997)
3. Prefer not to say (999)

SEP_4: Where have you spent most of your **<u>nights</u>** over the **<u>last month</u>**?
(Interviewer: Open Google Maps in another tab. Turn the device to face the respondent and show them the map. Assist them with using the mobile interface to drop a pin, if needed. Right click the location on the map and select the GPS coordinates to copy to clipboard. Paste the coordinates below.)

1. Location: ______________ *(write in)*
2. Don't know (997)
3. Prefer not to say (999)
   [in REDCap, program answer choices as:
   1. Select to enter location
   2. Don't know (997)
   3. Prefer not to say (999)

Prompt: Now for the next set of questions, I will ask you to think about the <u>last 6 months</u>.

SEP_5: Over the **<u>last 6 months</u>**, what is the longest amount of time you have lived in the same place?

1. One month or less
2. Two to three months
3. Four to five month months
4. All six months
5. Don't know (997)
6. Prefer not to say (999)

SEP_6: How often in the **<u>last 6 months</u>** would you say you were worried or stressed about being forced to move?

1. Never
2. Rarely
3. Sometimes
4. Often
5. Always
6. Don't know (997)
7. Prefer not to say (999)

SEP_7: I previously asked about sleeping outside in the last month. Now I'd like to ask, in the **last 6 months**, have you spent more than one night sleeping outside (like on the street, in a park, in a tent or encampment), in a vehicle, in an abandoned building, or in a transit station?

1. Yes
2. No
3. Don't know (997)
4. Prefer not to say (999)

If SEP_7 = No (2), skip to SEP_9;
Else ask

SEP_8: In the **last 6 months**, have you been voluntarily or involuntarily (forcibly) relocated (meaning you had to change your living location) due to an “encampment sweep” or “clean up” of public areas where you were living?

5. Yes
6. No
7. Don't know (997)
8. Prefer not to say (999)

If SEP_8a = No (2), or Prefer not to say (999) (3), skip to SEP_9;
Else ask

SEP_8a: Due to this voluntary or involuntary (forced) relocation, did you lose any of the following personal belongings?

1. Your ID card(s)
2. Any medication
3. Clothing
4. Phone
5. Other items (specify:)____________________
6. I did not lose any personal belongings as a result of the relocation
7. Prefer not to say (999)

If SEP_8a ! = Other items (5), then skip to SEP_8b;
Else ask

SEP_8a1: Other items: ______ *(write in)*

SEP_8b: Due to this voluntary or involuntary (forced) relocation, did you have more difficulty accessing health or social services or benefits?

1. Yes
2. No
3. Don't know (997)
4. Prefer not to say (999)

SEP_9: At any point in the **last 6 months**, have you had some form of government-issued personal identification (such as a driver’s license or other form of state-issued ID card)?

1. Yes
2. No
3. Don't know (997)
4. Prefer not to say (999)

If SEP_9 = No (2), Don't know (997) (3), or Prefer not to say (999) (4), skip to SEP_10;
Else ask

SEP_9a: Which of the following forms of government-issued identification have you had in the **last 6 months**? *(Select all that apply)*

1. State-issued driver's license
2. State-issued non-license identification card
3. Health insurance card (***MassHealth if MA, Medicaid Card if RI or VT)***
4. Passport
5. Other (please specify: ___________)

If SEP_9a! = Other (5), then skip to SEP_9b;
Else ask

SEP_9a1: Other: ______ *(write in)*

SEP_9b: In the **last 6 months**, have any of your forms of identification (e.g., government issued ID card) been lost or stolen?

1. Yes
2. No
3. Don't know (997)
4. Prefer not to say (999)

SEP_10: In the **last 6 months**, have you had difficulty accessing health or social services or benefits (e.g., medical appointments, unemployment benefits) because you could not show the required form of identification (e.g., government issued ID card)?

1. Yes
2. No
3. Don't know (997)
4. Prefer not to say (999)

SEP_11: In the **last 6 months**, did you receive any money from any of the following sources? *(Select all that apply)*

1. Full or part-time employment, on the books
2. Full or part-time employment, off the books
3. Temporary work or odd jobs
4. Recycling cans, returning bottles for deposits
5. Panhandling
6. Public assistance or disability
7. Parents
8. Friends or family members (not parents)
9. Husband/wife or domestic partner
10. Drug selling
11. Sex work, or sexual activities of any sort (like a hand job, blowjob, or sex)
12. Sex work as a pimp or manager
13. Other activities that are not legal
14. Something else:____________
15. I did not receive any money in the last six months
16. Don't know (997)
17. Prefer not to say (999)

If SEP_11 ! = Something else (14), skip to SEP_12;
Else ask

SEP_11a: Something else: ______________

SEP_12: What is your current employment status? *(Select all that apply)*

1. Working, part-time
2. Working, full-time
3. Not working, disabled
4. Not working, retired
5. Not working, looking for work

6. Not working, not looking for work
7. Not working, full- or part-time student
8. Not allowed to work
9. Something else: ___________
10. None of the above
11. Don't know (997)
12. Prefer not to say (999)

If SEP_12 != Something else (9), skip to SEP_13;
Else ask

SEP_12a: Something else: ______

SEP_13: Are you currently receiving any public cash benefits (such as workers' compensation, unemployment, TANF/SNAP, or SSI/SSD)?

1. Yes
2. No
3. Don't know (997)
4. Prefer not to say (999)

SEP_14: What is your average take home **monthly** income, including public or family support (such as cash from cash assistance, welfare, or TANF/SNAP)?
(Interviewer note: if needed, clarify that we mean post-tax income)

1. $0
2. < $1 - $500
3. $501 - $1,500
4. $1,501 - $3,000
5. > $3,000
6. Don't know (997)
7. Prefer not to say (999)

SEP_15: Do you currently have health insurance or healthcare coverage?

1. Yes
2. No
3. Don't know (997)
4. Prefer not to say (999)

If SEP_15 = No (2) or Don't know (997) (3) or Prefer not to say (999) (4), skip to SEP_16;
Else ask

SEP_15a: What kind of health insurance or healthcare coverage do you currently have? *(Select all that apply)*

1. Private (e.g., Blue Cross, Cigna, Aetna, United, Kaiser) *(Note: could be through self, family, employer)*
2. Medicaid (***MassHealth if MA, HealthSource RI if RI, Green Mountain Care if VT***)
3. Medicare/SSDI
4. VA/TRICARE
5. Something else: _____
6. None of the above
7. Don't know (997)
8. Prefer not to say (999)

If SEP_15a != Something else (5), skip to SEP_16;
Else ask

SEP_15b: Something else: ______

If SEP_15a = 2, skip to SEP_16a

SEP_16: During the **last six months**, were you ever covered by Medicaid, also known as ***[IF MA "MassHealth" or IF RI "HealthSourceRI" or IF VT "Green Mountain Care"]***?

1. Yes
2. No
3. Don't know (997)
4. Prefer not to say (999)

If SEP_16 != Yes (1), skip to SEP_17;
Else ask

SEP_16a: During the **last six months**, did you lose or have your Medicaid coverage cancelled?

1. Yes
2. No
3. Don't know (997)
4. Prefer not to say (999)

If SEP_16a != Yes (1), skip to SEP_17;
Else ask

SEP_16b: What was the main reason your Medicaid coverage ended?

1. I did not receive renewal papers
2. I received papers but did not understand them
3. I moved and did not receive notice
4. I did not meet the work requirements
5. My income increased
6. I started a new job
7. I missed the deadline to reapply
8. The eligibility changed
9. Other:_____________
10. Don't know (997)
11. Prefer not to say (999)

If SEP_16b ! = Other (8), skip to SEP_17;
Else ask

SEP_16b1: Other: ______

SEP_17: Do you have regular, consistent access to the Internet?

1. Yes
2. No
3. Don't know (997)
4. Prefer not to say (999)

SEP_18: Do you have regular, consistent access to a phone?
1. Yes
2. No
3. Don't know (997)
4. Prefer not to say (999)

SEP_19: In the **last six months**, how often have you seen or heard physical violence, such as fights, stabbing, or shootings in the neighborhood you spend the most time in?
1. Never
2. Rarely
3. Sometimes
4. Often
5. Don't know (997)
6. Prefer not to say (999)

SEP_20: In the l**ast six months**, have you been physically attacked, mugged, or threatened with a weapon in the neighborhood you spent the most time in?
1. Never
2. Once
3. A few times
4. Many times
5. Don't know (997)
6. Prefer not to say (999)

Substance Use Block

Drug & Alcohol Use

Prompt: This section is going to ask about drug use. We're going to start with a few questions about alcohol:

DRG_0: Have you ever had a drink containing alcohol?

1. Yes
2. No
3. Don't know (997)
4. Prefer not to say (999)

If DRG_0 = (2) No OR (3) Don't know (997) OR (4) Prefer not to say (999), skip to DRG_2;
Else ask

DRG_0a: How old were you the first time you used alcohol? *Write in #:* __________

The next questions focus on the last year (12 months).

DRG_1: How often did you have a drink containing alcohol in the **last year**?

1. Never
2. Monthly or less
3. Two to four times a month
4. Two to three times per week
5. Four or more times per week
6. Don't know (997)
7. Prefer not to say (999)

If DRG_1 = (1) Never OR (6) Don't know (997) OR (7) Prefer not to say (999), skip to DRG_2;
Else ask

DRG_1a: How many drinks containing alcohol did you have on a typical day when you were drinking in the **last year**?

1. 1-2 drinks
2. 3-4 drinks
3. 5-6 drinks
4. 7-9 drinks
5. 10 or more
6. Don't know (997)
7. Prefer not to say (999)

DRG_1b: How often did you have six or more drinks on one occasion in the **last year**?

1. Never
2. Less than monthly
3. Monthly
4. Weekly
5. Daily or almost daily
6. Don't know (997)

7. Prefer not to say (999)

DRG_1c: How often during the **last year** have you found that you were not able to stop drinking once you started?

1. Never
2. Less than monthly
3. Monthly
4. Weekly
5. Daily or almost daily
6. Don't know (997)
7. Prefer not to say (999)

DRG_1d: How often during the **last year** have you failed to do what was normally expected from you because of drinking?

1. Never
2. Less than monthly
3. Monthly
4. Weekly
5. Daily or almost daily
6. Don't know (997)
7. Prefer not to say (999)

DRG_1e: How often during the **last year** have you needed a first drink in the morning to get yourself going after a heavy drinking session?

1. Never
2. Less than monthly
3. Monthly
4. Weekly
5. Daily or almost daily
6. Don't know (997)
7. Prefer not to say (999)

DRG_1f: How often during the **last year** have you had a feeling of guilt or remorse after drinking?

1. Never
2. Less than monthly
3. Monthly
4. Weekly
5. Daily or almost daily
6. Don't know (997)
7. Prefer not to say (999)

DRG_1g: How often during the **last year** have you been unable to remember what happened the night before because you had been drinking?

1. Never
2. Less than monthly
3. Monthly
4. Weekly
5. Daily or almost daily
6. Don't know (997)
7. Prefer not to say (999)

DRG_1h: Have you or someone else been injured as a result of your drinking?
1. No
2. Yes, but not in the last year
3. Yes, during the last year
4. Don't know (997)
5. Prefer not to say (999)

DRG_1i: Has a relative or friend or a doctor or another health worker been concerned about your drinking or suggested you cut down?
1. No
2. Yes, but not in the last year
3. Yes, during the last year
4. Don't know (997)
5. Prefer not to say (999)

Optional Prompt: Okay, these questions will still be asking about alcohol, just during slightly different time periods.

DRG_1j: Did you use alcohol in the last **six months**?
1. Yes
2. No
3. Don't know (997)
4. Prefer not to say (999)

If DRG_1j = (2) No OR (3) Don't know (997) OR (4) Prefer not to say (999), skip to DRG_2
Else ask

DRG_1k: Did you use alcohol in the last **month**?
1. Yes
2. No
3. Don't know (997)
4. Prefer not to say (999)

If DRG_1k = (2) No OR (3) Don't know (997) OR (4) Prefer not to say (999), skip to DRG_2
Else ask

DRG_1l: How often did you use alcohol in the last **month**?
1. More than once a day
2. Every day
3. A few times a week
4. Once a week
5. A few times a month
6. Don't know (997)
7. Prefer not to say (999)

Prompt: In this next section, we are only talking about drugs that you meant to use — we'll be asking about unexpected cuts later. Note that the statement "not as prescribed" refers to any use without a prescription or not as doctor directed. This could mean buying it off the street, using it in a way that was not prescribed, like crushing and snorting, or using it in a dose that was not prescribed. Optional Prompt: I will ask you a series of questions about your use of each drug on the list. Some questions will be *ever* and some will be about *the last month only*.

Drug Use Table: Use for DRG_2

**DRG_2.**		1.	2.	3.	4.	5.	6.
		Have you ever used [insert drug]? (y/n)	If yes to ever, How old were you the first time you used [insert drug]	If yes to ever: Last 6 months (Y/N)	If yes to Last 6 Months: Last month (Y/N)	If yes to last month: How often did you use *X* drug in the last month:<br>1. More than once a day<br>2. Every day<br>3. A few times a week<br>4. Once a week<br>5. A few times a month<br>6. Don't know (997)<br>7. Prefer not to say (999)	If yes to last month, how did you use it, *(Select all that apply)*:<br>1. Orally *(Note: i.e., "ate/drank/swallowed, gummed")*<br>2. Injected<br>3. Sniffed<br>4. Smoked *(Note: this includes vaping)*<br>5. Booty bumped *(Note: i.e., "boofed")*<br>6. Don't know (997)<br>7. Prefer not to say (999)
DRG_2a	Powder cocaine						
DRG_2b	Crack cocaine						
DRG_2c	Methamphetamine (Meth/Tina/Crank)						
DRG_2d	Heroin, dope, or fentanyl (that you knew was fentanyl at time of use)						
DRG_2e	Prescription opioids (not as prescribed)						

DRG_2.		1.	2.	3.	4.	5.	6.
		Have you ever used [insert drug]? (y/n)	If yes to ever, How old were you the first time you used [insert drug]	If yes to ever: Last 6 months (Y/N)	If yes to Last 6 Months: Last month (Y/N)	If yes to last month: How often did you use *X* drug in the last month:<br>1. More than once a day<br>2. Every day<br>3. A few times a week<br>4. Once a week<br>5. A few times a month<br>6. Don't know (997)<br>7. Prefer not to say (999)	If yes to last month, how did you use it, *(Select all that apply)*:<br>1. Orally *(Note: i.e., "ate/drank/swallowed, gummed")*<br>2. Injected<br>3. Sniffed<br>4. Smoked *(Note: this includes vaping)*<br>5. Booty bumped *(Note: i.e., "boofed")*<br>6. Don't know (997)<br>7. Prefer not to say (999)
DRG_2f	Methadone (not as prescribed)						
DRG_2g	Buprenorphine/Suboxone (not as prescribed)						
DRG_2h	Xylazine/Tranq dope (that you knew was xylazine at time of use)						
DRG_2i	Benzos (Benzodiazepines) or other prescription sedatives (not as prescribed)						
DRG_2j	Prescription stimulants (not as prescribed)						
DRG_2k	LSD/Acid						
DRG_l	PCP						
DRG_2m	Ecstasy/MDMA						

**DRG_2.**		1.	2.	3.	4.	5.	6.
		Have you ever used [insert drug]? (y/n)	If yes to ever, How old were you the first time you used [insert drug]	If yes to ever: Last 6 months (Y/N)	If yes to Last 6 Months: Last month (Y/N)	If yes to last month: How often did you use *X* drug in the last month:<br>1. More than once a day<br>2. Every day<br>3. A few times a week<br>4. Once a week<br>5. A few times a month<br>6. Don't know (997)<br>7. Prefer not to say (999)	If yes to last month, how did you use it, *(Select all that apply)*:<br>1. Orally *(Note: i.e., "ate/drank/swallowed, gummed")*<br>2. Injected<br>3. Sniffed<br>4. Smoked *(Note: this includes vaping)*<br>5. Booty bumped *(Note: i.e., "boofed")*<br>6. Don't know (997)<br>7. Prefer not to say (999)
DRG_2n	Magic mushrooms (Psilocybin)						
DRG_2o	Ketamine						
DRG_2p	Kratom and/or Kratom extracts (7-OH, mitragynine, OPMS Gold)						

Prompt: A big goal of this study is to learn about new and different drugs people are using over time. Have you ever intentionally used anything else that I didn't ask about?

**DRG_3.**		1.	2.	3.	4.	5.	6.
		Ever (y/n)	If yes to ever, How old were you the first time you used [insert drug]	If yes to ever, Last 6 months (Y/N)	If yes to Last 6 Months, Last month (Y/N)	If yes to last month, How often did you use X drug in the last month:<br>1. More than once a day<br>2. Every day<br>3. A few times a week<br>4. Once a week<br>5. A few times a month<br>6. Don't know (997)<br>7. Prefer not to say (999)	If yes to last month, how did you use it,select all that apply:<br>1. Orally *(Note: i.e., "ate/drank/swallowed, gummed")*<br>2. Injected<br>3. Sniffed<br>4. Smoked *(Note: this includes vaping)*<br>5. Booty bumped *(Note: i.e., "boofed")*<br>6. Don't know (997)<br>7. Prefer not to say (999)
DRG_3a	Other *(write in)*						
DRG_3b							
DRG_3c							
DRG_3d							
DRG_3e							
DRG_3f							
DRG_3g							
DRG_3h							

Prompt: We are going to ask you about which drugs you use together. An important term to note: when we say "together" we mean in the same use session. So you might be using the drugs in different ways (for example, you smoke one, and inject another), but you use them together or back to back.

DRG_4: What drugs have you ever used together or back to back?

**Gate Logic/Prompt**	DRG_4.		1.	3.	4.
			If yes to ever, Last 6 months (Y/N)	If yes to last six months, How often did you use X drug in the last month:<br>1. More than once a day<br>2. Every day<br>3. A few times a week<br>4. Once a week<br>5. A few times a month<br>6. Don't know (997)<br>7. Prefer not to say (999)	If yes to last six months, how did you use it, select all that apply:<br>1. Orally *(Note: i.e., "ate/drank/swallowed, gummed")*<br>2. Injected<br>3. Sniffed<br>4. Smoked *(Note: this includes vaping)*<br>5. Booty bumped *(Note: i.e., "boofed")*<br>6. Don't know (997)<br>7. Prefer not to say (999)
IF DRG_2a1 = 1 AND DRG_2d1 = 1 then ask:	DRG_4b	Powder cocaine and fentanyl			
IF DRG_2b1 = 1 AND DRG_2d1 = 1 then ask:	DRG_4c	Crack cocaine and fentanyl			
IF DRG_2c1 = 1 AND DRG_2d1 = 1 then ask:	DRG_4a	Meth and fentanyl			
Prompt: are there any other combinations of drugs you used back to back?	DRG_4d	Other (write in):			
	DRG_4e				
	DRG_4f				
	DRG_4g				

Prompt: Now I am going to ask you about injection practices more generally.

INJ_1.	Have you ever injected drugs?<br><br>***If INJ_1 = No (2), Don't know (997) (3), or Prefer not to say (999) (4), skip to SMK_1;***<br>***Else Ask***	1. Yes<br>2. No<br>3. Don't know (997)<br>4. Prefer not to say (999)
INJ_2.	How old were you when you first injected drugs?	**Age (in years): ____**<br>997. Don't know (997)<br>999. Prefer not to say (999)
INJ_3.	Have you injected drugs in the last month?<br><br>***If INJ_3 = No (2), Don't know (997) (3), or Prefer not to say (999) (4), skip to SMK_1;***<br>***Else ask***	1. Yes<br>2. No<br>3. Don't know (997)<br>4. Prefer not to say (999)

(Interviewer note: If respondent answers with a range of numbers, prompt them to select a specific number)

HRD_1: Where have you gotten new syringes in the **last month**? *(Select all that apply)*

1. Syringe services program (SSP), otherwise known as a syringe exchange program (including mobile delivery of supplies from an SSP)
2. Friend, family, or sex partner who went to a SSP
3. Friend, family, or sex partner who did not go to a SSP
4. Someone who sells needles illegally
5. By mail from an online retailer (e.g., Amazon)
6. By mail from a harm reduction program that did not charge you for the syringes
7. Drugstore or pharmacy
8. Physician/doctor’s office
9. Substance use disorder treatment program
10. Retail store (e.g. veterinary or medical supply store)
11. Picked up off street or found it
12. Somewhere else: ____
13. Did not get new/unused syringes in the last month
14. Don't know (997)
15. Prefer not to say (999)

If HRD_1 ! = Somewhere else (12), then skip to INJ_4;
Else ask

HRD_1a: Somewhere else: ______ (write in)

INJ_4. In the **last month**, did you give or lend a syringe to someone else after you had used it?

1. Yes
2. No

3. Don't know (997)
4. Prefer not to say (999)

INJ_5. In the **last month**, did you receive and use a syringe from someone after they had used it?

1. Yes
2. No
3. Don't know (997)
4. Prefer not to say (999)

INJ_6. In the **last month**, how often have you used discarded syringes that you found (e.g., in the trash, on the ground)?

1. Often
2. Sometimes
3. Rarely
4. Never
5. Don't know (997)
6. Prefer not to say (999)

INJ_7. In the **last month**, did you share cookers, cottons, or rinse water with someone else?

1. Yes
2. No
3. Don't know (997)
4. Prefer not to say (999)

Optional Prompt: Now I am going to ask you about smoking drugs aside from cannabis and tobacco, which I will ask about later.

SMK_1.	Have you ever smoked any drugs aside from cannabis or tobacco products?<br><br>***If SMK_1 = No (2), Don't know (997) (3), or Prefer not to say (999) (4), skip to CMD_1;***<br>***Else ask***	1. Yes<br>2. No<br>3. Don't know (997)<br>4. Prefer not to say (999)
SMK_2.	How old were you when you first smoked any drugs? (Excluding cannabis/tobacco)	**Age (in years): ____**<br>997. Don't know (997)<br>999. Prefer not to say (999)
SMK_3.	Have you smoked drugs in the last month? (Excluding cannabis/tobacco.)<br><br>***If SMK_3 = No (2), Don't know (997) (3), or Prefer not to say (999) (4), skip to CMD_1; Else ask***	1. Yes<br>2. No<br>3. Don't know (997)<br>4. Prefer not to say (999)

Optional Prompt: These questions will all be asking you about the last month.

	1. Yes

**In the <u>last month</u> …**		2. No<br>3. Don't know (997)<br>4. Prefer not to say (999)
SMK_3a.	***If INJ_3 = No (2), Don't know (997) (3), or Prefer not to say (999) (4), skip to: SMK_3b;***<br>***Else ask***<br><br>…did you inject and smoke drugs in the same "session"? (Excluding cannabis/tobacco.)	
SMK_3b.	…did you use a broken pipe?	
SMK_3c	…did you use materials you found or create a makeshift device to smoke with?	
SMK_3d.	…did you smoke drugs using a pipe that <u>you knew</u> had been used by someone else?	
SMK_3e.	…did you smoke alone in a location where no one could see you if you overdosed?	
SMK_3f.	…did you smoke in a public place, like a park, alleyway, or sidewalk?	
SMK_3g.	…did you rush while smoking because you were concerned someone would see or interrupt you?	

HRD_2: Where have you gotten new/unused <u>pipes</u> in the **<u>last month</u>**? *(Select all that apply)*
1. Syringe services program (SSP), otherwise known as a syringe exchange program (including mobile delivery of supplies from an SSP)
2. Friend, family, or sex partner who went to a SSP
3. Friend, family, or sex partner who did not go to a SSP
4. Someone who sells pipes illegally
5. By mail from a harm reduction program that did not charge you for the pipes
6. By mail from an online retailer (e.g., Amazon)
7. Retail store (e.g., smoke shop)
8. Drugstore or pharmacy
9. Physician/doctor's office/clinic
10. Substance use disorder treatment program
11. Somewhere else: _______
12. Did not get new/unused pipes in the last month
13. Don't know (997)
14. Prefer not to say (999)

If HRD_2 ! = Somewhere else (11), skip to CMD_1;
Else ask

HRD_2a: Somewhere else: ______ (write in)

Prompt: Injecting or smoking drugs, especially drugs like "tranq" (ie, xylazine), may cause skin wounds. There are different types of skin wounds. Skin abscesses are lumps that may be red, warm, and infected. Ulcers are wounds that are open and may be infected. Cellulitis is an infection that is deeper in the skin or under the skin that spreads. The area where it spreads is usually red, warm and swollen.

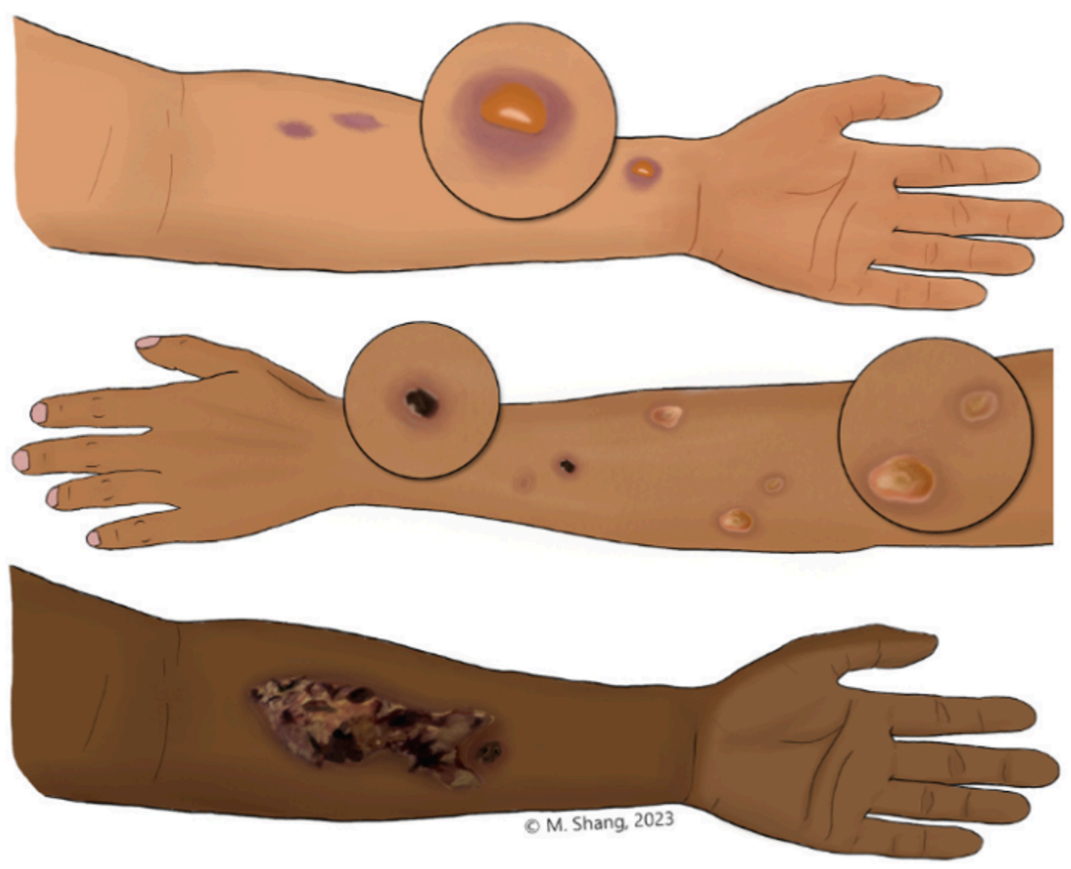<br><br>**Figure 1.**<br>*Xylazine-associated wound visual aid, reproduced from Jawa et al. (2024).*	**Abscesses**<br><br>**Ulcers**<br><br>**Cellulitis**

CMD_1a. Have you had any cellulitis in the **last 6 months**?
1. Yes
2. No
3. Don't know (997)
4. Prefer not to say (999)

CMD_1b Have you had any skin ulcers in the **last 6 months**?
1. Yes
2. No
3. Don't know (997)
4. Prefer not to say (999)

CMD_1c. Have you had any skin abscesses in the **last six months**?
1. Yes
2. No
3. Don't know (997)
4. Prefer not to say (999)

If CMD_1a = (1) Yes OR CMD_1b = (1) Yes OR CMD_1c = (1) Yes ask CMD_1d;
Else skip to CMD_2

CMD_1d. Were any of these wounds at an injection site?
1. Yes

2. No
3. Don't know (997)
4. Prefer not to say (999)

CMD_1e. **<u>In the last six months</u>**, have you or someone else who is <u>not</u> a healthcare provider treated any of your skin wounds?
1. Yes
2. No
3. Don't know (997)
4. Prefer not to say (999)

CMD_1f. **<u>In the last six months</u>**, did you *<u>seek</u>* treatment for any of your skin wounds from a healthcare provider?
1. Yes
2. No
3. Don't know (997)
4. Prefer not to say (999)

CMD_1g. **<u>In the last six months</u>**, did you *<u>receive</u>* treatment for any of your skin wounds from a healthcare provider?
1. Yes
2. No
3. Don't know (997)
4. Prefer not to say (999)

CMD_1j. **<u>In the last six months</u>**, has a skin wound changed the way you use drugs?
1. Yes
2. No
3. Don't know (997)
4. Prefer not to say (999)

If CMD_1j = Yes (1), skip to CMD_2
Else ask ask CMD_1k

CMD_1k. How did it change the way you use drugs? *(Select all that apply)*
1. I changed up how I use (e.g., sometimes smoking or sniffing instead of injecting every time)
2. I used mouthpieces on pipes when I smoke
3. I rotated the injection site
4. I injected into the wound
5. I tried to inject less often
6. Something else: __________
7. Don't know (997)
8. Prefer not to say (999)

If CMD_1k ! = Something else (6), then skip to CMD_2
Else ask

CMD_1i1: Something else: ______ *(write in)*

	1.	2.	3.	4.
CMD_2	**Have you ever been diagnosed with…? (select all that apply)**<br>1. Yes<br>2. No<br>3. Don't know (997)<br>4. Prefer not to say (999)	If CMD_2(1)=1: **Did this diagnosis occur in the last 6 months?**<br>1. Yes<br>2. No<br>3. Don't know (997)<br>4. Prefer not to say (999)	If CMD_2(1)=1: **Have you ever been hospitalized for ___?**<br>1. Yes<br>2. No<br>3. Don't know (997)<br>4. Prefer not to say (999)	If CMD_2(3)=1: **Did this hospitalization occur in the last 6 months?**<br>1. Yes<br>2. No<br>3. Don't know (997)<br>4. Prefer not to say (999)
CMD_2a. Blood infections				
CMD_2b. Endocarditis				
CMD_2c. Bone/joint infections				

DGG_1: In the **last month**, have you used drugs *(select all that apply)*:
1. Alone
2. With someone who is using drugs at the same time
3. With someone who is not using drugs at the same time
4. Don't know (997)
5. Prefer not to say (999)

DGG_2: In the **last month**, what are the places where you have used drugs? *(select all that apply)*
1. Public outdoor location (e.g., public park, alleyway)
2. Public indoor location (i.e., a public bathroom)
3. At a private indoor location (i.e., at a home or apartment)
4. In a tent
5. In a vehicle
6. At an overdose prevention center (i.e., a place where pre-obtained substances can be consumed under supervision)
7. Don't know (997)
8. Prefer not to say (999)

Prompt: We now want to ask about your seller/dealer. Please remember that all of your responses are strictly confidential:

DGG_3: Do you generally go to one primary seller/dealer?

1. Yes
2. No
3. Don't know (997)
4. Prefer not to say (999)

If DGG_3 = (1) Yes, I have a primary dealer, ask DGG_3a; else skip to UNX_1

DGG_3a: Do you trust your primary seller/dealer? For example, you trust their supply, the quality, and what they tell you is in the drugs?

1. Yes
2. Neutral
3. No
4. Don't know (997)
5. Prefer not to say (999)

Unexpected Drugs/Cuts

Prompt: Now, we'll talk about drugs you weren't necessarily meaning to use, like unexpected cuts that may have been in your supply.

UNX_1: In the last month, did you ever feel like there were unexpected drugs or cuts in your supply (like fentanyl or xylazine)?

1. Yes
2. No
3. Don't know (997)
4. Prefer not to say (999)

If UNX_1 = (1) Yes, ask UNX_2
Else skip to INJ_1

UNX_2: What were those drugs? *(Please select all that apply)*

1. Fentanyl
2. Xylazine
3. Medetomidine
4. BTMPS
5. Nitazines
6. Something else
7. Don't know (997)
8. Prefer not to say (999)

If UNX_2 = Something else (6), ask UNX_2a:

UNX_2a: Something else: ______ *(write in)*

If UNX_2 = (1) Fentanyl, ask UNX_3:

UNX_3: The <u>last time</u> you used drugs that unexpectedly contained fentanyl, what was the drug sold as or what did you think you were taking?

1. Powder cocaine
2. Crack cocaine
3. Heroin
4. Methamphetamine
5. Prescription opioid pain pills
6. Benzodiazepines
7. Prescription stimulants
8. Something else: ______
9. Don't know (997)
10. Prefer not to say (999)

If UNX_3 = Something else (8), ask UNX_3a:

UNX_3a: Something else: ______ *(write in)*

If UNX_2 = (1) Fentanyl, ask UNX_4:

UNX_4: What made you think it contained fentanyl? *(Please select all that apply)*
1. Differences in appearance, smell, or taste
2. I had unexpected side effects
3. Different high
4. I used test strips
5. I used a drug checking service
6. The seller told me
7. Someone else told me
8. Something else: ______
9. Don't know (997)
10. Prefer not to say (999)

If UNX_4 = Something else (8), ask UNX_4a:

UNX_4a: Something else: ______ *(write in)*

If UNX_2 = (2) Xylazine, ask UNX_5:

UNX_5: The <u>last time</u> you used drugs that unexpectedly contained Xylazine, what was the drug sold as, or what did you think you were taking?
1. Powder cocaine
2. Crack cocaine
3. Heroin
4. Fentanyl
5. Methamphetamine
6. Prescription opioid pain pills
7. Benzodiazepines
8. Prescription stimulants
9. Something else: ______
10. Don't know (997)
11. Prefer not to say (999)

If UNX_5 = Something else (9), ask UNX_5a:

UNX_5a: Something else: ______ *(write in)*

If UNX_2 = (2) Xylazine, ask UNX_6

UNX_6: What made you think it contained xylazine? *(Please select all that apply)*
1. Differences in appearance, smell, or taste
2. I had unexpected side effects (e.g., sedation, wounds)
3. Different high
4. I felt high but still was sick
5. I used test strips
6. I used a drug checking service
7. The seller told me

8. Someone else told me
9. Something else: ______
10. Don't know (997)
11. Prefer not to say (999)

If UNX_6 = Something else (9), ask UNX_6a:

UNX_6a: Something else: ______ *(write in)*

If UNX_6 = (2) I had unexpected side effects (e.g., sedation, wounds), ask UNX_7

UNX_7: What were those side effects? (*Please select all that apply)*

1. I blacked out or forgot what happened
2. I was very sedated or falling over
3. I got a severe skin wound or infection
4. Something else: ______
5. Don't know (997)
6. Prefer not to say (999)

If UNX_7 = Something else (4), ask UNX_7a:

UNX_7a: Something else: ______ *(write in)*

If UNX_2 = (3) Medetomidine, ask UNX_8:

UNX_8: The <u>last time</u> you used drugs that unexpectedly contained medetomidine, what was the drug sold as or what did you think you were taking?

1. Powder cocaine
2. Crack cocaine
3. Heroin
4. Fentanyl
5. Methamphetamine
6. Prescription opioid pain pills
7. Benzodiazepines
8. Prescription stimulants (not as prescribed)
9. Something else: ______
10. Don't know (997)
11. Prefer not to say (999)

If UNX_8 = Something else (9), ask UNX_8a:

UNX_8a: Something else: ______ *(write in)*

If UNX_2 = (3) Medetomidine, ask UNX_9:

UNX_9: What made you think it contained medetomidine? *(Please select all that apply)*

1. Differences in appearance, smell, or taste
2. I had unexpected side effects

3. Different high
4. I used test strips
5. I used a drug checking service
6. The seller told me
7. Someone else told me
8. Something else: ______
9. Don't know (997)
10. Prefer not to say (999)

If UNX_9 = Something else (8), ask UNX_9a:

UNX_9a: Something else: ______ *(write in)*

If UNX_9 = (2) I had unexpected side effects (e.g., sedation, wounds), ask UNX_10:

UNX_10: What were those side effects? *(Please select all that apply)*
1. I blacked out or forgot what happened
2. I was very sedated or falling over
3. I got a severe skin wound or infection
4. Something else: ______
5. Don't know (997)
6. Prefer not to say (999)

If UNX_10 = Something else (4), ask UNX_10a

UNX_10a: Something else: ______ *(write in)*

If UNX_2 = (6) Something else, ask UNX_11

UNX_11: The <u>last time</u> you used drugs that unexpectedly contained ***[PIPE IN UNX_2 = (6) open text response]***, what was the drug sold as or what did you think you were taking?
1. Powder cocaine
2. Crack cocaine
3. Heroin
4. Fentanyl
5. Methamphetamine
6. Prescription opioid pain pills
7. Benzodiazepines
8. Prescription stimulants (not as prescribed)
9. Something else: ______
10. Don't know (997)
11. Prefer not to say (999)

If UNX_11 = Something else (9), ask UNX_11a:

UNX_11a: Something else: ______ *(write in)*

UNX_12: What made you think it contained ***[PIPE IN UNX_2 = (6) open text response]***?
(Please select all that apply)

1. Differences in appearance, smell, or taste
2. I had unexpected side effects
3. Different high
4. I used test strips
5. I used a drug checking service
6. The seller told me
7. Someone else told me
8. Something else: ______
9. Don't know (997)
10. Prefer not to say (999)

If UNX_12 = Something else (8), ask UNX_12a:

UNX_12a: Something else: ______ *(write in)*

Substance Use Disorders

**Prompt: Now I'm going to ask you about some challenges you might have had related to your drug use. Remember that all your answers are strictly confidential and that you can refuse to answer a question at any time.**<br><br>During the **last 6 months**…					
		1.	2.	3.	4.
		**Yes**	**No**	**Don't know (997)**	**Prefer not to say (999)**
SUD_1.	Did you use larger amounts of drugs or use them for a longer time than you planned or intended?				
SUD_2.	Did you try to control or cut down on your drug use but were unable to do it?				
SUD_3.	Did you spend a lot of time getting drugs, using them, or recovering from their use?				
SUD_4.	Did you have a strong desire or urge to use drugs?				
SUD_5.	Did you get so high or sick from using drugs that it kept you from working, going to school, or caring for children?				
SUD_6.	Did you continue using drugs even when it led to social or interpersonal problems?				
SUD_7.	Did you spend less time at work, school, or with friends because of your drug use?				
SUD_8.	Did you use drugs that put you or others in physical danger?				
SUD_9.	Did you continue using drugs even when it was causing you physical or psychological problems?				
SUD_10a.	Did you need to increase the amount of a drug you were taking so that you could get the same effects as before?<br><br>***If SUD_10a = No (2) or Don't know (997) (3) or Prefer not to say (999) (4), skip to SUD_11a ; Else Ask***				
SUD_10b.	Did using the same amount of a drug lead to it having less of an effect as it did before?				
SUD_11a.	Did you get sick or have withdrawal symptoms when you quit or missed taking a				

	drug?<br><br>***If SUD_11a = No (2) or Don't know (997) (3) or Prefer not to say (999) (4), skip to SUD_12; Else Ask***				
SUD_11b.	Did you ever keep taking a drug to relieve or avoid getting sick or having withdrawal symptoms?				

SUD_12: Which drugs have caused you to have some of the problems you noted above? *(Please select all that apply, note interviewer does not need to read entire list but can probe)*

1. None
2. Alcohol
3. Stimulants – Crack Cocaine (rock)
4. Stimulants – Powder Cocaine (coke)
5. Stimulants – Methamphetamine (meth, tina, crank)
6. Stimulants – Amphetamines (speed)
7. Opioids – Heroin (dope) or Fentanyl
8. Natural Cannaboids – Marijuana (weed), hashish (hash) or cannabis
9. Synthetic Marijuana (K2/Spice)
10. Synthetic Cathinones (Bath Salts)
11. Club Drugs – MDMA/GHB/Rohypnol (Ecstasy)
12. Dissociative Drugs – Ketamine/PCP (Special K)
13. Hallucinogens – LSD/Mushrooms (acid)
14. Sedatives - Xylazine (‘tranq’)
15. Inhalants – Solvents (paint thinner)
16. Prescription Medications – Depressants
17. Prescription Medications – Stimulants
18. Prescription Medications – Opioid Pain Relievers
19. Other: _________
20. Don't know (997)
21. Prefer not to say (999)

If SUD_12 ! = Other (20), then skip to SUD_13;
Else ask

SUD_12a: Other: ______ *(write in)*

SUD_13: What drug has caused the most serious problems for you in the last **<u>6 months</u>**? Please select only one.

1. None
2. Alcohol
3. Stimulants – Crack Cocaine (rock)
4. Stimulants – Powder Cocaine (coke)
5. Stimulants – Methamphetamine (meth, tina, crank)

6. Stimulants – Amphetamines (speed)
7. Opioids – Heroin (dope) or Fentanyl
8. Natural Cannaboids – Marijuana (weed), hashish (hash) or cannabis
9. Synthetic Marijuana (K2/Spice)
10. Synthetic Cathinones (Bath Salts)
11. Club Drugs – MDMA/GHB/Rohypnol (Ecstasy)
12. Dissociative Drugs – Ketamine/PCP (Special K)
13. Hallucinogens – LSD/Mushrooms (acid)
14. Sedatives - Xylazine (‘tranq’)
15. Inhalants – Solvents (paint thinner)
16. Prescription Medications – Depressants
17. Prescription Medications – Stimulants
18. Prescription Medications – Opioid Pain Relievers
19. Other: _________
20. Don't know (997)
21. Prefer not to say (999)

If SUD_13 ! = Other (20), skip to DUS_1;
Else ask

SUD_13a: Other: ______ *(write in)*

Drug Use Stigma

Optional Prompt: In this next section, I'm going to read some statements about stigma in healthcare settings toward people who use drugs. Please think about your experiences over the past six months and tell me how much you agree or disagree. Some of these statements may make you uncomfortable; just tell me if you need a break.

In the last six months…		**[1] Strongly Disagree**	**[2] Disagree**	**[3] Neither Agree nor Disagree**	**[4] Agree**	**[5] Strongly Agree**	**[999] Prefer not to say (999)**
DUS_1.	I have been treated disrespectfully by healthcare workers because of my drug use	□ 1	□ 2	□ 3	□ 4	□ 5	
DUS_2.	I do not feel welcome in healthcare settings	□ 1	□ 2	□ 3	□ 4	□ 5	
DUS_3.	I have been observed more closely than other patients in healthcare settings because of my drug use	□ 1	□ 2	□ 3	□ 4	□ 5	
DUS_4.	I have been ignored by healthcare workers once they learn or suspect that I use drugs	□ 1	□ 2	□ 3	□ 4	□ 5	
DUS_5.	People who are known or suspected to use drugs are "red flagged" in the healthcare system	□ 1	□ 2	□ 3	□ 4	□ 5	
DUS_6.	I am treated like a criminal in healthcare settings because I use drugs	□ 1	□ 2	□ 3	□ 4	□ 5	
DUS_7.	Healthcare workers have thought that I'm pill shopping or trying to con them into giving me prescription medications to get high or sell	□ 1	□ 2	□ 3	□ 4	□ 5	
DUS_8.	Healthcare workers have given me poor care because I use drugs	□ 1	□ 2	□ 3	□ 4	□ 5	
DUS_9.	I have avoided getting healthcare because I expect I will be treated badly by healthcare workers	□ 1	□ 2	□ 3	□ 4	□ 5	

Overdose

Prompt: For these questions, when I say 'opioid overdose', I mean a time when your breathing slowed down or you stopped breathing completely and someone had to 'breathe for you', or you lost consciousness and someone had to do something to bring you back like administer naloxone (i.e., Narcan). We will ask about stimulant overdoses later.

OVD_1: In the **last six months**, how many times did you overdose on drugs involving heroin, fentanyl or other opioids?

______ *(write in #)*
997. Don't know (997)
999. Prefer not to say (999)

If OVD_1 = Zero (0), skip to OVD_2;
Else Ask

OVD_1a: How many times in the **last month**? *[did you overdose on drugs involving heroin, fentanyl, or other opioids?]*

______ *(write in #)* ***Validate so OVD_1a ≤ OVD_1.***
997. Don't know (997)
999. Prefer not to say (999)

OVD_1b: What substances had you intentionally used during your most recent opioid overdose? *(Select all that apply)*

1. Powdered cocaine
2. Crack cocaine
3. Fentanyl
4. Xylazine
5. Methamphetamine
6. Prescription opioids (Oxycontin, Percocet)
7. Benzodiazepines ("Benzos", Xanax, Ativan)
8. Methadone
9. Buprenorphine
10. Cannabis
11. Other: ____________
12. Don't know (997)
13. Prefer not to say (999)

If OVD_1b ! = Other (12), then skip to OVD_1c;
Else ask

OVD_1b1: Other: ______ *(write in)*

OVD_1c: Did someone use naloxone (Narcan™) on you after your most recent opioid overdose involving heroin, fentanyl, or other opioids?

1. Yes
2. No
3. Don't know (997) or don't remember

4. Prefer not to say (999)

OVD_1d: Did you experience withdrawal symptoms because naloxone (Narcan™) was administered after your most recent overdose?

1. Yes
2. No
3. Don't know (997)
4. Prefer not to say (999)

OVD_1e: Do you think other non-opioid drugs (e.g., xylazine, medetomidine) were involved in your most recent overdose?

1. Yes
2. No
3. Don't know (997)
4. Prefer not to say (999)

OVD_1f: Were you transported to the emergency department by EMS after your most recent opioid overdose?

1. Yes
2. No, because I declined to go
3. No, because no one called EMS
4. Don't know (997)
5. Prefer not to say (999)

If OVD_1f = No, because I declined to go (2), ask OVD_1g

OVD_1g: Why did you decline going with EMS? (*Select all that apply*)

1. I did not think I needed to go
2. My withdrawal symptoms were too severe
3. I did not want to experience withdrawal symptoms in ED
4. I did not think that care in the ED would be helpful
5. The EMS personnel treated me badly
6. Fear of law enforcement involvement
7. Something else
8. Don't know (997)
9. Prefer not to say (999)

If OVD_1g ! = Something else (7), skip to HRD_3;
Else ask

OVD_1g1: Something else: ______ *(write in)*

Prompt: Now, we'll talk about stimulant overdoses or "overamping". When we say "overamp", we mean a time after using stimulants (like cocaine or meth) when you had severe and unexpected physical symptoms (like chest pain, nausea or vomiting, extreme sweating or uncontrolled high temperature, convulsions, seizures, cardiac arrest, or stroke) or mental health symptoms (like extreme anxiety, paranoia, fear, or feeling stuck or frozen). These severe symptoms generally last for an hour or longer.

OVD_2: In the **last six months**, how many times did you experience an overamp from using cocaine, methamphetamine, or other stimulants?

______ *(write in #).*
997. Don't know (997)
999. Prefer not to say (999)

If OVD_2 = Zero (0), skip to HRD_3
Else Ask

OVD_2a: How many times in the **last month** did you experience an overamp?

______ *(write in #)* ***Validate so OVD_2a ≤ OVD_2.***
997. Don't know (997)
999. Prefer not to say (999)

OVD_2b: What substances had you intentionally used during your most recent overamp? *(Select all that apply)*

14. Powdered cocaine
15. Crack cocaine
16. Fentanyl
17. Xylazine
18. Methamphetamine
19. Prescription opioids (Oxycontin, Percocet)
20. Benzodiazepines ("Benzos", Xanax, Ativan)
21. Methadone
22. Buprenorphine
23. Cannabis
24. Other: ____________
25. Don't know (997)
26. Prefer not to say (999)

If OVD_2b ! = Other (12), then skip to OVD_2c;
Else ask

OVD_2b1: Other: ______ *(write in)*

OVD_2c: What symptoms did you experience <u>during</u> your <u>most recent</u> overamp? *(Select all that apply)*

1. Difficulty breathing or not breathing
2. Loss of consciousness (blacked out)
3. Slowed heart rate
4. Strong desire to sleep
5. Unable to motivate yourself to move/get up
6. Anxiety
7. Hallucinations (e.g., seeing or hearing someone/something that is not there)
8. Heart attack
9. Heart pounding (intense feeling) and/or rapid heart rate (heart racing)
10. Hyperventilation
11. Paranoia
12. Seizure
13. Stroke

14. I don’t remember
15. Something else: _______
16. None of the above
17. Don't know (997)
18. Prefer not to say (999)

If OVD_2c ! = Something else (14), then skip to OVD_2d;
Else ask

OVD_2c1: Something else: ______ *(write in)*

OVD_2d: The last time you had an overamp, how long did the symptoms last?
1. Less than an hour
2. 1-2 hours
3. 3-5 hours
4. More than 5 hours
5. Don't know (997)
6. Prefer not to say (999)

OVD_2e: If someone had to help you after your most recent overamp, what did they do? (*Select all that apply*)
1. Don’t remember or Don't know (997)
2. No one helped me
3. Walked me around, slapped or otherwise physically stimulated (for example, Sternum Rub)
4. Monitored oxygen with a pulse oximeter (goes on a finger)
5. Gave rescue breathing
6. Oxygen
7. Hydration
8. Cold water or ice
9. Chest compressions
10. Gave naloxone (Narcan)
11. Gave me something other than naloxone (Narcan)
12. Someone beside EMS (a friend or bystander) took me to the ED
13. Took me to a drop in center
14. Called 911/EMS
15. Something else: _______
16. Prefer not to say (999)

If OVD_2e ! = Something else (14), then skip to OVD_2f;
Else ask

OVD_2e1: Something else: ______ *(write in)*

OVD_2f: Did you experience any <u>withdrawal</u> symptoms because of the intervention(s) administered after your most recent overamp?
1. Yes
2. No
3. Don't know (997)
4. Prefer not to say (999)

OVD_2g: Were you transported to the emergency department by EMS after your most recent overamp? *(Select all that apply)*

1. Yes
2. No, because I declined to go
3. No, because EMS was not called
4. Don't know (997)
5. Prefer not to say (999)

If OVD_2g = No, because I declined to go (2), ask OVD_2h

OVD_2h: Why did you choose not to go with EMS? (*Select all that apply*)

1. I did not need to go
2. Withdrawal symptoms were too severe
3. Did not want to experience withdrawal symptoms in ED
4. Expected that care in ED would not be helpful
5. The EMS personnel treated me badly
6. Fear of law enforcement involvement
7. Something else
8. Don't know (997)
9. Prefer not to say (999)

If OVD_2h ! = Something else (7), then skip to HRD_3;
Else ask

OVD_2h1: Something else: ______ *(write in)*

Harm Reduction

Optional Prompt: Next we'll talk about harm reduction and some of the services that you might have been using during the last 6 months.

HRD_3: In the **last 6 months**, how often did you get any supplies or services from a harm reduction program for yourself (not for others)?

1. Daily (or whenever the program I use is open)
2. More than once per week
3. Once per week
4. 1-3 times per month
5. I have not used services from a harm reduction program in the past six months
6. Don't know (997)
7. Prefer not to say (999)

If HRD_3 = (5) I have not used services from a harm reduction program in the past six months, (6) Don't know (997), or (7) Prefer not to say (999), skip to HRD_4;
Else ask

HRD_3a: In the **last 6 months**, where did you get supplies or services from a syringe exchange or other harm reduction program? *(Select all that apply)*

1. Brick and mortar location(s)
2. Mobile outreach (e.g., a van)
3. Vending machines
4. Mail order/delivery (e.g., ordered online and received at a mailing address)
5. Other, please specify:: ________
6. Prefer not to say (999)

If HRD_3a ! = Other (5), then skip to HRD_3b;
Else ask

HRD_3a1: Other: ______ *(write in)*

HRD_3b: In the **last 6 months**, what supplies did you get from a syringe exchange or other harm reduction program? *(Select all that apply)*

Injection Supplies
1. Syringes
2. Cookers and other works (cotton, cookers, ties/tourniquets, syringe/ pill filters, saline/ sterile water, skin prep pads)
3. Sharp container(s)

Smoking & Inhalation Supplies
4. Pipes
5. Other smoking supplies (brass screens/ brillo/ Choreboy, mouthpieces, straws)
6. Safer snorting supplies (e.g., straws)

Supplies for Other Routes of Administration
7. Booty bumping, boofing, or rectal administration supplies

Drug Safety Supply
8. Fentanyl test strips
9. Xylazine test strips

10. Naloxone (Narcan)

Testing & Sexual Health

11. HIV self-test kits or HIV “home tests”
12. Pregnancy tests
13. Safe sex supplies (e.g., condoms, lube)

First Aid & Medical

14. First aid or wound care supplies (e.g, bandages)

Personal Care & Hygiene

15. Personal hygiene kits
16. Feminine hygiene products
17. Liquid IV or oral rehydration packets

Basic Needs & Essential Items

18. Sleeping bags or blankets
19. Clothing items (clothes, socks, hats, gloves)
20. Backpacks & tote bags
21. Wagon/ carts

Other

22. Other, please specify: ____________________
23. Prefer not to say (999)

If HRD_3b ! = Other (22), then skip to HRD_3c;
Else ask

HRD_3b1: Other: ______ *(write in)*

HRD_3c: In the **<u>last 6 months</u>**, what <u>services</u> did you receive <u>directly</u> from a syringe exchange or other harm reduction program, meaning services you got there, on the spot, <u>not</u> referrals to other locations)? *(Select all that apply)*

HIV Prevention & Treatment

1. Pre-exposure prophylaxis (PrEP) for HIV prevention
2. Post-exposure prophylaxis (PEP) for HIV prevention following an exposure
3. HIV treatment/care (e.g., antiretroviral therapy)

Hepatitis C

4. Hepatitis C virus (HCV) testing
5. Hepatitis C virus (HCV) treatment/care

STIs & Other Infectious Diseases

6. Sexually transmitted infection (STI) testing
7. Sexually transmitted infection (STI) treatment/care
8. Other infectious disease testing
9. Vaccination (e.g., for influenza, Hepatitis A or B)

Clinical & Primary Care

10. Basic clinical services (e.g., wound care, primary care, physicals);
11. Mental health services (e.g., counseling, cognitive behavioral therapy)
12. Substance use disorder treatment services (e.g., medications for opioid use disorder like buprenorphine/Suboxone)
13. Acupuncture or holistic health services

Case Management & Navigation

14. Clinical case management or navigation services (e.g., PrEP navigation)
15. Non-clinical case management (e.g., housing coordination services)

Drug Checking

16. Drug checking services using a machine (a machine with results provided by a staff member or other professional)
17. Drug checking via mailing samples to an external lab (with results provided later)

Peer & Community Support

18. Peer support groups or peer counseling
19. Respite/drop-in center services (e.g., food, laundry, toilet, shower)
20. Storage for medications or other personal items

Other

21. Other, please specify: ______
22. Don't know (997)
23. Prefer not to say (999)

If HRD_3c ! = Other (21), then skip to HRD_3d;
Else ask

HRD_3c1: Other: ______ *(write in)*

HRD_3d: In the **last 6 months**, did someone at a syringe exchange or other harm reduction program refer you to any offsite services provided by another organization?

1. Yes
2. No
3. Don't know (997)
4. Prefer not to say (999)

If HRD_3d = (2) No, (3) Don't know (997), or (4) Prefer not to say (999), skip to HRD_4
Else ask

HRD_3e: What services did you get referred out to *(Select all that apply)*:

1. Pre-exposure prophylaxis (PrEP) for HIV prevention
2. Post-exposure prophylaxis (PEP) for HIV prevention following an exposure
3. HIV treatment/care (e.g., antiretroviral therapy)
4. Hepatitis C virus (HCV) testing
5. Hepatitis C virus (HCV) treatment/care
6. Sexually transmitted infection (STI) testing
7. Sexually transmitted infection (STI) treatment/care
8. Basic clinical services (e.g., wound care, primary care, physicals);
9. Mental health services (e.g., counseling, cognitive behavioral therapy)
10. Substance use disorder treatment services, whether inpatient or outpatient (e.g., medications for opioid use disorder like buprenorphine/Suboxone)
11. Other, please specify: __________
12. Don't know (997)
13. Prefer not to say (999)

If HRD_3e ! = Other (6), then skip to HRD_3f;
Else ask

HRD_3e1: Other: ______ *(write in)*

HRD_3f: In the **last 6 months**, how often did you get any supplies or services from a harm reduction program for others?

1. Daily (or whenever the program I use is open)
2. More than once per week
3. Once per week
4. 1-3 times per month
5. I have not obtained any supplies from a harm reduction program for other people in the last six months
6. Don't know (997)
7. Prefer not to say (999)

If HRD_3f = (5) I have not obtained any supplies from a harm reduction program for other people in the last six months (6) Don't know (997), or (7) Prefer not to say (999), skip to HRD_4;
Else ask

HRD_3g: In the **last 6 months**, what supplies did you get from a syringe exchange or other harm reduction program for other people? *(Select all that apply)*

Injection Supplies
1. Syringes
2. Cookers and other works (cotton, cookers, ties/tourniquets, syringe/ pill filters, saline/ sterile water, skin prep pads)
3. Sharp container(s)

Smoking & Inhalation Supplies
4. Pipes
5. Other smoking supplies (brass screens/ brillo/ Choreboy, mouthpieces, straws)
6. Safer snorting supplies (e.g., straws)

Other Routes of Administration
7. Booty bumping, boofing, or rectal administration supplies

Drug Supply Safety
8. Fentanyl test strips
9. Xylazine test strips
10. Naloxone (Narcan)

Testing & Sexual Health
11. HIV self-test kits or HIV “home tests”
12. Pregnancy tests
13. Safe sex supplies (e.g., condoms, lube)

First Aid & Medical
14. First aid or wound care supplies (e.g, bandages)

Personal Care & Hygiene
15. Personal hygiene kits
16. Feminine hygiene products
17. Liquid IV or oral rehydration packets

Basic Needs & Essential Items
18. Sleeping bags or blankets
19. Clothing items (clothes, socks, hats, gloves)
20. Backpacks & tote bags
21. Wagon/ carts

Other
22. Other, please specify: ___________________
23. Prefer not to say (999)

If HRD_3g ! = Other (22), then skip to HRD_4;
Else ask

HRD_3g1: Other: ______ *(write in)*

HRD_4: Do you currently have naloxone (Narcan) with you (for example, in your bag or a pocket)?
1. Yes
2. No
3. Don't know (997)
4. Prefer not to say (999)

HRD_5: Have you ever been taught or trained in how to use naloxone (Narcan)?
1. Yes
2. No
3. Don't know (997)
4. Prefer not to say (999)

HRD_6: Have you ever administered naloxone (Narcan) in response to an overdose?
1. Yes
2. No
3. Don't know (997)
4. Prefer not to say (999)

If HRD_6 = No (2), Don't know (997) (3), or Prefer not to say (999) (4), skip to HRD_7;
Else ask

HRD_6a: How many times in the **last six months** did you administer naloxone (Narcan) in response to an overdose?

______ times *(write in #)*
997. Don't know (997)
999. Prefer not to say (999)

HRD_7: In the **last 6 months**, where have you gotten naloxone (Narcan)? *(Select all that apply)*
1. Harm reduction program
2. Other community program
3. Vending machine
4. Prescription from doctor’s office or clinic
5. Drug treatment program
6. Pharmacy
7. Health department
8. On the street
9. Family member or a friend
10. Emergency responders (e.g., EMS, ambulance, police, fire)
11. Hospital
12. Emergency room
13. Work
14. Research study

15. Other, please specify: ________________________
16. I have not gotten naloxone (Narcan) in the last 6 months?
17. Don't know (997)
18. Prefer not to answer

If HRD_7 ! = Other (15), then skip to HRD_8;
Else ask

HRD_7a: Other: ______ *(write in)*

HRD_8: What do you usually do to avoid an overdose? *(Select all that apply)*
1. Nothing
2. Avoid mixing drugs with alcohol
3. Avoid mixing drugs with certain other drugs
4. Avoid mixing drugs at all
5. Avoid specific drugs
6. Not use alone
7. Use with someone else
8. Use at an overdose prevention center
9. Take smaller amounts
10. Go slow
11. Take a test shot or tester
12. Smoke or sniff instead of inject
13. Use test strips
14. Use a hotline or app that would notify emergency services if I became non-responsive
15. Keep naloxone (Narcan) on hand
16. Use a drug checking service, like a machine read by a professional
17. Going to a trusted dealer
18. Something else: __________

If HRD_8 ! = Something else (18), then skip to HRD_9;
Else ask

HRD_8a: Something else: ______ *(write in)*

Prompt: An important term for these next questions: an overdose prevention center is a place where people come to use their own drugs under the supervision of medically trained workers or peers, and they also have access to sterile supplies. Sometimes these are called supervised consumption sites.

HRD_9: Have you ever heard of an overdose prevention center before today?
1. Yes
2. No
3. Don't know (997)
4. Prefer not to say (999)

HRD_10: Have you <u>ever</u> used drugs at an overdose prevention center?
1. Yes

2. No
3. Don't know (997)
4. Prefer not to say (999)

If HRD_10 = (2) No, (3) Don't know (997), or (4) Prefer not to say (999), skip to HRD_13; Else ask

HRD_11: Have you used drugs at an overdose prevention center in the **last six months**?
1. Yes
2. No
3. Don't know (997)
4. Prefer not to say (999)

If HRD_11 = No (2), Don't know (997) (3), or Prefer not to say (999) (4), skip to HRD_13; Else ask

HRD_12: Thinking about your last 30-day drug use in general, how often did you use an overdose prevention center?
1. Every time
2. Most of the time
3. About half of the time
4. Some or a little of the time
5. Never
6. Don't know (997)
7. Prefer not to say (999)

Prompt: Now I'd like to ask you some questions about drug testing/checking

HRD_13: Have you **ever used** any of the following drug checking tools or services, which can be used to test the safety of your drug supply?? *(Select all the apply)*
1. Test strips (e.g., for fentanyl)
2. On-site drug checking machine read by a professional
3. Mail-in drug checking services
4. Something else: ______
5. None; I have never used any of these drug checking tools
6. Don't know (997)
7. Prefer not to say (999)

If HRD_13 = Something else (4), ask HRD_13a;
If HRD_13 = None; I have never used any of these drug checking tools (5), ask HRD_14 Else skip to HRD_15

HRD_13a: Something else: ______ *(write in)*

HRD_14: Why have you not used any drug checking tools or services? *(Select all the apply)*
1. Unaware; didn't know about them
2. Services were unavailable or difficult to access (locations, times)
3. Not interested in knowing whats in my drugs
4. No point in getting my drugs checked (I have no other alternatives)
5. Worried police will stop or arrest me

6. Worried other people will know that I use drugs
7. I want to use fentanyl
8. I want to use xylazine
9. I have my own way of checking my drugs
10. I worry it will use up too much of my drugs
11. I trust my source / supply of drugs
12. Other reason, please specify: _______

If HRD_14 = Other reason, please specify (12), ask HRD_14a
Else skip to SUT_1

HRD_14a: Other: ______ *(write in)*

HRD_15: Thinking about <u>the last time</u> you had your drugs checked, did the results show any unexpected or unwanted drugs or cuts in your supply?

1. Yes (there were unexpected or unwanted drugs or cuts)
2. No (nothing unexpected or unwanted)
3. The results were unclear
4. Don't know (997)
5. Prefer not to say (999)

HRD_16: What did you do because of these results? (*Select all that apply*)

1. Nothing, I used my drugs as usual
2. I used my drugs in an overdose prevention center ***[@HIDECHOICE if HRD_10 ! = 1]***
3. I reduced the dose I used
4. I went slower
5. I did a tester shot
6. I used with someone else around
7. I had naloxone (Narcan) on hand
8. I did not use because of the results
9. I gave the drugs away
10. I sold the drugs
11. I threw the drugs away
12. I told the person I got the drugs from what was in the batch
13. I told other people I know what was in the batch
14. Other, please specify: _______
15. Don't know (997)
16. Prefer not to say (999)

If HRD_16 ! = Other (14), then skip to SUT_1;
Else ask

HRD_16a: Other: ______ *(write in)*

SUD Treatment

Optional Prompt: Great — now we're going to ask about substance use treatment.

SUT_1:		1.	2.
		Has any doctor, nurse, counselor, or other healthcare professional ever told you that you have a substance use disorder related to *X*:<br>1. Yes<br>2. No<br>3. Don't know (997)<br>4. Prefer not to say (999)	How old were you the first time you were told you had *X* disorder? If you do not remember exactly, please give your best estimate.<br>____ *(write in #)*<br>997. Don't know (997)<br>999. Prefer not to say (999)
**SUT_1a.**	Alcohol use or alcohol use disorder (AUD)		
**SUT_1b.**	Opioid use or opioid use disorder		
**SUT_1c.**	Stimulant use (cocaine, methamphetamine) or stimulant use disorder		
**SUT_1d.**	Another drug use disorder (please specify: ______)		

Prompt: The next questions relate to any experiences you have had with treatment for *alcohol use or alcohol use disorder*.

If SUT_1a1 != (1) Yes, skip to SUT_3;
Else ask

SUT_2: In your lifetime, have you ever received any treatment for alcohol use or alcohol use disorder?
1. Yes
2. No
3. Don't know (997)
4. Prefer not to say (999)

If SUT_2 ! = (1) Yes, skip to SUT_3;
Else ask

SUT_2a: What type or types of alcohol treatment have you ever received? *(Select all that apply)*
1. Support group, group counseling or self-help group
2. AA
3. One-on-one counselling
4. Treatment program with a provider conducted through telemedicine

5. Day program
6. Residential treatment facility – stayed overnight
7. Detox program- stayed overnight
8. Naltrexone
9. Disulfram (Antabuse)
10. Acamprostate (Campral)
11. Other, please specify: ____________

If SUT_2a ! = Other (11), then skip to SUT_2b;
Else ask

SUT_2a1: Other: ______ *(write in)*

SUT_2b: In the **__last six months__**, have you received any treatment for alcohol use or alcohol use disorder?
1. Yes
2. No
3. Don't know (997)
4. Prefer not to say (999)

If SUT_2b ! = (1) Yes, skip to SUT_3
Else ask

SUT_2c: What type or types of alcohol treatment have you received in the **__last 6 month__**s? *(Select all that apply)*
1. Support group, group counseling or self-help group
2. AA
3. One-on-one counselling
4. Treatment program with a provider conducted through telemedicine
5. Day program
6. Residential treatment facility – stayed overnight
7. Detox program- stayed overnight
8. Naltrexone
9. Disulfram (Antabuse)
10. Acamprostate (Campral)
11. Other, please specify: ____________

If SUT_2c ! = Other (11), then skip to SUT_3;
Else ask

SUT_2c1: Other: ______ *(write in)*

Prompt: The next section asks about substance use disorder treatment for <u>drug use</u> only. This does not include treatment for <u>alcohol</u>.

**SUT_3:**		**1.**	**2.**	**3.**
		Have you ever received *X* treatment?<br>1. Yes<br>2. No	[If SUT_3(1)=1] Have you received *X* treatment in the last 6 months?<br>1. Yes<br>2. No	[If SUT_3(2)=1] Are you currently receiving X treatment?<br>1. Yes<br>2. No
Medications for Opioid Use Disorder (MOUD)				
SUT_3a.	Methadone			
SUT_3b.	Buprenorphine (Suboxone, Sublocade)			
SUT_3c.	Naltrexone (Vivitrol; extended-release naltrexone [XR-NTX] for opioid use disorder, not alcohol use disorder)			
Outpatient Treatment				
SUT_3d.	Detox (detoxification)			
SUT_3e.	Outpatient therapy or group sessions			
SUT_3f.	Partial hospitalization (intensive outpatient)			
Inpatient or Residential Treatment				
SUT_3g.	Hospitalization (inpatient treatment)			
SUT_3h.	Residential drug treatment program (non-hospital)			
SUT_3i.	Psychiatric inpatient treatment			
SUT_3j.	Recovery/sober living housing			
Other or Emerging Treatments				
SUT_3k.	Mutual support group (like AA or NA)			

SUT_3:		1.	2.	3.
		Have you ever received *X* treatment?<br>1. Yes<br>2. No	[If SUT_3(1)=1] Have you received *X* treatment in the last 6 months?<br>1. Yes<br>2. No	[If SUT_3(2)=1] Are you currently receiving X treatment?<br>1. Yes<br>2. No
SUT_3l.	Contingency management (i.e., getting payments or other incentives for not using drugs)			
SUT_3m.	Psychedelic therapy for substance use disorder (e.g., ketamine, psilocybin)			
SUT_3n.	GLP-1 medications for substance use disorder (e.g., Ozempic, Wegovy, Semaglutide)			
SUT_3o.	Another type of treatment for substance use disorder (*if yes, please specify: ____________*)<br><br>SUT_3o1a: Other: _______ *(write in)*			

Prompt: The next questions will be about the <u>last 6 months</u> only.

If SUT_3a(2) = (1) ask:

SUT_4: How long have you been or were you on methadone the most recent time?

1. Less than one month
2. One to three months
3. Three to six months
4. Longer than six months
5. Don't know (997)
6. Prefer not to say (999)

SUT_4a: How did you find your current or most recent methadone provider? *(Select all that apply)*

1. Visited clinic (walk-in)
2. Made phone calls
3. Asked friends
4. Referred by a doctor, nurse, counselor, or other healthcare professional
5. Offered or referred to by an emergency department or hospital
6. Offered or referred to by an SSP or other community organization
7. Offered or referred to by community outreach worker
8. Offered or referred to by police
9. Other, please specify: ________
10. Don't know (997)
11. Prefer not to say (999)

If SUT_4a ! = Other (9), then skip to SUT_4b;
Else ask

SUT_4a1: Other: ______ *(write in)*

SUT_4b: How long did it take you to get enrolled in your most recent methadone program?

1. Hours
2. Days
3. Months
4. Over a year
5. Don't know (997)
6. Prefer not to say (999)

SUT_4c: Why did you enroll in a methadone program during the **<u>last 6 months</u>**? *(Select all that apply)*

1. To transition away from street-based opioids
2. To alleviate symptoms of withdrawal
3. To top up when there is not enough money for drug of choice
4. For pain management
5. To ensure consistency in my supply
6. To reduce drug use for health reasons/concerns
7. To sleep/come down
8. For compliance with court-order or legal mandate
9. Another reason, please specify: _______

10. Don't know (997)
11. Prefer not to say (999)

If SUT_4c ! = Another reason (9), then skip to SUT_4d;
Else ask

SUT_4c1: Another reason: ______ *(write in)*

SUT_4d: How much do you agree that, during the **<u>last 6 months</u>**, your methadone providers have respected you and your values, beliefs, and feelings?
1. Strongly agree
2. Agree
3. Neutral
4. Disaggree
5. Strongly disagree
6. Don't know (997)
7. Prefer not to say (999)

SUT_4e: Overall, how satisfied have you been with your methadone providers during the **<u>last 6 months</u>**?
1. Extremely satisfied
2. Very satisfied
3. Satisfied
4. Somewhat satisfied
5. Dissatisfied
6. Extremely dissatisfied
7. Don't know (997)
8. Prefer not to say (999)

If SUT_3a(2)= 1 and SUT_3a(3)= 2, ask SUT_4f

SUT_4f: Why did you stop receiving methadone treatment? *(Select all that apply)*
1. Couldn't afford treatment
2. Not covered by health insurance
3. Completed planned treatment timeframe / duration
4. Transportation issues (e.g., too far away, no public transit available, transit is too expensive)
5. Difficulty scheduling appointments
6. Pregnant
7. Got incarcerated
8. Change my mind / did not want to continue in treatment
9. Discontinued by a treatment program / provider
10. Treatment wasn’t working / helping
11. Didn’t want to be seen getting treatment
12. Other, please specify:_____________

If SUT_4f = Other (12), then ask SUT_4f1

SUT_4f1: Other: ______ *(write in)*

If SUT_3b(2) = (1) ask:

SUT_5: How long have you been or were you on buprenorphine the most recent time?
1. Less than one month
2. One to three months
3. Three to six months
4. Longer than six months
5. Don't know (997)
6. Prefer not to say (999)

SUT_5a: How did you find your current or most recent buprenorphine prescriber? *(Select all that apply)*
1. Visited clinic (walk-in)
2. Made phone calls
3. Asked friends
4. Referred by a doctor, nurse, counselor, or other healthcare professional
5. Offered or referred to by an emergency department or hospital
6. Offered or referred to by an SSP or other community organization
7. Offered or referred to by community outreach worker
8. Offered or referred to by police
9. Other, please specify: ________
10. Don't know (997)
11. Prefer not to say (999)

If SUT_5a = Other (9), then ask SUT_5a1

SUT_5a1: Other: ______ *(write in)*

SUT_5b: How long did it take you to enroll with your most recent buprenorphine prescriber?
1. Hours
2. Days
3. Months
4. Over a year
5. Don't know (997)
6. Prefer not to say (999)

SUT_5c: Why did you start taking buprenorphine the most recent time? *(Select all that apply)*
1. To transition away from street-based opioids
2. To alleviate symptoms of withdrawal
3. To top up when there is not enough money for drug of choice
4. For pain management
5. To ensure consistency in my supply
6. To reduce drug use for health reasons/concerns
7. To sleep/come down
8. For compliance with court-order or legal mandate
9. Another reason, please specify: _______
10. Don't know (997)
11. Prefer not to say (999)

If SUT_5c = Another reason (9), then ask SUT_5c1

SUT_5c1: Another reason: ______ *(write in)*

SUT_5d: How much do you agree that, during the **last 6 months**, your buprenorphine prescriber(s) have respected you and your values, beliefs, and feelings?

1. Strongly agree
2. Agree
3. Neutral
4. Disaggree
5. Strongly disagree
6. Don't know (997)
7. Prefer not to say (999)

SUT_5e: Overall, how satisfied have you been with your buprenorphine prescriber(s) during the **last 6 months**?

1. Extremely satisfied
2. Very satisfied
3. Satisfied
4. Somewhat satisfied
5. Dissatisfied
6. Extremely dissatisfied
7. Don't know (997)
8. Prefer not to say (999)

If SUT_3b(2)=(1) and SUT_3b(3)=(2), ask SUT_5f

SUT_5f: Why did you stop receiving buprenorphine treatment? *(Select all that apply)*

1. Couldn't afford treatment
2. Not covered by health insurance
3. Completed planned treatment timeframe / duration
4. Transportation issues (e.g., too far away, no public transit available, transit is too expensive)
5. Difficulty scheduling appointments
6. Pregnant
7. Got incarcerated
8. Change my mind / did not want to continue in treatment
9. Discontinued by a treatment program / provider
10. Treatment wasn’t working / helping
11. Didn’t want to be seen getting treatment
12. Other, please specify:_____________

If SUT_5f = Other (12), then ask SUT_5f1

SUT_5f1: Other: ______ *(write in)*

If SUT_3c(2) = (1) ask:

SUT_6: How long have you been or were you on naltrexone the most recent time?

1. Less than one month
2. One to three months

3. Three to six months
4. Longer than six months
5. Don't know (997)
6. Prefer not to say (999)

SUT_6a: How did you find your current or most recent naltrexone prescriber? *(Select all that apply)*

1. Visited clinic (walk-in)
2. Made phone calls
3. Asked friends
4. Referred by a doctor, nurse, counselor, or other healthcare professional
5. Offered or referred to by an emergency department or hospital
6. Offered or referred to by an SSP or other community organization
7. Offered or referred to by community outreach worker
8. Offered or referred to by police
9. Other, please specify: ________
10. Don't know (997)
11. Prefer not to say (999)

If SUT_5g = Other (12), then ask SUT_5g1

SUT_5g1: Other: ______ *(write in)*

SUT_6b: How long did it take you to enroll with your most recent naltrexone prescriber?

1. Hours
2. Days
3. Months
4. Over a year
5. Don't know (997)
6. Prefer not to say (999)

SUT_6c: Why did you start taking naltrexone the most recent time? *(Select all that apply)*

1. To transition away from street-based opioids
2. To alleviate symptoms of withdrawal
3. For pain management
4. To reduce drug use for health reasons/concerns
5. To sleep/come down
6. For compliance with court-order or legal mandate
7. Another reason, please specify: _______
8. Don't know (997)
9. Prefer not to say (999)

If SUT_6c = Another reason (7), then ask SUT_6c1

SUT_6c1: Another reason: ______ *(write in)*

SUT_6d: How much do you agree that, during the **last 6 months**, your naltrexone prescriber(s) have respected you and your values, beliefs, and feelings?

1. Strongly agree
2. Agree

3. Neutral
4. Disaggree
5. Strongly disagree
6. Don't know (997)
7. Prefer not to say (999)

SUT_6e: Overall, how satisfied have you been with your naltrexone prescriber(s) during the **last 6 months**?

1. Extremely satisfied
2. Very satisfied
3. Satisfied
4. Somewhat satisfied
5. Dissatisfied
6. Extremely dissatisfied
7. Don't know (997)
8. Prefer not to say (999)

If SUT_3c(2)=(1) and SUT_3c(3)=(2), ask SUT_6f

SUT_6f: Why did you stop receiving naltrexone treatment? *(Select all that apply)*

1. Couldn't afford treatment
2. Not covered by health insurance
3. Completed planned treatment timeframe / duration
4. Transportation issues (e.g., too far away, no public transit available, transit is too expensive)
5. Difficulty scheduling appointments
6. Pregnant
7. Got incarcerated
8. Change my mind / did not want to continue in treatment
9. Discontinued by a treatment program / provider
10. Treatment wasn't working / helping
11. Didn't want to be seen getting treatment
12. Other, please specify:_____________

If SUT_6f = Other (12), then ask SUT_6f1

SUT_6f1: Other: ______ *(write in)*

If SUT_3n(2) = (1) ask:

SUT_7: How long have you been or were you on GLP-1s the most recent time?

1. Less than one month
2. One to three months
3. Three to six months
4. Longer than six months
5. Don't know (997)
6. Prefer not to say (999)

SUT_7a: How did you find your current or most recent GLP-1 prescriber? *(Select all that apply)*

1. Visited clinic (walk-in)

2. Made phone calls
3. Asked friends
4. Referred by a doctor, nurse, counselor, or other healthcare professional
5. Other, please specify: ________
6. Don't know (997)
7. Prefer not to say (999)

If SUT_7a = Other (5), then ask SUT_7a1

SUT_7a1: Other: ______ *(write in)*

SUT_7b: How long did it take you to enroll with your most recent GLP-1 prescriber?
1. Hours
2. Days
3. Months
4. Over a year
5. Don't know (997)
6. Prefer not to say (999)

SUT_7c: Why did you start taking GLP-1s the most recent time? *(Select all that apply)*
1. To transition away from street-based opioids
2. To alleviate symptoms of withdrawal
3. To reduce drug use for health reasons/concerns
4. Another reason, please specify: _______
5. Don't know (997)
6. Prefer not to say (999)

If SUT_7c = Another reason (4), then ask SUT_7c1

SUT_7c1: Other: ______ *(write in)*

SUT_7d: How much do you agree that, during the **last 6 months**, your GLP-1s prescriber(s) have respected you and your values, beliefs, and feelings?
1. Strongly agree
2. Agree
3. Neutral
4. Disaggree
5. Strongly disagree
6. Don't know (997)
7. Prefer not to say (999)

SUT_7e: Overall, how satisfied have you been with your GLP-1s prescriber(s) during the **last 6 months**?
1. Extremely satisfied
2. Very satisfied
3. Satisfied
4. Somewhat satisfied
5. Dissatisfied
6. Extremely dissatisfied
7. Don't know (997)

8. Prefer not to say (999)

If SUT_3n(2)=(1) and SUT_3n(3)=(2), ask SUT_7f

SUT_7f: Why did you stop receiving GLP-1 treatment? *(Select all that apply)*
1. Couldn't afford treatment
2. Not covered by health insurance
3. Completed planned treatment timeframe / duration
4. Transportation issues (e.g., too far away, no public transit available, transit is too expensive)
5. Difficulty scheduling appointments
6. Pregnant
7. Got incarcerated
8. Change my mind / did not want to continue in treatment
9. Discontinued by a treatment program / provider
10. Treatment wasn't working / helping
11. Didn't want to be seen getting treatment
12. Other, please specify:____________

If SUT_7f = Other (12), then ask SUT_7f1

SUT_7f1: Other: ______ *(write in)*

If SUT_3d(2) = (1) ask:

SUT_8: In the **last six months**, were you admitted into a hospital to detox from drugs?
1. Yes
2. No
3. Don't know (997)
4. Prefer not to say (999)

If SUT_8 ! = 1, then skip to SUT_8b;
Else ask

SUT_8a: In the **last six months**, how many nights did you spend in a hospital to detox from drugs?

_______ (*write in* #)
997. Don't know (997)
999. Prefer not to say (999)

SUT_8b: Overall, how satisfied have you been with your detox programs in the **last 6 months**?
1. Extremely satisfied
2. Very satisfied
3. Satisfied
4. Somewhat satisfied
5. Dissatisfied
6. Extremely dissatisfied
7. Don't know (997)
8. Prefer not to say (999)

If SUT_3e(2) = (1), ask SUT_9

SUT_9: How long have you been or were you in an outpatient drug treatment program in the **last six months?**

1. Less than one month
2. One to three months
3. Three to six months
4. Longer than six months
5. Don't know (997)
6. Prefer not to say (999)

SUT_9a: Overall, how satisfied have you been with your outpatient drug or alcohol treatment program in the **last 6 months**?

1. Extremely satisfied
2. Very satisfied
3. Satisfied
4. Somewhat satisfied
5. Dissatisfied
6. Extremely dissatisfied
7. Don't know (997)
8. Prefer not to say (999)

If SUT_3h(2) = (1), ask SUT_10

SUT_10: How long did you stay in a residential drug treatment program in the **last six months**?

1. Less than one month
2. One to three months
3. Three to six months
4. Longer than six months
5. Don't know (997)
6. Prefer not to say (999)

SUT_10a: Overall, how satisfied have you been with your residential drug treatment program in the **last 6 months**?

1. Extremely satisfied
2. Very satisfied
3. Satisfied
4. Somewhat satisfied
5. Dissatisfied
6. Extremely dissatisfied
7. Don't know (997)
8. Prefer not to say (999)

Prompt: Now, we want to learn a little more about things that might have made it difficult to get treatment.

SUT_11: During the **last 6 months**, was there a time when you tried to get treatment for alcohol or drug problems but could not get it?

1. Yes
2. No
3. Don't know (997)

4. Prefer not to say (999)

If SUT_11 ! = (1) Yes, skip to SUT_12;
Else ask

SUT_11a: Were you trying to access any treatment that might have been available, or were there specific types of treatment you were trying to get?

1. Any treatment
2. Specific type of treatment
3. Don't know (997)
4. Prefer not to say (999)

If SUT_11 ! = (2) Yes, skip to SUT_11c;
Else ask

SUT_11b: What kind of treatment did you try to access, but were unable to get? *(Select all that apply)*

1. Buprenorphine (Suboxone, Sublocade)
2. Methadone
3. Naltrexone (Vivitrol; extended-release naltrexone [XR-NTX] for alcohol use disorder)
4. Detox (detoxification)
5. Outpatient therapy or group sessions
6. Partial hospitalization (intensive outpatient)
7. Hospitalization (inpatient treatment)
8. Residential drug treatment program (not hospitalization)
9. Psychiatric inpatient treatment
10. Recovery/sober living housing
11. Mutual support group (like AA or NA)
12. Contingency management (getting an incentive for not using drugs)
13. Psychedelic therapy
14. GLP-1 medications for substance use disorder (e.g., Ozempic, Wegovy, Semaglutide)
15. Another type of treatment (Please specify)
16. Don't know (997)
17. Prefer not to say (999)

If SUT_11b = Another type of treatment (15), then ask SUT_11b1

SUT_11b1: Another type of treatment: ______ *(write in)*

SUT_11c: What caused the difficulty/barrier to accessing treatment? *(Select all that apply)*

1. Couldn't afford treatment
2. Not covered by health insurance
3. Could not find treatment or did not know where to go for treatment
4. Transportation issues (e.g., too far away, no public transit available, transit is too expensive)
5. Waitlist was too long (or am on waitlist still)
6. Difficulty scheduling appointments
7. I am not comfortable talking to my healthcare provider about these types of things (e.g., sex, drug use)
8. I do not think I should get treatment care while I am still using drugs.

9. Got incarcerated
10. Pregnant
11. I’m worried that people would judge me for getting treatment
12. Turned down by a care provider
13. Other, please specify:_____________
14. Don't know (997)
15. Prefer not to say (999)

If SUT_11b = Other (12), then ask SUT_11b1

SUT_11c1: Other: ______ *(write in)*

SUT_12: Have you ever been required to go to treatment by a court or other state authority when you did ***not*** choose to go voluntarily? This is sometimes called civil or involuntary commitment, court ordered treatment or hospitalization, or “Section 35” in Massachusetts.

1. Yes
2. No
3. Don't know (997)
4. Prefer not to say (999)

If SUT_12 != Yes (1), skip to HCV_1
Else ask

SUT_12a: Were you required to go to treatment by a court or other state authority when you did ***not*** choose to go voluntarily in the past six months?

1. Yes
2. No
3. Don't know (997)
4. Prefer not to say (999)

<u>HCV Prevention & Treatment</u>

Prompt: Next, we're going to talk about <u>hepatitis C Virus (also sometimes called HCV for short)</u>.

HCV_2. Have you ever been tested for hepatitis C?
1. Yes
2. No
3. Don't know (997)
4. Prefer not to say (999)

If HCV_2 = No (1), Don't know (997) (3) or Prefer not to say (999) (4), skip to STI_1;
Else ask

HCV_3. When was your last hepatitis C test?
1. Less than 3 months ago
2. 3 – 6 months ago
3. 6 – 12 months ago
4. More than 1 year ago
5. Don't know (997)
6. Prefer not to say (999)

HCV_4. Where was your last hepatitis C test done?
1. In a jail or prison
2. In an outpatient clinic (e.g., like in primary care)
3. In a hospital, as an inpatient
4. In an emergency department
5. In a syringe services program (SSP), otherwise known as a syringe exchange program
6. In another community-based program
7. Donating blood or plasma
8. Other
9. Don't know (997)
10. Prefer not to say (999)

HCV_5. Have you ever been told by a healthcare provider that you had hepatitis C?
1. Yes
2. No
3. Don't know (997)
4. Prefer not to say (999)

If HCV_5 = Yes (1), ask HCV_6;
Else skip to STI_1

HCV_6. Do you have hepatitis C right now?

1. Yes
2. No
3. Don't know (997)
4. Prefer not to say (999)

If HCV_6 = Yes (1) ask; else skip to HCV_7

HCV_6a. Is this your first time having hepatitis C?
1. Yes
2. No
3. Don't know (997)
4. Prefer not to say (999)

HCV_7. When were you last told by a healthcare provider that you had hepatitis C?
1. Within the last 6 months
2. Between 6 months and 1 year ago
3. Between 1 and 3 years ago
4. Between 3 and 5 years ago
5. Between 5 and 10 years ago
6. More than 10 years ago
7. Don't know (997)
8. Prefer not to say (999)

HCV_8. Have you ever been told by a healthcare provider that your body cleared hepatitis C on its own?
1. Yes
2. No
3. Don't know (997)
4. Prefer not to say (999)

HCV_9. Were you ever told by a healthcare provider that you were cured of hepatitis C?
1. Yes
2. No
3. Don't know (997)
4. Prefer not to say (999)

If HCV_9 = (1) Yes, ask HCV_10, else skip to HCV_11;

HCV_10. After being told that you were cured from a previous hepatitis C infection, have you ever been told again that you had been infected (that is, you had been re-infected with hepatitis C)?
1. Yes
2. No
3. Don't know (997)

4. Prefer not to say (999)

HCV_11. Have you ever been told by a healthcare provider that you should take medication to treat hepatitis C?
1. Yes
2. No
3. Don't know (997)
4. Prefer not to say (999)

HCV_12. Have you ever taken any medication to treat hepatitis C?
1. Yes
2. No
3. Don't know (997)
4. Prefer not to say (999)

PROMPT: Current HCV treatments are called direct-acting antivirals (DAAs). This is a daily, oral pill that is usually taken for 8 or 12 weeks and has few or no side effects. If taken as directed, most cases of hepatitis C can be cured. Some common DAAs are called Mavyret, Harvoni, or Epclusa.

HCV_13. Before today, had you heard of direct-acting antivirals (DAAs) to treat hepatitis C?
1. Yes
2. No
3. Don't know (997)
4. Prefer not to say (999)

If HCV_12 = Yes (1) and HCV 13 = Yes (1), ask HCV_14; else skip to STI_1

HCV_14. Have you ever received any direct-acting antiviral (DAA) treatment(s) for hepatitis C?
1. Yes
2. No
3. Don't know (997)
4. Prefer not to say (999)

If HCV_14 = Yes (1) , ask HCV_14a and HCV_15; else skip to HCV_16

HCV_14a. When was the last time you received any DAA treatment(s) for hepatitis C?
1. Within the last 6 months
2. Between 6 months and 1 year ago
3. Between 1 and 3 years ago
4. Between 3 and 5 years ago
5. Between 5 and 10 years ago
6. More than 10 years ago
7. Don't know (997)

8. Prefer not to say (999)

HCV_15. The last time you received DAA treatment for hepatitis C, did you complete that treatment?

1. Yes, I completed it
2. No, because I'm still on treatment
3. No, because I stopped treatment early
4. Don't know (997)
5. Prefer not to say (999)

HCV_16. The last time that you were told you had hepatitis C, what were the reason(s) that you did not use DAA treatment? *(Select all that apply)*

1. DAAs were not available when I was last diagnosed
2. I didn't think I needed it
3. I did not feel ready to start treatment
4. Thought I would forget to take it
5. Couldn't afford the costs of using DAAs (e.g., co-pays, labs)
6. Didn't know where to go to get DAAs
7. Didn't have transportation to get to the healthcare provider to get DAAs
8. Uncomfortable talking to a healthcare provider about these types of things (e.g., HCV, drug use)
9. Didn't think I should receive treatment while still using drugs
10. Tried to access DAAs, but a healthcare provider wouldn't prescribe them to me
11. Didn't know where to store DAAs so they would not be lost or stolen
12. Worried or felt nervous about side effects of DAAs
13. Worried about long-term effects of DAAs on my health
14. Worried that people would judge me for using DAAs
15. Worried that taking DAAs would impact my HIV or SUD treatment
16. Other, please specify: _____________
17. Don't know (997)
18. Prefer not to say (999)

If HCV_16 = Other (18), then ask HCV_16a

HCV_16a: Other: ______ *(write in)*

If HCV_14 = 1 (Yes) AND HCV_15 = 3 (No, because I stopped treatment early), ask HCV_17

HCV_17. The last time that you were told you had hepatitis C, what were the reason(s) that you did not complete DAA treatment? *(Select all that apply)*

1. Didn't think I needed it anymore
2. I just forget to take the pills
3. I went to jail/prison and couldn't take DAA while there
4. Couldn't afford the costs of using DAAs (e.g., co-pays, labs)

5. I have trouble getting transportation to the healthcare provider to keep getting DAAs
6. I am not comfortable talking to my healthcare provider about these types of things (e.g., sex, drug use)
7. Didn’t think I should keep taking DAAs while I was still using drugs
8. My healthcare provider wouldn’t prescribe DAAs to me anymore
9. My pills got lost or stolen
10. I have side effects that make me sick
11. I am worried about the long-term effects of DAAs on my health
12. I feel like people judge me for using DAAs
13. Worried that taking DAAs would impact my HIV or SUD treatment
14. Other, specify: ___________
15. Don't know (997)
16. Prefer not to say (999)

If HCV_17 = Other (14), then ask HCV_17a, else skip to STI_1
HCV_17a: Other: ______ *(write in)*

Sexual Health Block

STI Testing

Prompt: In this next section, we're going to talk about sexual health, starting with sexually transmitted infections.

STI_1. In your entire life, have you ever been tested for STIs such as gonorrhea, chlamydia, syphilis, etc.? *(Do not include HIV testing).*

1. Yes
2. No
3. Don't know (997)
4. Prefer not to say (999)

If STI_1 = (1) Yes, ask STI_1a, else skip to STI_2

STI_1a. In your entire life, have you ever tested positive for any STI?

1. Yes
2. No
3. Don't know (997)
4. Prefer not to say (999)

If STI_1a = (1) Yes, ask STI_1b, else skip to STI_1c

STI_1b. In your entire life, which of the following STIs have you tested positive for? *(Select all that apply)*

1. Chlamydia
2. Trichomonas (trich)
3. Gonorrhea (clap)
4. Syphilis
5. Genital or Anal Herpes
6. Genital or Anal Warts
7. Chancroid (sore or ulcer in or around the vagina or anus)
8. Pelvic inflammatory disease
9. Vaginal candidiasis (yeast infection)
10. Other, please specify:______
11. Don't know (997)
12. Prefer not to say (999)

If STI_1b = Other (10), then ask STI_1b1

STI_1b1: Other: ______ *(write in)*

STI_1c. In the **<u>last 6 months</u>**, have you been tested for any STI?

1. Yes
2. No
3. Don't know (997)
4. Prefer not to say (999)

If STI_1c = (1) Yes, ask STI_1d;
Else skip to STI_2

STI_1d. When was the last time you were tested for STIs? It's OK if you don't know the exact date. Please give your best guess.

MM/YYYY_______ (*write in*)
997. Don't know (997)
999. Prefer not to say (999)

STI_1e In the **last 6 months**, have you tested positive for any STIs?

1. Yes
2. No
3. Don't know (997)
4. Prefer not to say (999)

If STI_1e = 1 (Yes), ask STD_1f;
Else, skip to STI_2

STI_1f. Which of the following STIs did you test positive for in the **last 6 months**? *(Select all that apply)*

1. Chlamydia
2. Trichomonas (trich)
3. Gonorrhea (clap)
4. Syphilis
5. Genital or Anal Herpes
6. Genital or Anal Warts
7. Chancroid (sore or ulcer in or around the vagina or anus)
8. Pelvic inflammatory disease
9. Vaginal candidiasis (yeast infection)
10. Other, please specify: ________
11. Don't know (997)
12. Prefer not to say (999)

If STI_1f = Other (10), then ask STI_1f1

STI_1f1: Other: ______ *(write in)*

STI_1g. You indicated testing positive for the following STI(s) in the **last 6 months**. We would like to know if you have received treatment for these STI(s). For each of the STIs listed below, please indicate if you have received treatment in the last 6 months. *(Select all that apply)*

1. Chlamydia - Urethral (infection of your urethra or penis)
2. Chlamydia - Rectal (infection of your rectum or anus)
3. Chlamydia - Oral (infection of your throat or mouth)
4. Trichomonas (trich)
5. Gonorrhea (clap or drip) - Urethral (infection of your urethra or penis)
6. Gonorrhea (clap or drip) - Rectal (infection of your rectum or anus)
7. Gonorrhea (clap or drip) - Oral (infection of your throat or mouth)
8. Syphilis
9. Genital or Anal Herpes
10. Genital or Anal Warts
11. Chancroid (sore or ulcer in or around the vagina or anus)
12. Pelvic inflammatory disease
13. Vaginal candidiasis (yeast infection)
14. Other, please specify: ________
15. Prefer not to say (999)

If STI_1g = Other (14), then ask STI_1g1

STI_1g1: Other: ______ *(write in)*

STI_2. Before today, have you ever heard of people taking medicines <u>AFTER</u> sex to reduce the likelihood of getting gonorrhea, chlamydia or syphilis? This is called STI post-exposure prophylaxis, or DoxyPEP.

1. Yes
2. No
3. Don't know (997)
4. Prefer not to say (999)

STI_2a. Have you ever taken DoxyPEP <u>AFTER</u> sex, to reduce the likelihood of getting gonorrhea, chlamydia or syphilis?

1. Yes
2. No
3. Don't know (997)
4. Prefer not to say (999)

If STI_2a =(1) Yes, ask STI_2b; Else skip to HTS_1

STI_2b. In the last 6 months, have you taken DoxyPEP <u>AFTER</u> sex, to reduce the likelihood of getting gonorrhea, chlamydia or syphilis?

1. Yes
2. No
3. Don't know (997)
4. Prefer not to say (999)

HIV Testing and Prevention

Prompt: The following questions ask you about your past experience getting tested for HIV.

HTS_1: Have you ever been tested for HIV?
1. Yes
2. No
3. I'm not sure
4. Prefer not to say (999)

If HTS_1 = No (2), Don't know (997) (3), or Prefer not to say (999) (4), skip to HTS_5a; Else ask

HTS_2: Not including any HIV testing you have done for this study, when did you have your most recent HIV test?
1. 0-3 months ago
2. 3-6 months ago
3. 6-12 months ago
4. 12-18 months ago
5. 18-24 months ago
6. > 2 years ago
7. Don't know (997)
8. Prefer not to say (999)

HTS_2a: I'd just like to confirm: not including any HIV testing you have done for this study, in the **last 6 months** have you been tested for HIV?
1. Yes
2. No
3. Don't know (997)
4. Prefer not to say (999)

HTS_3: Have you ever used an HIV self-test kit (or “home test”) for yourself?
1. Yes
2. No
3. Don't know (997)
4. Prefer not to say (999)

If HTS_3 = (2) No OR (3) Don't know (997) OR (4) Prefer not to say (999), skip to HTS_4; Else ask

HTS_3a: Did you connect with a clinical provider during or after using an HIV self-test (for example, over the phone)?
1. Yes
2. No
3. Don't know (997)
4. Prefer not to say (999)

HTS_4: What is your current HIV status? *(Interviewer note: If 'I'm not sure' or 'Prefer not to say (999)' selected, treat as 'Not Living with HIV' for subsequent questions)*

0. Not living with HIV (HIV-negative)
1. Living with HIV (HIV-positive)
2. I'm not sure/Don't know (997)
3. Prefer not to say (999)

If HTS_4 = 1 (Living with HIV [HIV-positive]), skip to HCC_1 and do not ask HTS_4 through HTS_26 at all future study visits;
Else ask

**HIV Knowledge**			
**Prompt: For the next question, please choose True (T) or False (F) or Don't know (997) (DK) for the following statements. If you don't know, please don't guess — this isn't a test and it's really helpful for us to get an understanding of what's well known and what's not.**			
	**True**	**False**	**Don't know (997)**
HTS_5a: HIV can be transmitted through coughing and sneezing	1	2	997
HTS_5b: HIV can be transmitted through sharing cigarettes	1	2	997
HTS_5c: HIV can be transmitted by hugging an infected person	1	2	997
HTS_5d: HIV can be transmitted through toilet seats	1	2	997
HTS_5e: Mosquitoes can transmit HIV	1	2	997
HTS_5f: Transferring drugs from one syringe into another can transmit HIV	1	2	997
HTS_5g: HIV can be transmitted through drug injection "works," like cookers, cottons, or rinse water	1	2	997
HTS_5h: Condoms decrease the risk of HIV transmission when used correctly and consistently	1	2	997
HTS_5i: HIV can be transmitted from mother to baby	1	2	997
HTS_5j: HIV is transmitted through infected semen	1	2	997

HIV Risk Perceptions

**No chance at all**										**Very likely**
**0**	**10**	**20**	**30**	**40**	**50**	**60**	**70**	**80**	**90**	**100**

Prompt: For the next set of questions, please tell me, on a scale from 0 to 100%, what you think the chances are that the following things will happen over the next year.

HTS_6: On a scale from 0 to 100%, what is the chance that you will get HIV in the next year?

Write in #:__________ *(in 10% increments)*

Don't know (997)

Prefer not to say (999)

HTS_7: On a scale from 0 to 100%, what is the chance that you will get HIV in your lifetime?

Write in #:__________ *(in 10% increments)*

Don't know (997)

Prefer not to say (999)

Prompt: Pre-exposure prophylaxis, or PrEP, is used to *<u>prevent</u>* HIV. PrEP is a medication taken to prevent HIV.

HTS_11: Before joining this study, had you heard of PrEP?

1. Yes
2. No
3. Don't know (997)
4. Prefer not to say (999)

HTS_12a: Have you ever taken PrEP for HIV prevention?

1. Yes
2. No
3. Don't know (997)
4. Prefer not to say (999)

If HTS_12a= 2 (No), 3 (Don't know (997)) or 4 (Prefer not to say (999)) skip to HTS_13; Else ask

HTS_12b: Have you taken PrEP for HIV prevention in the **<u>past 6 months</u>**?

1. Yes
2. No
3. Don't know (997)
4. Prefer not to say (999)

If HTS_12b = 2 (No), 3 (Don't know (997)) or 4 (Prefer not to say (999)) skip to HTS_13; Else ask

HTS_12c: Are you currently taking PrEP for HIV prevention?

1. Yes
2. No
3. Don't know (997)
4. Prefer not to say (999)

HTS_13: Different types of PrEP are now available as pills and injections that work for different periods of time. Which type of PrEP would you prefer, if any?

1. None (I would not take any type of PrEP)
2. Daily oral pill
3. Pill before and after having sex (also known as on-demand PrEP or 2-1-1 PrEP)
4. Injection every two months
5. Injection every six months
6. Don't know (997)

7. Prefer not to say (999)

If HTS_12c = 1 (Yes), skip to HTS_17e;
Else ask

HTS_14: How interested are you in taking PrEP in the next **6 months**?
1. Very disinterested
2. Disinterested
3. Neutral
4. Interested
5. Very interested
6. Don't know (997)
7. Prefer not to say (999)

HTS_15: How likely are you to begin taking PrEP in the next **6 months**?
1. Extremely unlikely
2. Very unlikely
3. Somewhat likely
4. Very likely
5. Extremely likely
6. Don't know (997)
7. Prefer not to say (999)

If HTS_15 = 1 (Extremely unlikely), skip to HTS_17a;
Else ask

HTS_16: Where would you most prefer to get PrEP care?
1. Primary care clinic or health center
2. Substance use disorder treatment program or clinic
3. Pharmacy
4. Syringe services program (SSP), otherwise known as a syringe exchange program
5. HIV/STI clinic
6. Other, please specify: ____________
7. Don't know (997)
8. Prefer not to say (999)

If HTS_16 = Other (6), then ask HTS_16a

HTS_16a: Other: ______ (write in)

Barriers to PrEP Uptake

If HTS_12a = 1 (Yes), skip to HTS_17e
Else ask

HTS_17a: What are your reasons for not starting PrEP? *(Please select all that apply)*
1. Didn’t know about PrEP until today
2. I don’t think I need it
3. I am not ready to start PrEP yet
4. I think I would forget to take the medication

5. I don’t think I could afford the cost of PrEP (e.g., co-pays, labs)
6. I Don't know (997) where to go to get PrEP
7. I don’t have transportation to get to the healthcare provider to get PrEP
8. Uncomfortable talking to healthcare providers about these types of things (e.g., HIV, sex, drug use)
9. I do not think I should use PrEP while I am still using drugs
10. I tried to access PrEP, but my healthcare provider wouldn’t prescribe it to me
11. I Don't know (997) where to store my PrEP so it wouldn’t be lost or stolen
12. I am nervous about side effects of PrEP
13. I’m worried about the long-term effects of PrEP on my health
14. I’m worried that people would judge me for using PrEP
15. Other, please specify: ______
16. Prefer not to say (999)

If HTS_17a = Other (15), then ask HTS_17a1

HTS_17a1: Other: ______ (write in)

If HTS_12a = 1 (Yes) AND HTS_12c = 0 (Yes), skip to HTS_17f
Else ask HTS_17b and HTS_17c

HTS_17b: When did you stop using PrEP?

1. MM/YY: _________
2. Don't know (997)
3. Prefer not to say (999)

HTS_17c: Why did you stop using PrEP?

1. I did not think I needed it anymore
2. I kept forgetting to take it, so I just stopped
3. I went to jail/prison and couldn’t keep taking PrEP
4. Couldn’t afford the cost of using PrEP (e.g., co-pays, labs)
5. Uncomfortable talking to healthcare providers about these types of things (e.g., sex, drug use)
6. I did not think I should keep taking PrEP while I was still using drugs
7. My healthcare provider wouldn’t prescribe it to me anymore
8. My PrEP kept getting lost or stolen
9. I had side effects that made me sick
10. I was worried about the long-term effects of PrEP on my health
11. I felt like people judged me for using PrEP
12. Other, please specify: _______
13. Prefer not to say (999)

If HTS_17c = Other (12), then ask HTS_17c1

HTS_17c1: Other: ______ (write in)

If HTS_12a = 2 (No), then skip to HTS_24
Else if HTS_12a = 1 (Yes) AND HTS_12b = 1 (Yes) AND HTS_12c = 2 (No), then skip

to 17e
Else if HTS_12c = 1 (Yes), then skip to HTS_17f
Else if HTS_12a = 1 (Yes) AND HTS_12b= 2 (No), then ask

HTS_17d: The last time you used PrEP, how did you use it?
1. One PrEP pill once a day (also known as daily PrEP)
2. PrEP pills before and after sex (also known as 2-1-1 or on-demand PrEP)
3. A shot every 2 months (also known as Cabotegravir or Cabenuva)
4. A shot every 6 months (also known as Lenacapavir or Yeztugo)
5. Don't know (997)
6. Prefer not to say (999)

HTS_17e: How are you currently using PrEP?
1. One PrEP pill once a day (also known as daily PrEP)
2. PrEP pills before and after sex (also known as 2-1-1 or on-demand PrEP)
3. A shot every 2 months (also known as Cabotegravir or Cabenuva)
4. A shot every 6 months (also known as Lenacapavir or Yeztugo)
5. Don't know (997)
6. Prefer not to say (999)

If HTS_17d = 1 (One PrEP pill once a day) OR 17f = 1, skip to HTS_18

If HTS_17d = 2 (PrEP pills before and after sex) or 17f = 2, skip to HTS_19

If HTS_17d = 3 (A shot every 2 months) OR HTS_17f = 3, skip to HTS_20

If HTS_17d = 4 (A shot every 6 months) OR HTS_17f = 4, skip to HTS_21

**Daily PrEP**	
HTS_18: During the **last 6 months**, did you ever miss 4 or more doses in a week?	1. Yes<br>2. No<br>3. Don't know (997)<br>4. Prefer not to say (999)
If HTS_18 = Yes (1), then ask HTS_18a; else skip to HTS_22<br><br>HTS_18a: During the last **6 months**, about how many times did you miss 4 or more doses in a week?	*(Dropdown of numbers 1 to 26)*<br>1. Don't know (997)<br>2. Prefer not to say (999)

**2-1-1- PrEP**	
HTS_19: In the **last 7 days**, on how many days did you take any PrEP pills?	*Dropdown list 0-7*<br>997. Don't know (997)<br>999. Prefer not to say (999)

HTS_19a: During the **last 6 months**, did you ever NOT take your complete 2-1-1 regimen when having condomless sex?	1. Yes<br>2. No<br>3. Don't know (997)<br>4. Prefer not to say (999)
If HTS_19a = Yes (1), then ask HTS_19b; else skip to HTS_22<br>HTS_19b: During the **last 6 months**, about how many times did you miss your complete 2-1-1 regimen when having condomless sex?	*Dropdown of numbers 1 to 52*<br>997. Don't know (997)<br>999. Prefer not to say (999)

**Cabotegravir/shot every 2 months**	
HTS_20: When did you get your most recent injection? It's OK if you don't know the exact date. Please provide your best guess.	1. [Month] [Day] [Year]<br>2. Don't know (997)<br>3. Prefer not to say (999)
HTS_20a: When is the date of your next PrEP injection? It's OK if you don't know the exact date. Please provide your best guess.	1. [Month] [Day] [Year]<br>2. I am not planning to get another PrEP injection<br>3. Don't know (997)<br>4. Prefer not to say (999)
HTS_20b: In the **last 6 months**, have you ever gone more than 3 months between PrEP injections?<br>***If HTS_20b = 2 (No), skip to HTS_22***	1. Yes<br>2. No<br>3. Don't know (997)<br>4. Prefer not to say (999)
HTS_20c: In the **last 6 months**, what challenges have gotten in the way of you getting your PrEP injections? *(Select all that apply)*	1. I am not sure I need PrEP anymore.<br>2. I just forget to go to get my PrEP injection<br>3. I thought I could miss an injection and still be protected<br>4. I went to jail/prison and missed my PrEP injection while there<br>5. I can not afford the costs of using PrEP (e.g., co-pays, labs).<br>6. I have trouble getting to the healthcare provider to keep getting PrEP<br>7. I am not comfortable talking to my healthcare provider about these types of things (e.g., sex, drug use)<br>8. I do not think I should get PrEP injections while I am still using drugs.<br>9. My healthcare provider wouldn't prescribe it to me anymore.<br>10. I have side effects that make me sick, or injection site reactions that I don't like.

	11. I am about the long-term effects of PrEP on my health.<br>12. I feel like people judge me for using PrEP.<br>13. Other, specify ____________<br>14. Don't know (997)<br>15. Prefer not to say (999)

**Lenacapavir/shot every 6 month**	
HTS_21: When did you get your most recent injection? It's OK if you don't know the exact date. Please provide your best guess.	1. [Month] [Day] [Year]<br>2. Don't know (997)<br>3. Prefer not to say (999)
HTS_21a: What is the date of your next PrEP injection? It's OK if you don't know the exact date. Please provide your best guess.	1. [Month] [Day] [Year]<br>2. I am not planning to get another PrEP injection<br>3. Don't know (997)<br>4. Prefer not to say (999)
HTS_21b: What challenges have gotten in the way of you getting your PrEP injections? *(Select all that apply)*	1. I haven't had any challenges<br>2. I am not sure I need PrEP anymore.<br>3. I just forget to go to get my PrEP injection<br>4. I thought I could miss an injection and still be protected<br>5. I went to jail/prison and missed my PrEP injection while there<br>6. I can not afford the costs of using PrEP (e.g., co-pays, labs).<br>7. I have trouble getting to the healthcare provider to keep getting PrEP<br>8. I am not comfortable talking to my healthcare provider about these types of things (e.g., sex, drug use)<br>9. I did not think I should keep getting PrEP injections while I was still using drugs.<br>10. My healthcare provider wouldn't prescribe it to me anymore.<br>11. I have side effects that make me sick, or injection site reactions that I don't like.<br>12. I am about the long-term effects of PrEP on my health.<br>13. I feel like people judge me for using PrEP.<br>14. Other, please specify ____________<br>15. Don't know (997)<br>16. Prefer not to say (999)
If HTS_21b = Other (14), then ask HTS_21b1<br><br>***HTS_21b1***: Other: ______ *(write in)*	

HTS_22: When was your most recent PrEP care medical appointment? It's OK if you don't remember the exact date. Please provide your best guess

1. [Month] [Day] [Year]
2. Don't know (997)
3. Prefer not to say (999)

Barriers to Regular PrEP Use

If HTS_17e or HTS_17f = 1 (One PrEP pill once a day [also known as daily PrEP]) OR or 2 (PrEP pills before and after sex [also known as 2-1-1 or on-demand PrEP]) AND (HTS_18a = (1) or HTS_19b = (1)), then ask HTS_23;
Else skip to HTS_24

HTS_23: What has gotten in the way of you taking your PrEP doses as prescribed?
(Select all that apply)

1. Didn't think I needed it anymore
2. I just forget to take the pills
3. I went to jail/prison and couldn't take PrEP while there
4. Couldn't afford the costs of using PrEP (e.g., co-pays, labs)
5. I have trouble getting transportation to the healthcare provider to keep getting PrEP
6. I am not comfortable talking to my healthcare provider about these types of things (e.g., sex, drug use)
7. Didn't think I should keep taking PrEP while I am still using drugs
8. My healthcare provider wouldn't prescribe it to me anymore
9. My PrEP gets lost or stolen
10. I have side effects that make me sick
11. I am worried about the long-term effects of PrEP on my health
12. I feel like people judge me for using PrEP
13. Other, please specify: ______
14. Don't know (997)
15. Prefer not to say (999)

If HTS_23 = Other (13), then ask HTS_23a

HTS_23a: Other: ______ *(write in)*

Post-Exposure Prophylaxis (PEP) and Awareness and Use

HTS_24: Before today, have you ever heard of people taking HIV prevention medicines AFTER a sexual or drug use exposure, to reduce the likelihood of getting HIV? This is called post-exposure prophylaxis, or PEP.

1. Yes
2. No
3. Don't know (997)
4. Prefer not to say (999)

HTS_25: Have you ever taken post-exposure prophylaxis (PEP) AFTER a sexual or drug use exposure, to reduce the likelihood of getting HIV?

1. Yes
2. No
3. Don't know (997)
4. Prefer not to say (999)

If HTS_25 = (1), ask HTS_26
Else skip to SRB_1

HTS_26: In the **last 6 months**, have you taken PEP (post-exposure prophylaxis) AFTER a sexual or drug use exposure, to reduce the likelihood of getting HIV?

1. Yes
2. No
3. Don't know (997)
4. Prefer not to say (999)

HIV Care Cascade

If HTS_4 = 1 (Not living with HIV), 3 (I'm not sure/Don't know (997)) OR 4 (Prefer not to say (999)), then skip to SRB_1;
Else ask HCC_1 (Ask HCC_1 through HCC_2c only the first time participant reports living with HIV)

HCC_1: When did you first find out you were living with HIV from an HIV test?
It's OK if you don't know the exact date. Please give your best guess.
1. [Month] [Day] [Year]
2. Don't know (997)
3. Prefer not to say (999)

HCC_2: Have you ever been seen by a healthcare provider for care related to your HIV diagnosis?
1. Yes
2. No
3. Don't know (997)
4. Prefer not to say (999)

If HCC_2 = 1 (Yes), skip to HCC_2b;
Else ask

HCC_2a: What are your reasons for not ever seeing a healthcare provider for HIV care? *(Select all that apply)*
1. I did not think I needed it
2. I am not ready yet.
3. Couldn't afford care
4. Not covered by health insurance
5. Could not find treatment or did not know where to go for treatment
6. Transportation issues (e.g., too far away, no public transit available, transit is too expensive)
7. I am not comfortable talking to my healthcare provider about these types of things (e.g., sex, drug use)
8. Waitlist was too long (or am on waitlist still)
9. Difficulty scheduling appointments
10. I do not think I should get HIV care while I am still using drugs.
11. Got incarcerated
12. I'm worried that people would judge me for having HIV.
13. Turned down by a care provider
14. Other, please specify:_____________
15. Don't know (997)
16. Prefer not to say (999)

If HCC_2a = Other (14), then ask HCC_2a1
Else skip to HCC_5

HCC_2a1: Other: ______ *(write in)*

If HCC_2 = 2 (No), skip to HCC_5

HCC_2b: When did you first go to a healthcare provider after learning you were living with HIV? It's OK if you don't know the exact date. Please give your best guess.

1. [Month] [Day] [Year]
2. Don't know (997)
3. Prefer not to say (999)

HCC_2c: Was there a time after you first engaged in HIV care that you went 6 months or longer without a medical visit with your HIV care provider?

1. Yes
2. No
3. Don't know (997)
4. Prefer not to say (999)

HCC_2d: When did you last go to a healthcare provider for HIV care? It's OK if you don't know the exact date. Please give your best guess.

1. [Month] [Day] [Year]
2. Don't know (997)
3. Prefer not to say (999)

HCC_3: In the **last 6 months**, have you had an appointment with your HIV care provider?

1. Yes
2. No
3. Don't know (997)
4. Prefer not to say (999)

HCC_4: Do you have a scheduled visit with your HIV provider in the **next 6 months**?
By scheduled appointments, we mean routine appointments, not walk-in or urgent care appointments for acute/urgent issues.

1. Yes
2. No
3. Don't know (997)
4. Prefer not to say (999)

HCC_5: Have you ever had an HIV viral load test?

1. Yes
2. No
3. Don't know (997)
4. Prefer not to say (999)

If HCC_5 = (2) No, (3) Don't know (997), or (4) Prefer not to say (999), skip to HCC_6
Else ask

HCC_5a: Have you had an HIV viral load test within the **last 6 months**?

1. Yes
2. No
3. Don't know (997)
4. Prefer not to say (999)

HCC_5b: What was the result of your most recent viral load test? *Please select one of the answers that most closely matches your last viral load test results in viral copies/mL.*

1. Below the level of detection, undetectable
2. Detectable, but less than 500 copies/mL
3. Greater than 500 copies/mL
4. Don't know (997)
5. Prefer not to say (999)

HCC_6: Have you ever had a CD4 count test?

1. Yes
2. No
3. Don't know (997)
4. Prefer not to say (999)

If HCC_6 = (2) No, (3) Don't know (997), or (4) Prefer not to say (999), skip to HCC_7;
Else ask

HCC_6a: Have you ever had a CD4 count less than or equal to 200 cells/mm^3?

1. Yes
2. No
3. Don't know (997)
4. Prefer not to say (999)

If HCC_2 = 2 (No), skip to HCC_10;
Else ask

HCC_7: Have you ever taken pills or an injection that were prescribed by a healthcare provider to <u>treat</u> your HIV (antiretroviral therapy [ART], not medications you are taking to prevent other complications of HIV)?

1. Yes
2. No
3. Don't know (997)
4. Prefer not to say (999)

If HCC_7 = (1) Yes, skip to HCC_8;
Else ask

HCC_7a: What are the reasons that you have never taken ART to treat your HIV? *(Select all that apply)*

1. I had not heard about ART until today.
2. I don't think I need it.
3. I am not ready yet.
4. I think I would forget to take it.
5. I don't think I could afford the cost of ART
6. I Don't know (997) where to go to get ART
7. I don't have transportation to get to the healthcare provider to get ART
8. I am not comfortable talking to my healthcare provider about these types of things (e.g., sex, drug use)

9. I do not think I should take ART while I am still using drugs.
10. I tried to access ART, but my healthcare provider wouldn't prescribe it to me.
11. I do not know where I would keep my ART so it is not lost or stolen.
12. I am nervous about side effects of ART that might make me sick.
13. I'm worried about the long-term effects of ART on my health.
14. I'm worried that people would judge me for using ART.
15. Other, please specify: ________
16. Prefer not to say (999)

If HCC_7a = Other (15), then ask HCC_7a1

HCC_7a1: Other: ______ *(write in)*

If HCC_7 != (1) Yes, skip to HCC_10;
Else ask

HCC_8: Are you currently taking ART to treat your HIV?
1. Yes
2. No
3. Don't know (997)
4. Prefer not to say (999)

If HCC_8 = Yes (1) skip to HCC_8c;
Else ask

HCC_8a: When did you stop using ART (the most recent time if more than once)? It's OK if you don't know the exact date. Please give your best guess.
1. [Month] [Day] [Year]
2. Prefer not to say (999)

HCC_8b: Why did you stop using ART (the most recent time if more than once)?
1. I did not think I needed it anymore.
2. I kept forgetting, so I just stopped
3. I went to jail/prison and couldn't keep taking ART
4. I could not afford the costs of using ART (e.g., co-pays, labs).
5. I couldn't get to the healthcare provider to keep getting ART
6. I was not comfortable talking to my healthcare provider about these types of things (e.g., HIV, sex, drug use)
7. I did not think I should keep taking ART while I was still using drugs.
8. My healthcare provider wouldn't prescribe it to me anymore.
9. My ART kept getting lost or stolen.
10. I had side effects that made me sick.
11. I was worried about the long-term effects of ART on my health.
12. I felt like people judged me for using ART.
13. Other, please specify: _______
14. Prefer not to say (999)

If HCC_8b = Other (13), then ask HCC_8b1

HCC_8b1: Other: ______ *(write in)*

If HCC_8 != Yes (1), skip to HCC_9;
Else ask

HCC_8c: When did you first start taking HIV medications? It's OK if you don't know the exact date. Please give your best guess.
1. [Month] [Day] [Year]
2. Prefer not to say (999)

HCC_8d: How many times during the day has your HIV provider told you to take a dose of medicine (pills or other medicines) to treat your HIV?
1. 1 time a day
2. 2 times a day
3. 3 or more times a day
4. I am receiving long acting injectable ART
5. Don't know (997)
6. Prefer not to say (999)

If HCC_8d ! = 4 (I am receiving long-acting injectable ART), then skip to HCC_8g;
Else ask

HCC_8e: In the **last 6 months**, did you ever miss a scheduled injection?
1. Yes
2. No
3. Don't know (997)
4. Prefer not to say (999)

HCC_8f: If taking-long acting ART, when did you receive your last injection?
It's OK if you don't know the exact date. Please give your best guess.
1. [Month] [Day] [Year]
2. Don't know (997)
3. Prefer not to say (999)

If HCC_8d = 4 (I am receiving long acting injectable ART), skip to HCC_9;
Else ask

HCC_8g: In the **last 6 months**, what percent of your prescribed HIV medication have you taken? *0% means you have taken no medication, 50% means you have taken half your medication, 100% means you have taken every single dose of your medication. If you are unsure, make a best guess.*

Visual Analog Scale:

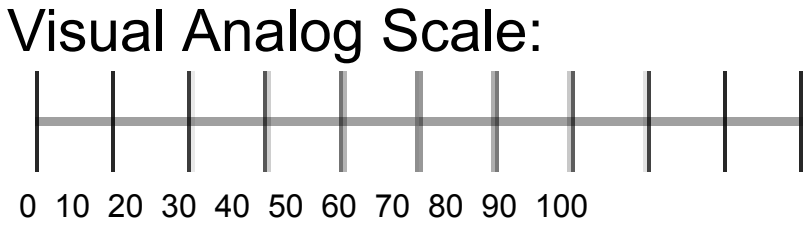

1. 0%
2. 10%
3. 20%
4. 30%

5. 40%
6. 50%
7. 60%
8. 70%
9. 80%
10. 90%
11. 100%
12. Don't know (997)
13. Prefer not to say (999)

HCC_8h: In the **last 6 months**, on how many days did you miss at least one dose of any of your HIV medicines?

Write in #: _______ (*should not be greater than 180*)
997. Don't know (997)
999. Prefer not to say (999)

HCC_8i: During the **last week**, how many days have you missed taking all your HIV medications?

Dropdown list: 0-7
997. Don't know (997)
999. Prefer not to say (999)

Situational HIV Medication Adherence Self-efficacy

**Prompt: Now we will ask you about situations that could occur during your treatment for HIV.**<br><br>**How confident are you that you can take your HIV medications exactly as prescribed in the following situations?**<br>**Use the response scale ranging from 1-Not at all confident to 5-Completely confident**<br><br>**How confident are you that you can take your HIV medications as prescribed…**								
HCC_9		1<br>Not at all confident	2<br>Slightly confident	3<br>Moderately confident	4<br>Very confident	5<br>Completely confident	Don't know (997)	Prefer not to say (999)
HCC_9a	Even when your daily routine changes or life feels chaotic?						997	999
HCC_9b	Even when you are using drugs or alcohol?						997	999
HCC_9c	Even when you are experiencing cravings or withdrawal symptoms?						997	999
HCC_9d	Even when you are staying in different places or do not have stable housing?						997	999
HCC_9e	Even when you do not have a safe or private place to keep your medications?						997	999
HCC_9f	Even when you feel depressed, anxious, stressed, or overwhelmed?						997	999
HCC_9g	Even when you are worried about others seeing your medications or judging you?						997	999
HCC_9h	Even when you are unable to get to a pharmacy, clinic, or healthcare						997	999

	appointment?							
HCC_9i	Even when your phone, belongings, or medications are lost, stolen, or unavailable?						997	999
HCC_9j	Even when you miss a dose or fall behind on your medications?						997	999

HIV Stigma

If HTS_4 = 2 (Living with HIV), then ask:<br><br>**In the last 6 months:**					
		1.	2.	997.	999.
		**Yes**	**No**	**Don't know (997)**	**Prefer not to say (999)**
HCC_10.	People have talked badly about you because of your HIV status.				
HCC_11.	Someone else disclosed your HIV status without your permission.				
HCC_12.	You have been verbally insulted, harassed, or threatened because of your HIV status.				

Sexual Risk Behaviors

PROMPT: Now I am going to ask you about your sexual behaviors. Some of these questions deal with sensitive topics and may make you uncomfortable. Please remember that this is completely confidential and you can always tell me if you want to skip a question, okay?

SRB_1. Have you ever had anal or vaginal sex with somebody else? *By anal sex, we mean a penis going into an anus. By vaginal sex, we mean a penis going into a vagina.*

1. Yes
2. No
3. Don't know (997)
4. Prefer not to say (999)

If SRB_1 = No (1), skip to HQL_1
Else ask

SRB_2. How old were you when you had anal or vaginal sex for the first time with somebody else?

1. (Age): _____
2. Don't know (997)
3. Prefer not to say (999)

SRB_3. In the **<u>last 6 months</u>**, have you had anal or vaginal sex? *(Reminder: By anal sex, we mean a penis going into an anus. By vaginal sex, we mean a penis going into a vagina.)*

1. Yes
2. No
3. Don't know (997)
4. Prefer not to say (999)

If SRB_3 = No (2) or Prefer not to say (999) (3), skip to HQL_1
Else ask

SRB_3a. In the **<u>last 6 months</u>**, with whom have you had anal or vaginal sex? *(Select all that apply)*

1. Cisgender women (not trans)
2. Cisgender men (not trans)
3. Transgender women (male-to-female/MTF)
4. Non-binary/gender variant, transgender person (male assigned sex at birth)
5. Transgender men (female-to-male/FTM)
6. Non-binary/gender variant, transgender person (female assigned sex at birth)
7. Intersex person
8. Don't know (997)
9. Prefer not to say (999)

SRB_4. In the **last 6 months**, how many PEOPLE have you had vaginal or anal sex with? It's OK if you don't know the exact number. Please give your best guess.

1. (write in #): _______
2. Don't know (997)
3. Prefer not to say (999)

SRB_5. Did you have vaginal or anal sex with any of these people without a condom?

1. Yes
2. No
3. Don't know (997)
4. Prefer not to say (999)

If SRB_5 = 1 (Yes), ask SRB_5a
Else skip to SRB_ 8

SRB_5a. How many people did you have vaginal or anal sex with without a condom?

1. (write in #): _______
2. Don't know (997)
3. Prefer not to say (999)

SRB_6. Among the people with whom you had anal or vaginal sex without a condom in the **last 6 months**, what was their HIV status?

1. All were HIV negative
2. All had unknown HIV status
3. All were people with HIV
4. HIV negative and HIV status unknown
5. HIV negative and people with HIV
6. HIV status unknown and people with HIV
7. HIV negative, HIV status unknown, and people with HIV
8. Don't know (997)
9. Prefer not to say (999)

Condom Use Frequency<br><br>SRB_7. While looking at the scale below, what percent of time did you use a condom when having vaginal or anal sex in the **last 6 months**?<br><br>*0% would mean 'NONE' of the time and 100% would mean 'ALL' of the time. If you are unsure, make a best guess.*<br><br>Visual Analog Scale:<br>0 10 20 30 40 50 60 70 80 90 100	1. 0%<br>2. 10%<br>3. 20%<br>4. 30%<br>5. 40%<br>6. 50%<br>7. 60%<br>8. 70%<br>9. 80%<br>10. 90%<br>11. 100%<br>12. Don't know (997)

	13. Prefer not to say (999)

Drug Use During Intercourse<br><br>SRB_8. While looking at the scale below, what percent of time did you drink alcohol or use drugs before you had anal or vaginal sex in the **last 6 months**?<br><br>*0% would mean 'NONE' of the time and 100% would mean 'ALL' of the time. If you are unsure, make a best guess.*<br><br>Visual Analog Scale:<br>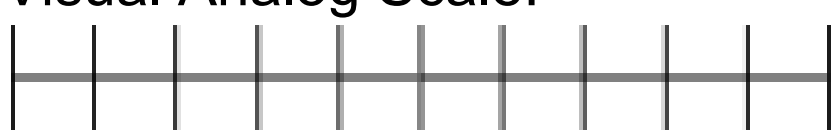<br>0 10 20 30 40 50 60 70 80 90 100	1. 0%<br>2. 10%<br>3. 20%<br>4. 30%<br>5. 40%<br>6. 50%<br>7. 60%<br>8. 70%<br>9. 80%<br>10. 90%<br>11. 100%<br>12. Don't know (997)<br>13. Prefer not to say (999)

Prompt: For these next few questions, when I say "sex," I will be asking about vaginal, anal, or oral sex. *(Reminder: By anal sex, we mean a penis going into an anus. By vaginal sex, we mean a penis going into a vagina. By oral sex, we mean using the mouth, lips, or tongue to stimulate a partner's penis, vagina, or anus.)*

SRB_10. In the **last 6 months**, have you received money, drugs, a place to stay, other things of value, or any favors (e.g. transportation, protection) in exchange for anal, vaginal, or oral sex?

1. Yes
2. No
3. Don't know (997)
4. Prefer not to say (999)

If SRB_10 = 2 (No), 3 (Don't know (997)) OR 4 (Prefer not to say (999)), skip to SRB_11 Else ask

SRB_10b. In the **last 6 months**, what specifically have you received in exchange for having anal, vaginal, or oral sex with someone else? *(Select all that apply)*

1. Currency (i.e., Cash)
2. Drugs
3. Alcohol
4. Housing
5. Transportation
6. Protection/Security
7. Clothing/Jewelry
8. Other: ________
9. Don't know (997)
10. Prefer not to say (999)

If SRB_10b= Other (8), then ask SRB_10b1

SRB_10b1: Other: ______ *(write in)*

SRB_11. In the **last 6 months**, have you given money, drugs, a place to stay, other things of value, or any favors (e.g. transportation, protection) in exchange for anal, vaginal, or oral sex?

1. Yes
2. No
3. Don't know (997)
4. Prefer not to say (999)

If SRB_11 = 1 (Yes), ask SRB_11a
Else skip to HQL_1

SRB_11a. In the **last 6 months**, what specifically have you given in exchange for them having anal, vaginal, or oral sex with you? *(Select all that apply)*

1. Currency (i.e., Cash)
2. Drugs
3. Alcohol
4. Housing
5. Transportation
6. Protection/Security
7. Clothing/Jewelry
8. Other: _______
9. Don't know (997)
10. Prefer not to say (999)

If SRB_11a= Other (8), then ask SRB_11a1

SRB_11a1: Other: ______ *(write in)*

Health Block

Health Status and Quality of Life

Optional Prompt: Great — thank you so much for talking with me about that. Now, we're going to talk about your health more generally.

HQL_1: In general, would you say your quality of life is…

1. Excellent
2. Very good
3. Good
4. Fair
5. Poor
6. Don't know (997)
7. Prefer not to say (999)

HQL_2: Rate your health today using the scale below:

Write in # on a scale of 0 to 100 in 10 increments: ______
Don't know (997)
Prefer not to say (999)

0 --100
Worst imaginable health   Best imaginable health

HQL_3: How is your mobility today?

1. I have no problems in walking about
2. I have slight problems in walking about
3. I have moderate problems in walking about
4. I have severe problems in walking about
5. I am unable to walk about
6. Don't know (997)
7. Prefer not to say (999)

HQL_4: How is your self-care today? *By self-care we mean washing or dressing yourself*

1. I have no problems washing or dressing myself
2. I have slight problems washing or dressing myself
3. I have moderate problems washing or dressing myself
4. I have severe problems washing or dressing myself
5. I am unable to wash or dress myself
6. Don't know (997)
7. Prefer not to say (999)

HQL_5: Were you able to complete your usual activities today (e.g., work, study, housework, family or leisure) today?

1. I have no problems doing my usual activities

2. I have slight problems doing my usual activities
3. I have moderate problems doing my usual activities
4. I have severe problems doing my usual activities
5. I am unable to do my usual activities
6. Don't know (997)
7. Prefer not to say (999)

Prompt: The following question refers to your overall sleep quality for the majority of nights in the past 7 days only. Please thinking about the quality of your sleep overall, such as how many hours of sleep you got, how easily you fell asleep, how often you woke up during the night (except to go to the bathroom), how often you woke up earlier than you had to in the morning, and how refreshing your sleep was.

HQL_6: During the last 7 days, how would you rate your overall sleep quality?

0 1 2 3 4 5 6 7 8 9 10
Terrible | Poor | Fair | Good | Excellent

Write in #: ______
Don't know (997)
Prefer not to say (999)

Prompt: We are going to ask you a few questions about pain.

HQL_7: What number best describes your pain on average in the last week?

0 1 2 3 4 5 6 7 8 9 10

No Pain Worse Pain Imaginable

**In the last week…**								
HQL_8.		1.	2.	3.	4.	5.	997.	999.
		Never	Almost Never	Some-times	Fairly Often	Very Often	Don't know (997)	Prefer not to say (999)
HQL_8a.	How difficult was it for you to take in new information because of pain?							
HQL_8b.	How much did pain interfere with your ability to concentrate?							
HQL_8c.	How much did pain interfere with your day to							

	day activities?							
HQL_8d.	How often did pain make you feel discouraged?							
HQL_8e.	How often did pain prevent you from standing for more than 30 minutes?							

Healthcare Access

Prompt: In this next section, I will be asking about how often you use different healthcare services. All of these questions will be about the last month only.

During the last month, how many . . .		997. Don't know (997)<br>999. Prefer not to say (999)
HCA_1.	…times have you had to go to an emergency room without being admitted to the hospital?	_______ times
HCA_2.	…times have you been hospitalized/admitted to the hospital (for example, had to stay overnight or were moved to another part of the hospital)?	_______ times
HCA_3.	…times have you visited a primary care provider for a wellness visit, physical, or general purpose check-up	_______ times
If HCA_3 = Zero (0) or Don't know (997) or Prefer not to say (999), skip to HCA_4<br>Else ask<br><br>How many of these times …		
HCA_3a.	…did you go in person?	_______ times
HCA_3b.	…did you see a doctor?<br>*(Interviewer: if needed, prompt "by doctor we mean someone who could prescribe you medication")*	_______ times
HCA_4.	…times have you seen a psychiatrist (MD) or psychologist (Ph.D., PsyD.) *By psychiatrist, we mean someone who could prescribe you mental health medication.*	_______ times
If HCA_4 = Zero (0) or Don't know (997) or Prefer not to say (999), skip to HCA_5<br>Else ask<br><br>How many of these times …		
HCA_4a.	...did you go in person?	_______ times
HCA_5.	...times have you seen any other kind of counselor or social worker?	_______ times
If HCA_5 = Zero (0) or Don't know (997) or Prefer not to say (999), skip to HCA_6a<br>Else ask<br><br>How many of these times …		
HCA_5a.	...did you go in person?	_______ times

If HCA_1 > 0, ask HCA_6a through HCV_6c

HCA_6a. Thinking about the last time you were at the emergency room, how was your overall experience getting treatment?
1. Extremely positive
2. Positive
3. Neutral
4. Negative
5. Extremely negative
6. Don't know (997)
7. Prefer not to say (999)

HCA_6b. Thinking about the last time you were at the emergency room, how much do you agree that the providers have respected you and your values, beliefs, and feelings?
1. Strongly agree
2. Agree
3. Neutral
4. Disaggree
5. Strongly disagree
6. Don't know (997)
7. Prefer not to say (999)

HCA_6c. Overall, how satisfied were you with the care you received at your last emergency room visit?
1. Extremely satisfied
2. Very satisfied
3. Satisfied
4. Somewhat satisfied
5. Dissatisfied
6. Extremely dissatisfied
7. Don't know (997)
8. Prefer not to say (999)

If HCA_3 > 0, ask HCA_7a through 7c

HCA_7a. Thinking about the last time you were getting primary care, how was your overall experience?
1. Extremely positive
2. Positive
3. Neutral
4. Negative
5. Extremely negative
6. Don't know (997)
7. Prefer not to say (999)

HCA_7b. Thinking about the last time you were getting primary care, how much do you agree that the providers have respected you and your values, beliefs, and feelings?
1. Strongly agree
2. Agree
3. Neutral
4. Disaggree
5. Strongly disagree

6. Don't know (997)
7. Prefer not to say (999)

HCA_7c. Overall, how satisfied were you with the care you received at your last primary care visit?
1. Extremely satisfied
2. Very satisfied
3. Satisfied
4. Somewhat satisfied
5. Dissatisfied
6. Extremely dissatisfied
7. Don't know (997)
8. Prefer not to say (999)

If HCA_4 > 0, ask HCA_8a through 8c

HCA_8a. Thinking about the last time you met with a psychiatrist, how was your overall experience?
1. Extremely positive
2. Positive
3. Neutral
4. Negative
5. Extremely negative
6. Don't know (997)
7. Prefer not to say (999)

HCA_8b. Thinking about the last time you met a psychiatrist, how much do you agree that the providers have respected you and your values, beliefs, and feelings?
1. Strongly agree
2. Agree
3. Neutral
4. Disaggree
5. Strongly disagree
6. Don't know (997)
7. Prefer not to say (999)

HCA_8c. Overall, how satisfied were you with the care you received at your last psychiatric visit?
1. Extremely satisfied
2. Very satisfied
3. Satisfied
4. Somewhat satisfied
5. Dissatisfied
6. Extremely dissatisfied
7. Don't know (997)
8. Prefer not to say (999)

If HCA_5 > 0, then ask HCA_9a through 9c

HCA_9a. Thinking about the last time you met with a counselor or social worker, how was your overall experience?

1. Extremely positive
2. Positive
3. Neutral
4. Negative
5. Extremely negative
6. Don't know (997)
7. Prefer not to say (999)

HCA_9b. Thinking about the last time you met a counselor or social worker, how much do you agree that the providers have respected you and your values, beliefs, and feelings?
1. Strongly agree
2. Agree
3. Neutral
4. Disaggree
5. Strongly disagree
6. Don't know (997)
7. Prefer not to say (999)

HCA_9c. Overall, how satisfied were you with the care you received at your last counselor or social worker visit?
1. Extremely satisfied
2. Very satisfied
3. Satisfied
4. Somewhat satisfied
5. Dissatisfied
6. Extremely dissatisfied
7. Don't know (997)
8. Prefer not to say (999)

HCA_10. Thinking about all of your experiences getting healthcare over the **<u>last 6 months</u>**, have you <u>ever</u> felt uncomfortable or thought you had been treated badly?
1. Yes
2. No
3. I have not tried to get healthcare in the last 6 months
4. Don't know (997)
5. Prefer not to say (999)

If HCA_10 = No (2), I have not tried to get healthcare in the last 6 months (3), Don't know (997) (4) OR Prefer not to say (999) (5), skip to HCA_11

HCA_10a. Why do you think you felt uncomfortable or were treated badly? *(Select all that apply)*
1. Your race or ethnicity
2. Your sex or gender
3. Your age
4. Your drug/alcohol use
5. Your income level
6. Your sexual identity
7. You are homeless
8. Other, please specify: _________

If HCA_10a = Other (8), then ask HCA_10a1

HCA_10a1: Other: ______ *(write in)*

HCA_11. Is there a place that you usually go to if you are sick and need healthcare?
1. Yes, there is one place
2. Yes, there is more than one place
3. There is no place
4. Prefer not to say (999)
5. Don't know (997)

If HCA_11 = There is no place (3), Prefer not to say (999) (4), OR Don't know (997) (5) skip to HCA_12;
Else ask

HCA_11a. What kind of place do you go to most often? *Read if necessary: A doctor's office or health center: a place where you see the same doctor or the same group of doctors every visit, where you usually need to make an appointment ahead of time, and where your medical records are on file. Urgent care centers and clinics: places where you do not need to make an appointment ahead of time, and do not usually see the same healthcare provider at each visit.*
1. A doctor's office or health center
2. Walk-in clinic, urgent care center, or retail clinic in a pharmacy or grocery store
3. Emergency room
4. A VA Medical Center or VA outpatient clinic
5. Some other place
6. Does not go to one place most often
7. Prefer not to say (999)
8. Don't know (997)

HCA_12. During the **last 6 months**, was there any time when you needed medical care, but did not get it?
1. Yes
2. No
3. Prefer not to say (999)
4. Don't know (997)

Other Comorbidities

Prompt: We are now going to ask you your height and weight. Research shows that people tend to overestimate their height and underestimate their weight. Please try and give us the most accurate answers possible.

CMD_3. Can you tell me how tall you are without shoes on? *(Feet and inches)*
_______ (*write in* #)
997 Don't know (997)
999 Prefer not to say (999)

CMD_4. Can you tell me how much you weigh?
(ONLY IF QUERIED: 'Without clothes or shoes')
_______ lb (*write in* #)
997 Don't know (997)
999 Prefer not to say (999)

CMD_5:			
Condition	Has a healthcare provider ever told you that you have one or more of the following conditions? (select all that apply)	[If yes to CMD_5*X*1] To the best of your understanding, do you still have [NAME OF PROBLEM]?	[If yes to CMD_5*X*2] In the last 6 months, have you taken or received treatment for [NAME OF PROBLEM]?
Psychiatric/Mental Health			
CMD_5a. ADD/ADHD (Attention-Deficit/Hyperactivity disorder)	CMD_5a1	CMD_5a2	CMD_5a3
CMD_5b. Antisocial personality disorder			
CMD_5c. Bipolar disorder			
CMD_5d. Depression			
CMD_5e. Eating disorders (like anorexia or bulimia)			
CMD_5f. Generalized anxiety disorder			
CMD_5g. Manic and hypomanic episodes			
CMD_5h. Obsessive-compulsive disorder (OCD)			
CMD_5i. Psychosis			
CMD_5j. PTSD (posttraumatic stress disorder)			
CMD_5k. Schizophrenia			

CMD_5:			
Condition	Has a healthcare provider ever told you that you have one or more of the following conditions? (select all that apply)	[If yes to CMD_5*X*1] To the best of your understanding, do you still have [NAME OF PROBLEM]?	[If yes to CMD_5*X*2] In the last 6 months, have you taken or received treatment for [NAME OF PROBLEM]?
CMD_5l. Sleep disorder			
CMD_5m. Social anxiety disorder			
Cardiometabolic Conditions			
CMD_5n. Diabetes			
CMD_5o. Congestive heart failure			
CMD_5p. Heart Disease (history of heart attack, angina)			
CMD_5q. High cholesterol			
CMD_5r. Hypertension/high blood pressure			
CMD_5s. Pre/borderline diabetes			
CMD_5t. Stroke			
Musculoskeletal / Rheumatologic Conditions			
CMD_5u. Arthritis (degenerative/osteoarthritis)			
CMD_5v. Gout			
CMD_5w. Lupus			
CMD_5x. Other Rheumatologic conditions (rheumatoid arthritis,			

CMD_5:			
Condition	Has a healthcare provider ever told you that you have one or more of the following conditions? (select all that apply)	[If yes to CMD_5*X*1] To the best of your understanding, do you still have [NAME OF PROBLEM]?	[If yes to CMD_5*X*2] In the last 6 months, have you taken or received treatment for [NAME OF PROBLEM]?
inflammatory arthritis, sacroiliitis, psoriatic arthritis, etc.)			
Neurologic Conditions			
CMD_5y. Epilepsy/seizure disorder			
CMD_5z. Migraine headaches			
Infectious/Viral Conditions			
CMD_5aa. Hepatitis B			
CMD_5ab. HPV (Human papilloma virus, ano-genital warts)			
CMD_5ac. Viral hepatitis			
Pulmonary/Lung Conditions			
CMD_5ad. Lung disease (asthma, COPD, chronic bronchitis, emphysema)			
Gastrointestinal / Hepatic / Renal Conditions			
CMD_5ae. Inflammatory bowel disease (ulcerative colitis, Crohn's disease)			

**CMD_5:**			
Condition	Has a healthcare provider ever told you that you have one or more of the following conditions? (select all that apply)	[If yes to CMD_5*X*1] To the best of your understanding, do you still have [NAME OF PROBLEM]?	[If yes to CMD_5*X*2] In the last 6 months, have you taken or received treatment for [NAME OF PROBLEM]?
CMD_5af. Kidney problems (polycystic kidney, kidney stones, etc)			
CMD_5ag. Non-viral liver condition (fatty liver, cirrhosis, alcohol/drug-induced hepatitis)			
Endocrine / Metabolic Conditions			
CMD_5ah. Thyroid problems			
Cancer			
CMD_5ai1. Cancer (Type_____)			
CMD_5ai2. Cancer (Type_____)			
CMD_5ai3. Cancer (Type_____)			
Geriatric / Functional Vulnerability			
CMD_5aj. Frailty			

CMD_6. GLP-1 agonists are prescription drugs used for weight loss, diabetes treatment, or to reduce heart disease risk. GLP-1s include drugs like Ozempic, Wegovy, Semaglutide, and others. Have you ever taken GLP-1s for a condition other than for a substance use disorder, which I asked about previously?

1. Yes
2. No
3. Don't know (997)
4. Prefer not to say (999)

IF CMD_6 = 1 (Yes), ask CMD_6a
Else skip to SMK_4

CMD_6a. Are you currently taking GLP-1s for a condition other than a substance use disorder?

1. Yes
2. No
3. Don't know (997)
4. Prefer not to say (999)

CMD_6b. Did or do you take GLP-1s primarily to lose weight or to treat a chronic condition like diabetes or heart disease? *Select all that apply*

1. To lose weight
2. To treat diabetes
3. To reduce heart disease risk
4. To treat some other condition (write in): _________
5. Don't know (997)
6. Prefer not to say (999)

Prompt: Now I am going to ask some quick questions about smoking cigarettes. I'll ask about vaping (e-cigarettes) a bit later.

SMK_4. Have you ever smoked a tobacco cigarette regularly (that is, smoking daily or almost daily) in your lifetime?

1. Yes
2. No
3. Don't know (997)
4. Prefer not to say (999)

If SMK_4 = No (2), Don't know (997) (3), or Prefer not to say (999) (4), skip to SMK_5;
Else ask

SMK_4a. Do you now smoke cigarettes every day, some days, or not at all?

1. Every day
2. Some days
3. I don't smoke anymore at all
4. Don't know (997)
5. Prefer not to say (999)

If SMK_4a = I don't smoke at all anymore (1), skip to SMK_4d;
Else if SMK_4a = Don't know (997) or Prefer not to say (999), skip to SMK_5;
Else ask

SMK_4b. At what age did you start smoking cigarettes regularly?

________*(write in age)*
997. Don't know (997)
999. Prefer not to say (999)

SMK_4c. On average, about how many cigarettes per day have you smoked over the years? (1 pack = 20 cigarettes)

________*(write in number of cigarettes) [validation: between 1-99]*
997. Don't know (997)
999. Prefer not to say (999)

If SMK_4a = Every day (1) or Some days (2), skip to SMK_5;
Else ask

SMK_4d. About how many years did you smoke cigarettes regularly?

________*(write in years)*
997. Don't know (997)
999. Prefer not to say (999)

SMK_4e. When you did smoke regularly, on average about how many cigarettes per day did you smoke? (1 pack = 20 cigarettes)

________(write in number of cigarettes) [validation: between 1-99)
997. Don't know (997)
999. Prefer not to say (999)

Prompt: The next questions are about electronic cigarettes often called e-cigarettes or vapes/vape pens. E-cigarettes may look like regular cigarettes, a pen or a cartridge, but are battery-powered and produce vapor instead of smoke. There are many types of e-cigarettes (e.g., vapes, mods, Juuls).

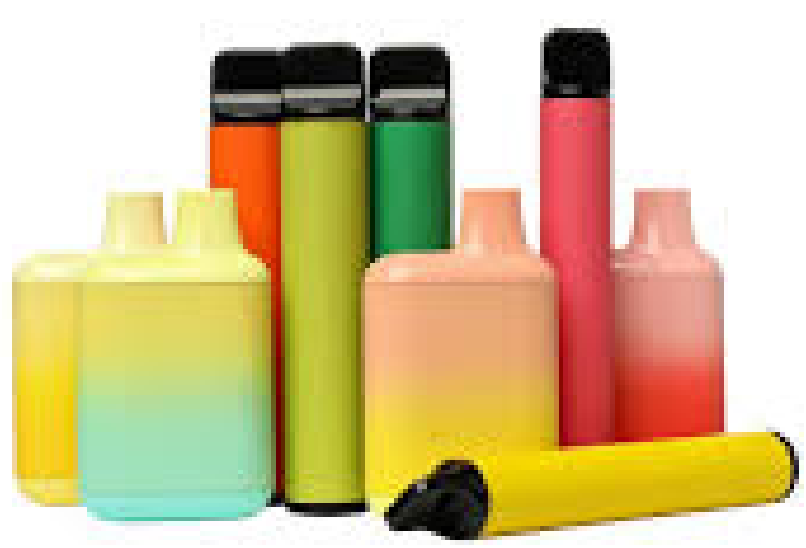

SMK_5. Have you ever used an e-cigarette?

1. Yes
2. No
3. Don't know (997)
4. Prefer not to say (999)

If SMK_5 = No (2), Don't know (997) (3), or Prefer not to say (999) (4), skip to PRG_1;
Else ask

SMK_5a: Do you currently use an e-cigarette?

1. Yes
2. No
3. Don't know (997)
4. Prefer not to say (999)

If SMK_5a = No (2), Don't know (997) (3), or Prefer not to say (999) (4), skip to PRG_1; Else ask

SMK_5b: How many times per day do you usually use your electronic cigarette? (assume that one “time” consists of around 15 puffs or lasts around 10 minutes)

1. 0-4 times per day
2. 5-9 times per day
3. 10-14 times per day
4. 15-19 times per day
5. 20-29 times per day
6. 30+ times per day
7. Don't know (997)
8. Prefer not to say (999)

Pregnancy & Parenting

Prompt: We are going to ask you some questions about parenting. As a reminder, all survey questions are voluntary, and if you would like, we can skip them altogether. Remember that all information collected in this survey is protected by a certificate of confidentiality, which means your identifiable data is protected and we cannot disclose your participation to anyone who is not on the research team.

PRP_1. Do you have any living children under the age of 18 (biological or caregiving role)?

1. Yes
2. No
3. Don't know (997)
4. Prefer not to say (999)

If PRP_1 = 1 (Yes), ask PRP_1a;
Else skip to PRP_4

PRP_1a. How many children do you have that are under the age of 18?
Write in #: _______
997. Don't know
999. Prefer not to say

PRP_2. How many of your children who are under the age of 18 live with you?

1. Write in #: _____ (open box for number)
2. Don't know (997)
3. Prefer not to say (999)

PRP_3. Has child protective services ever been involved with any of your children who are under the age of 18?

1. Yes
2. No
3. Don't know
4. Prefer not to say

If DEM_3 = 1 (Man) and DEM_4 = 2 (No), skip to ORL_1;
Else ask

PRP_4: Have you ever been pregnant?
1. Yes
2. No
3. Don't know (997)
4. Prefer not to say (999)

If PRP_1= 1 (Yes), ask PRP_5;
Else skip to ORL_1

PRP_5: During the **past 6 months**, were you pregnant at any time?

1. Yes, and I am currently pregnant
2. Yes, but I am not currently pregnant
3. No
4. Don't know (997)
5. Prefer not to answer (999)

If PRP_5 = 1 (Yes, and I am currently pregnant), ask PRP_5a;
If PRP_5 = 2 (Yes, but I am not currently pregnant), ask PRP_5b and PRG_5c;
Else skip to PRP_6

PRP_5a: Approximately how many weeks pregnant are you now?

Write in #: ______ (max. 43)
997. Don't know
999. Prefer not to answer

PRP_5b: Did your most recent pregnancy result in a live birth?

1. Yes
2. No
3. Don't know (997)
4. Prefer not to say (999)

PRP_5c: Approximately how weeks pregnant were you when your most recent pregnancy ended?

Write in #: ____ (max 43)
997. Don't know
999, Prefer not to say

PRP_6: Did you get supplies or services from a harm reduction program **during your most recent pregnancy**?

1. Yes
2. No
3. Don't know (997)
4. Prefer not to say (999)

IF PRP_6 = 1 (Yes), ask PRP_6a;
Else skip to PRP_7

PRP_6a: Which of the following did you receive at a harm reduction setting during your most recent pregnancy? (Select all *that apply)*

1. Pregnancy testing
2. Connection to prenatal care
3. Transportation to prenatal appointments
4. Harm reduction staff attending prenatal appointments for support
5. Peer specialist/peer recovery coach specializing in pregnancy services
6. Safer use counseling during pregnancy
7. Birth control planning post-delivery
8. Pregnancy clothing
9. Parenting classes
10. Lactation/breastfeeding support
11. Infant supplies
12. Support navigating child protective services involvement
13. Other, please specify: ________
14. None; I did not receive pregnancy-related services in a harm reduction setting.

PRP_7: During your **most recent** pregnancy, how did your use of harm reduction services change compared to before the start of your pregnancy?

1. Increased a lot
2. Increased somewhat
3. No change
4. Decreased somewhat
5. Decreased a lot
6. I started using harm reduction services during pregnancy
7. I stopped using harm reduction services during pregnancy
8. I did not use harm reduction services before or during pregnancy
9. Prefer not to answer

PRP_8: **During your most recent pregnancy**, did the following factors affect your use of harm reduction services?

		1.	2.	3.	997.	999.
		Made me use services less than before pregnancy	Did not change my use of services in pregnancy	Made me use services more than before pregnancy	Don't know	Prefer not to say
PRP_8a	Fear of child protective services involvement					
PRP_8b	Feeling judged or stigmatized from staff at harm reduction programs					
PRP_8c	Feeling judged or stigmatized from other clients at harm reduction programs					
PRP_8d	Partner/family influence					
PRP_8e	Not knowing whether a harm reduction program serves pregnant people					
PRP_8f	Availability of pregnancy-specific services					
PRP_8g	Needing help connecting to prenatal care					
PRP_8h	Feeling supported by staff at harm reduction programs					
PRP_8i	Feeling supported by other clients at harm reduction programs					
PRP_8j	Knowing other pregnant people also used these services					
PRP_8k	Other ______					

**PRP_9: Perceived Discrimination During Prenatal Care**<br>These questions relate to the medical care given during your most recent pregnancy. Your options are: never, rarely, sometimes, most of the time, always, don't know, and prefer not to say.								
		1.	2.	3.	4.	5.	997.	999.
		Never	Rarely	Sometimes	Most of the time	Always	Don't know	Prefer not to say
PRP_9a	You are treated with less courtesy than other people.							
PRP_9b	You are treated with less respect than other people.							
PRP_9c	You receive poorer service than others.							
PRP_9d	A doctor or nurse acts as if he or she thinks you are not smart.							
PRP_9e	A doctor or nurse acts as if he or she is afraid of you.							
PRP_9f	A doctor or nurse acts as if he or she is better than you.							
PRP_9g	You feel like a doctor or nurse is not listening to what you were saying.							

Oral Health and Access to Dental Services

Prompt: Now, we'll talk quickly about dental health. *NOTE: only ask annually*

ORL_1. When was your last dental check-up?

1. Less than 6 months ago
2. 6-12 months ago
3. More than 1 year but less than 2 years ago
4. 2-5 years ago
5. More than 5 years ago
6. Never
7. Don't know (997)
8. Prefer not to say (999)

If ORL_1 = 6 (Never), skip to ORL_6;
Else ask

ORL_2. What kind of place did you go to for your most recent dental health check up?
Read if necessary: A dentist's office is a stand-alone place where you see the same dentist or the same group of dentists every visit, where you usually need to make an appointment ahead of time, and where your dental records are on file.

1. A dentist office
2. A hospital dental department
3. A health center
4. A VA Medical Center or VA outpatient clinic
5. A mobile dental unit
6. A dental school
7. Some other place
8. Don't know (997)
9. Prefer not to say (999)

If ORL_ 1= 6 (Never) OR 8 (Don't know (997)) OR 9 (Prefer not to say (999)), skip to ORL_6;
Else ask

ORL_3. Thinking about the last time you had a dental check up, how was your overall experience getting treatment?

1. Extremely positive
2. Positive
3. Neutral
4. Negative
5. Extremely negative
6. Don't know (997)
7. Prefer not to say (999)

ORL_4. Thinking about the last time you had a dental check up, how much do you agree that the providers have respected you and your values, beliefs, and feelings?

1. Strongly agree
2. Agree
3. Neutral
4. Disaggree
5. Strongly disagree
6. Don't know (997)
7. Prefer not to say (999)

ORL_5. Overall, how satisfied were you with the care you received at your last dental check up?

1. Extremely satisfied
2. Very satisfied
3. Satisfied
4. Somewhat satisfied
5. Dissatisfied
6. Extremely dissatisfied
7. Don't know (997)
8. Prefer not to say (999)

ORL_6. During the **<u>last 12 months</u>**, was there a time that you needed dental care, but you couldn't or didn't get it at the time?

1. Yes
2. No
3. Don't know (997)
4. Prefer not to say (999)

If ORL_6= 2 (No), 3 (Don't know (997)) or 4 (Prefer not to say (999)), skip to ORL_7; Else ask

ORL_6a. What were the reasons that you could not or did not get the dental care you needed in the **<u>last 12 months</u>**? *(Select all that apply)*

1. I did not think I needed it
2. I am not ready yet
3. Couldn't afford care
4. Not covered by health insurance
5. Could not find treatment or did not know where to go for treatment
6. Transportation issues (e.g., too far away, no public transit available, transit is too expensive)
7. Waitlist was too long (or am on waitlist still)
8. Difficulty scheduling appointments
9. Got incarcerated
10. Turned down by a dental provider

11. Afraid or do not like dentists
12. Other, please specify:_____________
13. Don't know (997)
14. Prefer not to say (999)

If ORL_6a ! = Other (12), then skip to ORL_7;
Else ask

ORL_6a1: Other: ______ *(write in)*

Optional Prompt: The next questions will ask about the condition of your teeth and some factors related to gum health.

ORL_7. Gum disease is common and might cause swollen gums, receding gums, sore or infected gums, or loose teeth. Do you think you might have gum disease currently?
1. Yes
2. No
3. Don't know (997)
4. Prefer not to say (999)

ORL_8. Have you ever had treatment for gum disease, such as scaling and root planning, sometimes called “deep cleaning”?
1. Yes
2. No
3. Don't know (997)
4. Prefer not to say (999)

ORL_9. Do you notice that it looks like your teeth are getting longer?
1. Yes
2. No
3. Don't know (997)
4. Prefer not to say (999)

ORL_10. Have you ever been told by a dental professional or other healthcare provider that you have lost bone around your teeth?
1. Yes
2. No
3. Don't know (997)
4. Prefer not to say (999)

ORL_11. Have you ever had any adult teeth come loose on their own, without an injury?
1. Yes
2. No
3. Don't know (997)
4. Prefer not to say (999)

ORL_12. How many of your adult natural teeth in your mouth are missing (count teeth even if you have dentures to replace them)? If you are not sure, give your best estimate.

_______*(write in #) [validation: between 0-32]*

999. Prefer not to say (999)

If ORL_12 = 0, skip to ORL_14;

Else ask

ORL_13. Do you have a denture/dentures to replace some or all of your missing teeth?

1. All of my teeth
2. Some of my teeth
3. I don't have a denture/dentures
4. Don't know (997)
5. Prefer not to say (999)

ORL_14. Have you ever been told by a health professional that you had a spot on your gums, cheeks, tongue, roof of mouth or throat that needed to be watched?

1. Yes
2. No
3. Don't know (997)
4. Prefer not to say (999)

ORL_15. Have you ever had any tissue in your mouth (that is, on your gums, cheeks, tongue, roof of mouth or throat) biopsied or surgically removed?

1. Yes
2. No
3. Don't know (997)
4. Prefer not to say (999)

ORL_16. How often during the **last year** have you had painful aching anywhere in your mouth?

1. Fairly often
2. Occasionally
3. Hardly ever
4. Never
5. Don't know (997)
6. Prefer not to say (999)

ORL_17. In the **last year**, have you noticed a tooth that doesn't look right (for example, discolored [yellow/brown/grey], abnormally shaped, chipped, or shifted)?

1. Yes
2. No
3. Don't know (997)
4. Prefer not to say (999)

ORL_18. In the **last year**, have you had difficulty doing your usual jobs or attending school because of problems with your teeth, mouth or denture(s)?

1. Yes
2. No
3. Don't know (997)
4. Prefer not to say (999)

ORL_19. In the **last year**, have you had difficulty getting a job because of problems with your teeth, mouth or denture(s)?

1. Yes
2. No
3. Don't know (997)
4. Prefer not to say (999)

ORL_20. In the **last year**, have you had trouble pronouncing any words because of problems with your teeth, mouth or dentures?

1. Yes
2. No
3. Don't know (997)
4. Prefer not to say (999)

ORL_21. In the **last year**, have you found it uncomfortable to eat any foods because of problems with your teeth, mouth or dentures?

1. Yes
2. No
3. Don't know (997)
4. Prefer not to say (999)

ORL_22. Overall, how would you rate the health of your teeth and gums?

1. Excellent
2. Very good
3. Good
4. Poor
5. Fair
6. Don't know (997)
7. Prefer not to say (999)

Mental Health Disorders

Prompt: We're now going to talk about mental health. Some of these questions may make you uncomfortable or upset. Please tell me if you need a break.

**MHD_1: Depression**							
In the last seven days…							
		1.	2.	3.	4.	997.	999.
		Rarely or none of the time (less than 1 day)	Some or a little of the time (1-2 days)	Occasionally or a moderate amount of time (3-4 days)	Most of the time (5-7 days)	Don't know (997)	Prefer not to say (999)
MHD_1a.	I was bothered by things that usually don't bother me.						
MHD_1b.	I had trouble keeping my mind on what I was doing.						
MHD_1c.	I felt depressed.						
MHD_1d.	I felt that everything I did was an effort.						
MHD_1e.	I felt hopeful about the future.						
MHD_1f.	I felt fearful.						
MHD_1g.	My sleep was restless.						
MHD_1h.	I was happy.						
MHD_1i.	I felt lonely.						
MHD_1j.	I could not get "going."						

Optional Prompt: Next, I'll read a statement and you'll tell me how often you've been bothered by any of these problems over the last 2 weeks. We have slightly different options for answers this time.

**MHD_2: Anxiety**							
Over the last 2 weeks, how often have you been bothered by any of the following problems:							
		1.	2.	3.	4.	997.	999.
		Not at all	Several days	More than half the days	Nearly every day	Don't know (997)	Prefer not to say (999)
MHD_2a.	Feeling nervous, anxious, or on edge.						
MHD_2b.	Not being able to stop or control worrying.						
MHD_2c.	Worrying too much about different things.						
MHD_2d.	Trouble relaxing.						
MHD_2e.	Being so restless that it is hard to sit still.						
MHD_2f.	Becoming easily annoyed or irritable.						
MHD_2g.	Feeling afraid, as if something awful might happen.						
MHD_2h.	[If any problems are checked] How difficult have this/these problem(s) made it for you to do your work, take care of things at home, or get along with other people?<br>1. Not difficult at all<br>2. Somewhat difficult<br>3. Very difficult<br>4. Extremely difficult<br>5. Don't know (997)/Prefer not to say (999)						

Optional Prompt: These next statements are asking about the last month…

MHD_3: Perceived Stress Scale								
In the **last month**, how often have you…								
		1.	2.	3.	4.	5.	997.	999.
		Never	Almost Never	Sometimes	Fairly Often	Very Often	Don't know (997)	Prefer not to say (999)
MHD_3a.	Been upset because of something that happened unexpectedly?							
MHD_3b.	Felt that you were unable to control the important things in your life?							
MHD_3c.	Felt nervous and stressed?							
MHD_3d.	Felt confident about your ability to handle your personal problems?							
MHD_3e.	Felt that things were going your way?							
MHD_3f.	Found that you could not cope with all the things that you had to do?							
MHD_3g.	Been able to control irritations in your life?							
MHD_3h.	Felt that you were on top of things?							
MHD_3i.	Been angered because of things that happened that were outside of your control?							
MHD_3j.	Felt difficulties were piling up so high that you could not overcome them?							

Structural & Psychosocial Block

Material Hardship

Prompt: We'll now transition from questions about your individual health to questions about the world around you.

MTH_1. In the **last six months**, how often has finding shelter been a problem for you?
1. Never
2. Rarely
3. Sometimes
4. Usually
5. Don't know (997)
6. Prefer not to answer

MTH_2. In the **last six months**, how often has getting enough to eat been a problem for you?
1. Never
2. Rarely
3. Sometimes
4. Usually
5. Don't know (997)
6. Prefer not to answer

MTH_3. In the **last six months**, how often has obtaining clothes been a problem for you?
1. Never
2. Rarely
3. Sometimes
4. Usually
5. Don't know (997)
6. Prefer not to answer

MTH_4. In the **last six months**, how often has finding a place to wash up been a problem for you?
1. Never
2. Rarely
3. Sometimes
4. Usually
5. Don't know (997)
6. Prefer not to answer

MTH_5. In the **last six months**, how often has finding a place to go to the bathroom been a problem for you?
1. Never
2. Rarely
3. Sometimes
4. Usually
5. Don't know (997)
6. Prefer not to answer

Loneliness

Prompt: Next I have just a few questions for you about your friendships and relationships.

**LNY_1: Loneliness**								
		0	1	2	3	4	997.	999.
		Strongly disagree	Disagree	Neutral	Agree	Strongly agree	Don't know (997)	Prefer not to say (999)
LNY_1a	I am content with my friendships and relationships.	□ 0	□ 1	□ 2	□ 3	□ 4		
LNY_1b	I have enough people I feel comfortable asking for help at any time.	□ 0	□ 1	□ 2	□ 3	□ 4		
LNY_1c	My relationships are as satisfying as I would want them to be.	□ 0	□ 1	□ 2	□ 3	□ 4		

Everyday Discrimination Scale

Prompt: These questions ask about experiences you may have had with discrimination, not just in healthcare settings but in general. In your day-to-day life, how often have any of the following things happened to you?

EDS_1: Everyday Discrimination Scale									
		1.	2.	3.	4.	5.	6	997.	999.
		Almost everyday	At least once a week	A few times a month	A few times a year	Less than once a year	Never	Don't know (997)	Prefer not to say (999)
EDS_1	You are treated with less courtesy or respect than other people								
EDS_2	You receive poorer service than other people at restaurants or stores								
EDS_3	People act as if they think you are not smart								
EDS_4	People act as if they are afraid of you								
EDS_5	You are threatened or harassed								

If EDS_1, EDS_2, EDS_3, EDS_4, OR EDS_5 = (1) Almost everyday, (2) At least once a week, (3) A few times a month, or (4) A few times a year, ask EDS_6
Else skip to CMD_3

EDS_6: What do you think are the main reasons for these experiences? *(Select all that apply)*

1. Your ancestry or national origins
2. Your gender
3. Your race
4. Your age
5. Your religion
6. Your height
7. Your weight
8. Some other aspect of your physical appearance
9. Your housing status
10. Your sexual orientation
11. Your education or income level
12. Your drug/alcohol use
13. A physical disability
14. Your HIV status
15. Other, please specify: _____________

If EDS_6 = Other (Specify) (14), ask EDS_6a;

EDS_6a: Other:______ *(write in)*

Transportation Insecurity

**Prompt: Please rate how often the following have prevented you from going somewhere (e.g., a job, a syringe service program [SSP] or syringe exchange program, doctor’s appointment, groceries) in the last 6 months.**							
TSP_1.		1.<br>Never	2.<br>Rarely	3.<br>Sometimes	4.<br>Often	997.<br>Don't know (997)	999.<br>Prefer not to say (999)
TSP_1a.	The location was too far away to get to easily.						
TSP_1b.	I did not have access to a working car or vehicle available when I needed it.						
TSP_1c.	I could not afford the cost of gas, parking, or public transit fares.						
TSP_1d.	I could not find a friend or family member to give me a ride.						
TSP_1e.	Public transportation (bus, train, etc.) does not run in my area or at the times I need it.						
TSP_1f.	My usual way of getting around was broken, late, or otherwise unreliable.						

Criminal Justice System Involvement

Prompt: Now I have some questions about your encounters with the criminal justice system. Just as a reminder, like we talked about in the consent form, this study is covered by a Certificate of Confidentiality from the National Institutes of Health (NIH). This means that I or the rest of the research team cannot and will not release identifiable data about you to anyone without your consent, even if there is a court order or subpoena.

CJS_1. In the **last six months**, how many times were you stopped by the police (even if it didn't lead to arrest or further legal consequences)? *(Note: this could be anywhere and for any reason)*

______ *(write in #)*

997. Don't know (997)
999. Prefer not to say (999)

If CJS_1 = Zero (0), skip to CJS_4;
Else ask

Prompt: We'd now like to ask you a few questions about the police stop in the last six months that stands out most in your mind. It doesn't need to be the most recent one, but rather the one that you remember the best.

CJS_2.		1.	2.	997.	999.
		Yes	No	Don't know (997)	Prefer not to say (999)
CJS_2a.	Did the officer frisk you, pat you down, or search your bags or pockets?				
CJS_2b.	Did the officer confiscate your drug use equipment/works?				
CJS_2c.	Did the officer use harsh or insulting language?				
CJS_2d.	Did the officer use physical force?				
CJS_2e.	Did the officer treat you fairly?				
CJS_2f.	Did the officer explain why they stopped you in a way that was clear to you?				

CJS_3: Have you been arrested in the **last six months**?

1. Yes
2. No
3. Don't know (997)
4. Prefer not to say (999)

If CJS_3 = No (2) or Don't know (997) (3) or Prefer not to say (999) (4), then skip to CJS_4;
Else ask

CJS_3a: How many times in the **last 6 months** have you been arrested?
______ *(write in #)*
997. Don't know (997)
999. Prefer not to say (999)

CJS_4: In your lifetime, have you ever been in jail or prison for at least a week?
1. Yes
2. No
3. Don't know (997)
4. Prefer not to say (999)

If CJS_4 = 2 (No) or 3 (Don't know (997)) or 4 (Prefer not to say (999)), then skip to CJS_6;
Else ask

CJS_5: In the **last six months**, have you been in jail or prison for at least a week?
1. Yes
2. No
3. Don't know (997)
4. Prefer not to say (999)

If CJS_5 = No (2) or Don't know (997) (3) or Prefer not to say (999) (4), then skip to CJS_6;
Else ask

CJS_5a: How many times in the **last 6 months** have you been in jail or prison for at least a week?
______ *(write in #)*
997. Don't know (997)
999. Prefer not to say (999)

CJS_5b: The last time you were **in** jail/prison, did you receive methadone, buprenorphine, or naltrexone?
1. Yes
2. No
3. Don't know (997)
4. Prefer not to say (999)

CJS_5c: The last time you were **released from** jail/prison, did you receive any of the following at discharge?
1. Referral to a methadone, buprenorphine, or naltrexone program
2. Referral to other drug treatment services
3. A take-home dose/pack of methadone, buprenorphine, or naltrexone
4. Naloxone (Narcan) for overdose reversal
5. Other services, please specify: _______
6. Don't know (997)
7. Prefer not to say (999)

If CJS_5c ! = Other services (5), skip to CJS_6;
Else ask

CJS_5c1: Other services: ______ *(write in)*

CJS_6: At any time in the **last six months**, have you been on probation or parole?

5. Yes
6. No
7. Don't know (997)
8. Prefer not to say (999)

If CJS_6 = No (2), skip to ECV_1;
Else ask

CJS_6a: Have you been on probation or parole in the **last month**?

1. Yes
2. No
3. Don't know (997)
4. Prefer not to say (999)

Perceived Neighborhood Social Cohesion

**Prompt: I am now going to ask some questions about your neighborhood (the area in which you spend the most time).**										
PNS_1.		1.	2.	3.	4.	5.	6.	7.	997.	999.
		Strongly agree	Agree	Somewhat agree	Neither agree nor disagree	Somewhat disagree	Disagree	Strongly disagree	Don't know (997)	Prefer not to say (999)
PNS_1a.	I really feel a part of this neighborhood.									
PNS_1b.	If you were in trouble, there are lots of people in this area who would help you.									
PNS_1c.	Most people in this area can be trusted.									
PNS_1d.	Most people in this area are friendly.									

Proactive Personality Scale

**Prompt: OK great, just a few more questions.**										
PPS_1.		1.	2.	3.	4.	5.	6.	7.	997.	999.
		Strongly agree	Agree	Somewhat agree	Neither agree nor disagree	Somewhat disagree	Disagree	Strongly disagree	Don't know (997)	Prefer not to say (999)
PPS_1a.	If I see something I don't like, I fix it.									
PPS_1b.	No matter what the odds, if I believe in something I will make it happen.									
PPS_1c.	I love being a champion for my ideas, even against others' opposition.									
PPS_1d.	I excel at identifying opportunities.									
PPS_1e.	I am always looking for better ways to do things.									
PPD_1f.	If I believe in an idea, no obstacle will prevent me from making it happen.									

Post-Interview Questions

(Not participant facing, for interviewers only)

Complete after the respondent has departed. Internal feedback for collection only. Do not read items or responses to participants.

PIQ_1. How attentive was the participant during the interview?

5. Very attentive
6. Somewhat attentive
7. Distracted/not very attentive
8. Not attentive (e.g., sleepy/nodding off)

PIQ_2. The participant took a break during the interview

1. Yes
2. No

If PIQ_2 = 1 (Yes), then ask PIQ_2a
Else skip to PIQ_3

PIQ_2a. Approximately how many breaks were needed?
______ *(write in #)*

PIQ_3. The participant wanted to rush through the interview

1. Yes
2. No

PIQ_4. I would rate this interview as:

1. Excellent
2. Good
3. Fair
4. Poor
5. Very Poor

Participant Screening: Script

(Prior to Enrollment)

Hello, my name is [NAME] from [*Brown University / University of Vermont*]. We are conducting a research study about HIV, hepatitis C, and overdose in [*Rhode Island / Massachusetts / Vermont*]. People who join our study will help us by answering questions in a survey.

The research study takes place in person today, and then we will do brief check-ins every month, and then follow-up surveys every 6 months. Today you will be asked to answer questions from a survey for about 60-90 minutes. Then every month, you will be asked to answer questions from a survey for about 5 minutes. This study will go on for many years.

If you qualify, you will receive $80 for today's entry survey, $5 for each 5-minute monthly check-in survey, and $80 for the six-month follow-up survey. In total, you would earn $230 each year you remain in the study. You will only be compensated for the surveys you respond to.

Would you like to answer a few questions to see if you qualify? Your responses are completely confidential. If you qualify, I will review our study consent information with you and provide a copy for you to keep before starting the survey.

If YES, proceed to Section 1B: Participant Screening Questions

If NO, thank individual and end communication

Participant Screening: Questions

1.a. Participant ID ___________

1.b. Interviewer ID ___________

1.1. How old are you? ________ (*write in #, in years*)

1.2. Are you able to complete a survey in English or Spanish?
1. Yes
2. No
3. Don't know (997)/prefer not to answer

1.3. In the past 30 days, have you used any **illegal or unregulated drugs**, like heroin, fentanyl, xylazine, methamphetamine, crack, powder cocaine, or others like magic mushrooms, ecstasy, ketamine, or GHB.
1. Yes
2. No
3. Don't know (997)/prefer not to answer

1.4. In the past 30 days, have you used any prescription pills or medications that were <u>not</u> prescribed to you, that you got from someone else or bought on the street, or that you used in a way other than prescribed (for example, crushing or snorting them)? This could include medications like methadone or buprenorphine, stimulants or ADHD medications, sedatives, or benzodiazepines like Xanax.
1. Yes
2. No
3. Don't know (997)/prefer not to answer

1.5 In the past 30 days, how have you used these drugs? *Check all that apply*
1. Snort
2. Smoke
3. Inject
4. Swallow (consume by mouth)
5. Something else (e.g., booty bump): *write in response:* _________
6. Don't know (997)/prefer not to answer

If 1.5 = 4 AND != 1, 2, 3, or 5, ask 1.5a, else skip to 1.6

1.5a In the past 30 days, are you sure that you *only* used drugs orally, that is, taken them by mouth?
1. Yes
2. No
3. Don't know (997)/prefer not to answer

If 1.5a = 2, go back to 1.5 and ask question again to clarify consumption modalities

1.6. In the past 30 days, have you *only* used cannabis products, tobacco products (including vapes), or alcohol?

1. Yes
2. No
3. Don't know (997)/prefer not to answer

1.7 Do you currently live in Rhode Island, Massachusetts, or Vermont?
1. Yes; Rhode Island
2. Yes; Massachusetts
3. Yes; Vermont
4. No; none of these states
5. Don't know (997)/prefer not to answer

1.8 Do you have any definitive plans to move outside of Rhode Island, Massachusetts, or Vermont in the next year?
1. Yes
2. No
3. Don't know (997)/unsure/prefer not to answer

1.9 Do you identify as living with HIV?
1. Yes
2. No
3. Don't know (997)/unsure
4. Prefer not to say (999)

To qualify, participants must answer ≥18 to 1.1 AND 1 ("Yes") to 1.3 OR 1 ("Yes") to 1.4 AND 1 ("Snort"), (2) "Smoke", 3 ("Inject"), or 5 ("Something else") to 1.5 AND 2 "No" to 1.6 AND 1 ("Rhode Island"), 2 ("Massachusetts", 3 ("Vermont") to 1.7 AND 2 "No" to 1.8. All other participants are NOT eligible.

For participants who do not qualify:

"Unfortunately, you do not qualify for this study at this time. Do I have your permission to collect and save some basic contact information so we could reach out to you about other studies you may qualify for in the future?"
1. Yes
2. No

If Yes, then ask:

What is the best number to contact you? ( ) ______-____________

For participants who qualify:

"Thank you, you qualify for this study. Would you like to continue?"
1. Yes
2. No

If yes, begin protocol for reviewing informed consent documents.

Participant Contact and Scheduling

Prompt: Now I will ask you some personal questions to help us keep in touch with you after today. As described on the consent form, all the information you provide is confidential. Your personal details will be kept separate from your survey responses. We will keep your participation within the study confidential as well as. We will simply state that we are from Brown University or the University of Vermont when we use any of the contact information that you provide.

A. What is your full first name recorded on your birth certificate? ______________________

A1. Do you go by another first name?
  a. Yes
  b. No

A2. ***If yes:*** What other first name do you go by? __________________

B. What is your full last name recorded on your birth certificate? ______________________

B1. Do you go by another last name, like a married name?
  a. Yes
  b. No

B2. *If yes,* what is/was the other last name? __________________

C. What is your date of birth?

___/___/____
mm/dd/year

D. Do you know your social security number?
  a. Yes
  b. No
  c. Don't know (997)/Prefer not to say (999)

D1. If yes, what are the last 4 digits? xxxx____ (write in)

E. Do you currently have access to a phone?
  a. Yes, all of the time
  b. Yes, some of the time
  c. No

E1. *If yes*, what is the best number to contact you? (   ) ______-____________

E1a. *If E1 = yes, is it OK if we leave a voicemail at this phone number?*
  a. Yes
  b. No

ED1b. *If E1 = yes,* do you prefer call or text? ____________

E2. *If E = no*, what is the best way to reach you? ______________________

F. Do you have a current email address that you access regularly?
   a. Yes
   b. No

F1. *If yes*, email: ______________________

Now I am going to ask about contact information for people who might know how to reach you if we are unable to. We will keep your participation within the study confidential as well as. We will simply state that we are from Brown University or the University of Vermont when we use any of the contact information that you provide.

G. Who do you spend most of your time with? ______________________
   G1. Name: ______________________
   G2. Relationship: ______________________

G3. May we contact them if we cannot reach you?
   a. Yes
   b. No

G3a. *If yes*, phone: (   )___-____

H. Do you have any friends that we may contact if we are unable to reach you?
   a. Yes
   b. No

H. *If yes*, Are you comfortable with us contacting your friends in the event that we are unable to reach you?
   a. Yes
   b. No

H1. *If yes*: Name: ______________________ Phone: (   ) ______-____________

H2. *If yes*: Name: ______________________ Phone: (   ) ______-____________

H3. *If yes*: Name: ______________________ Phone: (   ) ______-____________

I. Do you have any family members that we may contact if we are unable to reach you?
   c. Yes
   d. No

I1. *If yes*, Are you comfortable with us contacting your family in the event that we are unable to reach you?
   a. Yes
   b. No

I2. *If yes*: Name: ______________________ Phone: (   ) ______-____________

I3. *If yes*: Name: ______________________ Phone: (   ) ______-____________

J. Do you currently have a case worker?

a. Yes
b. No

J1. *If yes*: Are you comfortable with us contacting your case worker in the event that we are unable to reach you?

a. Yes
b. No

J2. *If yes*: Name: ______________________
Phone: (   ) ______-____________
Organization: ______________________

K. Do you know your most recent address? _______________________________________

K1. *If yes,* do you have a neighbor who would know how to reach you?

a) Yes
b) No

K2. *If yes*: Are you comfortable with us contacting your neighbor in the event that we are unable to reach you?

a. Yes
b. No

K3. *If yes*: Name: ______________________ Phone: (___) ______-____________

K4. Their address (including Apt. #, if relevant) ___________________________________

L. Do you have any social media profiles that you would be comfortable with us using to reach out to you about your next surveys?

a. Yes
b. No

If yes, *ask*:

L1. Facebook: ______________________

L2. Instagram: ______________________

L3. Tiktok: ______________________

L4. Twitter: ______________________

L5. WhatsApp: ______________________

For all responses, 999=Prefer not to say (999); 000=Not applicable

---

Interviewer prompt: Thank you for participating in our survey. Your answers will help us. If you have any remaining questions or concerns, please contact a member of our study team.
