# Supplementary material for: Responding to the increase in substance use, HIV, HCV, and overdose in New England (RISE-NE): Protocol for a prospective cohort study
Source: PLoS One. 2026 Sep 25;21(9):e0358938. doi: 10.1371/journal.pone.0358938 (PMC13614656; doi:10.1371/journal.pone.0358938)
Supplement: Supplementary file 2 [file pone.0358938.s001.docx]

# **Additional File 1:** **Summary of Survey Domains, Item Counts, and Instrument Sources at Enrollment**

# **Table 1. Comprehensive Overview of Survey Domains, Item Counts, and Instrument Sources at Enrollment**

| **Variable Prefix** | **Survey Domain** | **Description** | **Total Number of Items** | **Recall Period(s)** | **Items Informed By…** |
| --- | --- | --- | --- | --- | --- |
| **DEM** | Demographics | Self-reported age, educational attainment, sex assigned at birth, gender identity, sexual orientation, race, ethnicity, language preferences | 15 | Last six months | Developed by our research team |
| **SEP** | Socioeconomic Position | Employment status, income sources, and current housing status | 27 | Last six months, last month | Developed by our research team |
| **DRG** | Drug & Alcohol Use | Alcohol use, frequency of substance use, routes of administration, co-use patterns of multiple substances | 35 | Ever, last year, last six months, last month | PhenX Toolkit* (1)  National HIV Behavioral Surveillance* (2)  AUDIT-C (3) |
| **INJ** | Drug Use (Injection) | Injection practices, source of syringes | 7 | Ever, last month | Developed by our research team |
| **SMK** | Drug Use (Smoking) | Smoking practices, source of pipes | 10 | Ever, last month | Developed by our research team |
| **DGG** | Drug Use (General) | Drug use locations, relationship with seller | 4 | Last month | Developed by our research team |
| **UNX** | Unexpected Drug Use | Experiences with unexpected substance exposure, known/suspected drug adulteration (e.g., xylazine, fentanyl, medetomidine), and use of drug checking resources | 12 | Last month | Informed by existing xylazine literature (4) |
| **SUD** | Substance Use Disorders | Clinical screening criteria for substance use severity | 15 | Last six months | TCU Drug Screen 5* (5) |
| **DUS** | Drug Use Stigma | Perceived stigma and discrimination encountered in healthcare settings | 9 | Last six months | Drug Use Stigma Scale (DUSS)* (6) |
| **OVD** | Overdose | Personal experience of overdose or overamp | 17 | Last six months, last month | Developed by our research team |
| **HRD** | Harm Reduction | Access to, utilization of, and barriers to harm reduction services (e.g., naloxone, safer use supplies, overdose prevention centers, drug checking services, etc.) | 24 | Ever, six months | Developed by our research team |
| **SUT** | Substance Use Disorder Treatment | Access to, utilization of, and barriers to treatment for substance use disorder (e.g., MOUD, outpatient treatment, inpatient treatment, etc.) | 62 | Ever, last six months | Developed by our research team |
| **HCV** | HCV Prevention & Treatment | Experiences with HCV testing, diagnosis, and treatment | 18 | Ever | Developed by our research team |
| **STI** | STI Testing | Experiences with STI testing and treatment, experiences with PEP | 11 | Ever, last six months | Developed by our research team |
| **HTS** | HIV Testing and Prevention | HIV testing experiences, current HIV status, HIV knowledge, HIV risk perceptions, experiences with PrEP, barriers to regular PrEP use, experiences with PEP | 48 | Ever, last six months, last seven days | Developed by our research team |
| **HCC** | HIV Care Cascade | Experiences with HIV care and treatment, medication adherence, HIV stigma | 38 | Ever, last six months, last seven days | HIV Treatment Adherence Self-Efficacy Scale (HIV-ASES) (7)  UNAIDS Indicator Registry — Stigma and Discrimination* (8) |
| **SRB** | Sexual Risk Behaviors | Sexual behaviors, number of sexual partners, partner characteristics, condom use frequency, drug use during intercourse, experiences with transactional intercourse | 14 | Ever, last six months | Developed by our research team |
| **HQL** | Health Status & Quality Of Life | Self-report assessments of general physical health, emotional well-being, and functional limitations over the preceding 30 days. | 12 | Last seven days | EQ-5D Health Questionnaire* (9)  Single Item Sleep Quality Scale (10)  PROMIS® Short Form* (11) |
| **HCA** | Healthcare Access | Access to healthcare services and providers, self-report quality of care | 26 |  | PhenX Toolkit - Access to Health Services* (12) |
| **CMD** | Other Comorbidities | Wound care, experiences with physical and psychiatric illnesses, use of GLP-1s, use of cigarettes and e-cigarettes | 50 | Ever, last six months | Informed by existing wound care literature (13) |
| **PRP** | Pregnancy & Parenting | Reproductive history, service access during pregnancy, perceived discrimination during prenatal care | 29 | Ever, last six months | Perceived Discrimination During Prenatal Care (14) |
| **ORL** | Oral Health & Access to Dental Services | Perceived dental health, emergency dental needs, and access/barriers to dental care | 23 | Ever, last year | Self-Reported Oral Health Questionnaire* (15) |
| **MHD** | Mental Health Disorders | Symptom severity screening tools evaluating clinical components of depression, anxiety, and stress | 28 | Last month, last two weeks, last seven days | CES Depression Scale* (16)  GAD-7 (17)  Perceived Stress Scale (18) |
| **MTH** | Material Hardship | Self-report ability to meet basic survival needs | 5 | Last six months | Existing literature on material hardship (19) |
| **LNY** | Loneliness | Self-report loneliness and isolation | 3 | N/A | Campaign to End Loneliness Measurement Tool (20) |
| **EDS** | Everyday Discrimination | Experiences of discrimination | 6 | Ever | The Everyday Discrimination Scale - Short Form* (21) |
| **TSP** | Transportation Insecurity | Transportational barriers to accessing needed services | 6 | Last six months | Transportation Security Index* (22) |
| **CJS** | Criminal Justice System Involvement | History of police stops, arrests, incarceration, community supervision (probation/parole) | 16 | Ever, last six months, last month | Developed by our research team |
| **PNS** | Perceived Neighborhood Social Cohesion | Perceived social trust and connection within the participant's primary residential or geographic community | 4 | N/A | Perceived Neighborhood Social Cohesion Scale (23) |
| **PPS** | Proactive Personality Scale | Proactive personality scale | 6 | N/A | 6-item Proactive Personality Scale (24) |

** Adapted by investigators*

**References**

1. PhenX Toolkit: Protocols [Internet]. [cited 2026 Jun 8]. Protocol - Substances - 30-Day Frequency. Available from: https://www.phenxtoolkit.org/protocols/view/31302
2. CDC. HIV Data [Internet]. 2026 [cited 2026 Jun 8]. National HIV Behavioral Surveillance (NHBS). Available from: https://www.cdc.gov/hiv-data/nhbs/index.html
3. Bush K. The AUDIT Alcohol Consumption Questions (AUDIT-C)An Effective Brief Screening Test for Problem Drinking. Arch Intern Med. 1998 Sep 14;158(16):1789. doi:10.1001/archinte.158.16.1789
4. Kelly PJA, Green TC, Rich JD, Vento SA, Bailey A, Silva V, et al. Xylazine Awareness and Suspected Presence in the Illicit Drug Supply Among People Who Used Stimulants in an Overdose Hotspot, 2023. Subst Use Misuse. 2026;61(2):217–26. doi:10.1080/10826084.2025.2549501 PubMed PMID: 40888200; PubMed Central PMCID: PMC12826337.
5. Knight DK, Blue TR, Flynn PM, Knight K. The TCU Drug Screen 5: Identifying Justice-involved Individuals with Substance Use Disorders. J Offender Rehabil. 2018;57(8):525–37. doi:10.1080/10509674.2018.1549180 PubMed PMID: 31666789; PubMed Central PMCID: PMC6820856.
6. Pollini RA, Paquette CE, Irvin B, Syvertsen JL, Lilly CL. Drug-related stigma among people who inject drugs – development and validation of the drug use stigma scale (DUSS). PLOS ONE. 2025 Dec 12;20(12):e0338691. doi:10.1371/journal.pone.0338691
7. Johnson MO, Neilands TB, Dilworth S, Morin SF, Remien RH, Chesney MA. The Role of Self-Efficacy in HIV Treatment Adherence: Validation of the HIV Treatment Adherence Self-Efficacy Scale (HIV-ASES). J Behav Med. 2007 Oct;30(5):359–70. doi:10.1007/s10865-007-9118-3 PubMed PMID: 17588200; PubMed Central PMCID: PMC2423379.
8. Stigma and discrimination experienced by people living with HIV in community settings | Indicator Registry [Internet]. [cited 2026 Jun 8]. Available from: https://indicatorregistry.unaids.org/indicator/stigma-and-discrimination-experienced-people-living-hiv-community-settings
9. Rabin R, Charro FD. EQ-SD: a measure of health status from the EuroQol Group. Ann Med. 2001 Jan;33(5):337–43. doi:10.3109/07853890109002087
10. Snyder E, Cai B, DeMuro C, Morrison MF, Ball W. A New Single-Item Sleep Quality Scale: Results of Psychometric Evaluation in Patients With Chronic Primary Insomnia and Depression. J Clin Sleep Med JCSM Off Publ Am Acad Sleep Med. 2018 Nov 15;14(11):1849–57. doi:10.5664/jcsm.7478 PubMed PMID: 30373688; PubMed Central PMCID: PMC6223557.
11. PhenX Toolkit: Protocols [Internet]. [cited 2026 Jun 8]. Protocol - Pain Interference - Adult. Available from: https://www.phenxtoolkit.org/protocols/view/860501
12. PhenX Toolkit: Protocols [Internet]. [cited 2026 Jun 8]. Protocol - Access to Health Services. Available from: https://www.phenxtoolkit.org/protocols/view/270101
13. Kelly PJA, Jessop AB, Scialanca M, Singley K, Luck CC, Hoadley A, et al. Psychosocial Impact of Suspected Xylazine-Associated Skin Wounds Among People Using Fentanyl, Philadelphia, 2022 to 2023. Subst Use Addict J. 2025 Apr;46(2):347–56. doi:10.1177/29767342241289797
14. Prater C, Cohen L, Chau E, Carter EB, Kuebee B, Tepe M, et al. Perceived Discrimination During Prenatal Care at a Community Health Center. J Racial Ethn Health Disparities. 2023 Jun;10(3):1304–9. doi:10.1007/s40615-022-01315-5 PubMed PMID: 35486348; PubMed Central PMCID: PMC9053117.
15. Chou R, Selph SS, Bougatsos C, Nix C, Ahmed A, Griffin J, et al. Table 1, Self-Reported Oral Health Questionnaire [Text] [Internet]. 2023 [cited 2026 Jun 8]. Available from: https://www.ncbi.nlm.nih.gov/books/NBK597294/table/ch3.tab1/
16. Radloff LS. The CES-D Scale: A Self-Report Depression Scale for Research in the General Population. Appl Psychol Meas. 1977 Jun;1(3):385–401. doi:10.1177/014662167700100306
17. Spitzer RL, Kroenke K, Williams JBW, Löwe B. A brief measure for assessing generalized anxiety disorder: the GAD-7. Arch Intern Med. 2006 May 22;166(10):1092–7. doi:10.1001/archinte.166.10.1092 PubMed PMID: 16717171.
18. Harris KM, Gaffey AE, Schwartz JE, Krantz DS, Burg MM. The Perceived Stress Scale as a Measure of Stress: Decomposing Score Variance in Longitudinal Behavioral Medicine Studies. Ann Behav Med Publ Soc Behav Med. 2023 Apr 21;57(10):846–54. doi:10.1093/abm/kaad015 PubMed PMID: 37084792; PubMed Central PMCID: PMC10498818.
19. Goldshear JL, Ganesh SS, Borquez A, Gelberg L, Corsi KF, Bluthenthal RN. Material hardship, forced displacement, and negative health outcomes among unhoused people who use drugs in Los Angeles, California and Denver, Colorado: a latent class analysis. BMC Public Health. 2025 Feb 13;25:591. doi:10.1186/s12889-025-21626-6 PubMed PMID: 39939965; PubMed Central PMCID: PMC11823192.
20. Campaign to End Loneliness [Internet]. 2022 [cited 2026 Jun 8]. Campaign to End Loneliness Measurement Tool — Evaluation and measurement. Available from: https://www.campaigntoendloneliness.org/evaluation/
21. Williams DR, Yan Yu null, Jackson JS, Anderson NB. Racial Differences in Physical and Mental Health: Socio-economic Status, Stress and Discrimination. J Health Psychol. 1997 Jul;2(3):335–51. doi:10.1177/135910539700200305 PubMed PMID: 22013026.
22. Transportation Security Index [The Transportation Insecurity Project]. Univeristy of Michigan [Internet]. 2025. Available from: https://transportation-insecurity.umich.edu/transportation-security-index/
23. Byrnes H, Miller B, Chamratrithirong A, Rhucharoenpornpanich O, Cupp P, Atwood K, et al. The Roles of Perceived Neighborhood Disorganization, Social Cohesion, and Social Control in Urban Thai Adolescents’ Substance Use and Delinquency. YOUTH Soc. 2013 Sep;45(3):404–27. Located at: WOS:000322771000006. doi:10.1177/0044118X11421940
24. Claes R, Beheydt C, Lemmens B. Unidimensionality of Abbreviated Proactive Personality Scales across Cultures. Appl Psychol. 2005 Oct;54(4):476–89. doi:10.1111/j.1464-0597.2005.00221.x
